# iTRAQ-Based Quantitative Proteomic Profiling of *Staphylococcus aureus* Under Different Osmotic Stress Conditions

**DOI:** 10.3389/fmicb.2019.01082

**Published:** 2019-05-29

**Authors:** Tinghong Ming, Lingxin Geng, Ying Feng, Chenyang Lu, Jun Zhou, Yanyan Li, Dijun Zhang, Shan He, Ye Li, Lingzhi Cheong, Xiurong Su

**Affiliations:** ^1^School of Marine Sciences, Ningbo University, Ningbo, China; ^2^College of Food and Pharmaceutical Sciences, Ningbo University, Ningbo, China; ^3^Department of Biological and Environmental Science and Engineering, King Abdullah University of Science and Technology, Thuwal, Saudi Arabia; ^4^Zhejiang Zhengli Antuo Biotechnology Co., Ltd, Ningbo, China

**Keywords:** *Staphylococcus aureus*, osmotic stress, iTRAQ, qRT-PCR, differentially expressed proteins

## Abstract

*Staphylococcus aureus* (*S. aureus*) is an extremely halotolerant pathogenic bacterium with high osmotic stress tolerance, and it is frequently encountered in aquatic production and preservation. However, the mechanism underlying the extremely high osmotic stress tolerance of *S. aureus* remains unclear. In this study, the isobaric tags for relative and absolute quantification (iTRAQ) method was used to identify the differentially expressed proteins (DEPs) under different sodium chloride (NaCl) concentrations. Compared with the control group (0% NaCl), the 10 and 20% NaCl groups had 484 DEPs and 750 DEPs, respectively. Compared with the 10% NaCl group, the 20% NaCl group had 361 DEPs. Among the DEPs, proteins involved in fatty acid synthesis, proline/glycine betaine biosynthesis and transportation, stress tolerance, cell wall biosynthesis and the TCA cycle were upregulated, whereas proteins associated with biofilm formation and pathogenic infections were downregulated. The results obtained in this study indicate that under extremely high osmotic stress, modification of the cell membrane structure, increased biosynthesis and transportation of osmotic protectants, and redistribution of energy metabolism contribute to the osmotic stress tolerance of *S. aureus*, and the infectious ability of the bacteria may be limited. The aim of this study was to provide new insight into how *S. aureus* tolerates the high-salt conditions involved in aquatic production and preservation.

## Introduction

*Staphylococcus aureus* is a gram-positive pathogenic bacterium, and it is the most prevalent cause of food-borne infections worldwide. Approximately 20–30% of the general population carries this symbiotic bacterial pathogen that can result in food poisoning, lead to suppurative infections, acquire drug resistance and cause other medical problems. Thus, *S. aureus* has become a worldwide public health issue (Vandesompele et al., 2002; Argudin et al., 2010; Oteo and Belen Aracil, 2015).

High-salt conditions can be used as an alternative means to preserve foods, and *S. aureus* has higher viability than other bacteria in high-salt foods (McCarthy et al., 2012). Recently, some reports have examined the prevalence of *S. aureus* in fish and fishery products from local retail markets and imported samples in several countries (Obaidat et al., 2015). *S. aureus* is salt-tolerant and therefore can contaminate almost all cured preparations, such as cold-smoked fish, caviar and salt-smoked fish (Moon et al., 2017). Thus, studies on the mechanism of salt tolerance in *S. aureus* aim to provide guidance for the prevention and control of contamination by this bacterium during aquatic production and preservation.

It has previously been shown that under high-salt osmotic stress conditions, bacteria can elicit rapid water flux to dilute or concentrate their cytoplasm, leading to disruption of their structure and function (Romantsov et al., 2009). Importantly, cells can respond to this osmotic stress by actively altering the distribution of specific osmoregulatory solutes through their cytoplasmic membrane (Hohmann, 2002). Osmotic stress tolerance in bacterial cells, to some extent, has been well studied (Munna et al., 2015). *Listeria monocytogenes* responds to hyperosmotic environments by transporting proteins to absorb osmotic protectants such as glycine betaine and carnitine. Similarly, *Bacillus subtilis* can use exogenously provided glycine betaine as an efficient osmoprotectant to adapt to high osmolarity environments (Boch et al., 1994). In addition, *Escherichia coli* and *Salmonella typhimurium* can maintain growth via the high-affinity glycine betaine transport system encoded by the proU operon to accumulate compatible solutes under high osmolarity conditions (Lucht and Bremer, 1994). Additionally, archaea can use organic osmolytes, including ectoines and betaines, to protect their macromolecules and cells against osmotic stress (da Costa et al., 1998). Furthermore, *Salmonella enterica* forms biofilms to alter the susceptibility of the bacteria to exogenous stresses and agents (Hasegawa et al., 2011). Thus, thorough investigation is required to analyze the cause of salt tolerance in *S. aureus* and the underlying mechanism.

Gram-positive *S. aureus* is one of the most halotolerant eubacteria. A previous study reported that *S. aureus* can grow in NaCl concentrations up to 3.5 M (Vijaranakul et al., 1995). Furthermore, when *S. aureus* cells are transferred to highly concentrated NaCl solutions, they can decrease their intracellular turgor by accumulating high concentrations of compatible solutes as well as by increasing their osmotic activity (Csonka, 1989). There have been some studies on *S. aureus* osmoregulation, and glycine betaine, choline, L-proline, and taurine have been shown to act as osmoprotectants (Vijaranakul et al., 1995). Accordingly, other studies have shown that *S. aureus* cells grown in a hyperosmolar environment could accumulate high proline and glycine concentrations (Kunin and Rudy, 1991). Furthermore, the expression levels of various virulence factors are primarily regulated by two-component regulatory systems (TCSs) and the SarA protein family, which regulate the coordinated expression of many genome-wide virulence genes to influence *S. aureus* pathogenicity and biofilm formation (Fournier and Hooper, 2000; Goerke et al., 2005). For instance, the *rbf* gene is involved in the regulation of the multicellular aggregation step of *S. aureus* biofilm formation in response to glucose and salt (Lim et al., 2004). Sihto et al. (2015) used qRT-PCR to assess the impact of NaCl stress on *sed* expression as well as the influence of *agr*, *sarA* and *sigB* on *sed* expression under NaCl stress in *S. aureus*. Similarly, Islam et al. (2015) identified 12 proteins via two-dimensional electrophoresis (2-DE) and mass spectrometry (MS) analyses that demonstrate increased abundance with increasing NaCl concentrations, and they showed that these proteins influence gene regulatory networks that control exopolysaccharide expression. Previous studies have shown that *S. aureus* salt tolerance is very complex and involves various pathways and interconnected networks (Islam et al., 2015). Therefore, the isobaric tag for relative and absolute quantification (iTRAQ) method can be used as a powerful tool for the global investigation of many proteins involved in the osmotic protection mechanism in *S. aureus* under high-salt culture conditions.

**FIGURE 1:**
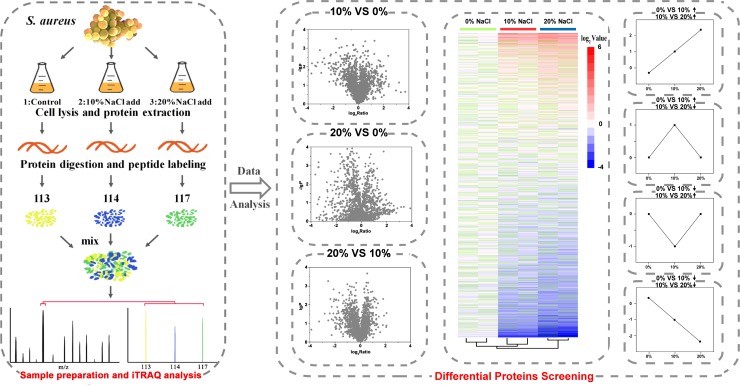
Sample preparation of *S. aureus* for iTRAQ experiments and strategy for performing iTRAQ analysis.

In this study, the cell morphology and structure of *S. aureus* ZS01 cells isolated from pickled aquatic products with 0, 10, and 20% NaCl were observed. The proteomic profiles of *S. aureus* grown in media with different NaCl concentrations were measured via iTRAQ (Figure 1), and qRT-PCR, Gene Ontology (GO), and Kyoto Encyclopedia of Genes and Genomes (KEGG) analyses were carried out for protein complementation, functional annotation, and pathway identification, respectively.

## Materials and Methods

### Bacterial Cultures and Growth Conditions

*Staphylococcus aureus* ZS01 was isolated from a traditional food, pickled *Bullacta exarate*, in Ningbo City, China. Due to the relatively low concentration of *S. aureus*, 10 L of supernatant was collected, filtered and resuspended in medium. Nutrient broth agar medium with 10% NaCl was used to isolate *S. aureus*, and the positive colonies were identified via the API microbial identification system (bioMérieux, Marcy l’Etoile, France) and 16S rRNA sequencing. The salt tolerance of the confirmed *S. aureus* isolates was further retested in nutrient broth agar medium with different NaCl concentrations (15, 20, 25, and 30%). The strain with highest salt tolerance was named *S. aureus* ZS01.

*Staphylococcus aureus* was inoculated into 0.5% (v/v) nutrient broth (NB) medium [Hangzhou Guoyao Group Chemical Reagent (China) Co., Ltd, Hangzhou, China] at 37°C overnight with shaking at 150 rpm. The NB medium included 1% (w/v) each of peptone and beef extract and 0.5% (w/v) of NaCl. Subsequently, the culture was inoculated in 0.5% (v/v) NB medium containing 1% (w/v) each of peptone and beef extract and 0% NaCl (control group), 1% (w/v) each of peptone and beef extract and 10% NaCl, and 1% (w/v) each of peptone and beef extract and 20% NaCl at a 10% inoculum volume. Cell growth was determined by measuring the optical density at 630 nm (OD_630__nm_) and cell counting (CFU/mL) using the 3M^TM^ Petrifilm^TM^ Rapid *S. aureus* Count Plate method. The bacterial strains were grown at 37°C with shaking at 150 rpm for 48 h before harvest (Supplementary Figure S1).

### Transmission Electron Microscopy (TEM)

After incubation for 48 h, all cells were harvested and immediately fixed overnight by 2.5% (v/v) glutaraldehyde. Negative staining and other preparation were performed by the method described as previous described (Vijaranakul et al., 1995). Ultrathin sections stained with uranyl acetate and lead citrate were examined in a HITACHI H-7650 TEM (HITACHI, Tokyo, Japan) operating at 80 kV. The cells were viewed and photographed, and the cell wall thickness was measured by TEM.

### Sample Preparation and Protein Extraction

After 48 h of incubation, liquid cultures were centrifuged at 5,000 rpm for 5 min and washed three times with 1 × PBS (NaCl 8 g⋅L^−1^, KCl 0.2 g⋅L^−1^, Na_2_HPO_4_ 1.44 g⋅L^−1^, KH_2_PO_4_ 0.24 g⋅L^−1^, pH 7.4). The collected *S. aureus* pellets were resuspended in 8 volumes of lysis buffer (4% SDS, 100 mM HEPES, 0.1% PMSF, 1× cocktail) and then incubated in an ice bath ultrasound system (100 W) with the following program: ultrasonic lysis for 5 s followed by a stop for 10 s and then an additional ultrasonic lysis for 5 min. The samples were boiled for 10 min and collected via centrifugation at 14,000 rpm for 30 min at 4°C. The total protein content was quantified using the BCA protein assay kit.

### Protein Digestion, iTRAQ Labeling and Pre-separation

According to the instructions of the iTRAQ kit (Applied Biosystems, Carlsbad, CA, United States), 100 μg of the reduction reagent was added to each sample followed by incubation at 60°C for 1 h. Next, cysteine was added as a blocking reagent. After incubation for 10 min at room temperature, precooled acetone (acetone: sample = 5:1, *v*/*v*) was added to each tube followed by precipitation at −20°C for 1 h. Next, the samples were centrifuged at 12,000 rpm for 20 min at 4°C, and 20 μL of the dissolution solution was added to the precipitate to resuspend and dissolve the sample. Trypsin (Sigma, CA, United States) was added at a ratio of 1:20 (enzyme: protein), and this mixture was incubated overnight at 37°C. Labeling reagent was added to each tube and mixed. After reaction of the samples at room temperature for 1 h, 3 volumes of water were added.

The peptide mixture was then fractionated on a surveyor HPLC system with a 1.7 μm Waters BEH C18 2.1 × 50 mm reversed-phase column (Waters Company, CA, United States). The chromatographic method was as follows: the peptides were eluted with a 5–35% B (A: 20 mM ammonium formate, pH = 10.0; B: acetonitrile) gradient for 16 min. Absorbance was measured at 214 nm, and a total of 40 fractions were collected and then combined into 10 fractions.

### LC-MS/MS Analysis

All fractions were analyzed by nano-HPLC with a 3 μm nano-HPLC C18 250 mm × 75 μm chromatographic column (Eksigent Technologies, Dublin, CA, United States); the A phase was 2% acetonitrile with 0.1% formic acid, and the B phase was 98% acetonitrile with 0.1% formic acid. The peptides were eluted with a gradient of 5–45% for 5–100 min at a flow rate of 300 nL⋅min^−1^. The spray voltage was 2500 V, the mass spectrum acquisition range was 350–1250 m/z, and the total time was 250 ms. The 10 strongest parent ions were taken as a cascade, and the dynamic elimination time was 25 s. Mass spectrometry was performed on a Q Exactive system.

### Protein Identification and Data Analysis

The iTRAQ data were analyzed using Mascot software (version 2.3.02), and the protein identification was performed using the most recently updated UniProt *Staphylococcus aureus* database^1^. iTRAQ modification of peptide/protein identification and quantification was carried out with AB SCIEX ProteinPilot 4.5^TM^. To reduce the probability of false peptide identification, only peptides with an unused ProtScore ≥ 1.3 at the 95% confidence interval as determined via Mascot probability analysis were considered for protein identification and quantification. For the iTRAQ quantitation, the quantitative protein ratios were weighted and normalized to the median ratio in Mascot, and a protein was considered to be differentially expressed if it contained two unique peptides and had similar and significant ratios of ≥1.2 (upregulated) or ≤0.83 (downregulated) in both of the replicates.

### qRT-PCR Complementation

Complementation of the DEPs was performed by quantitative reverse transcription PCR (qRT-PCR). The cells were washed three times with 1 × PBS. After the cells were thoroughly ground in liquid nitrogen, total RNA was extracted using the TransZol Up Plus RNA Kit (Beijing Jinjin Biotechnology Co., Ltd., Beijing, China), according to the manufacturer’s instructions. Next, the RNA yields were determined via a NanoDrop 2000 Spectrophotometer (Thermo Fisher Scientific, Inc., South Logan, UT, United States). One microliter total RNA, 4 μL 5× Mix, and 1 μL DNA Remover were added according to the instructions for the first-strand synthesis kit (Beijing Jinjin Biotechnology Co., Ltd., Beijing, China).

First-strand cDNA was diluted to 30 ng⋅μL^−1^ and used as the template for the qRT-PCR amplification according to the kit instructions (Roche Company, Basel, Switzerland). qRT-PCR was performed in a Rotor-Gene 6000 real-time PCR detection system (Corbett Research, Mortlake, VIC, Australia) coupled with fluorescence signal detection (SYBR^®^ Premix Ex TaqTM II). Each reaction, including four biological replicates, contained a 20.0 μL reaction mixture, which included 10.0 μL SYBR^®^ Premix Ex TaqTM II (2X), 2.0 μL properly diluted cDNA, 1.0 μL each of the forward and reverse primers (10 μM), and 6.0 μL ddH_2_O. The reaction program was as follows: predenaturing at 95°C for 10 s followed by 40 amplification cycles of denaturing at 95°C for 10 s, annealing at 55°C for 10 s, and extension at 72°C for 20 s. The most stable internal reference genes were selected using the software geNorm, and the results were statistically analyzed after normalization of the expression levels of the target genes to the reference genes (Vandesompele et al., 2002; Hellemans et al., 2007). All primers were designed with Primer Premier 5.0, and the primer sequence information is shown in Supplementary Table S1. Each sample was run in triplicate, and all reactions were performed four times. The data analysis was carried out via the 2^−ΔΔCT^ method, followed by statistical analysis with the SPSS software v13.0.

## Results

### High Concentrations of NaCl Inhibit the Cellular Growth of *S. aureus*

**FIGURE 2:**
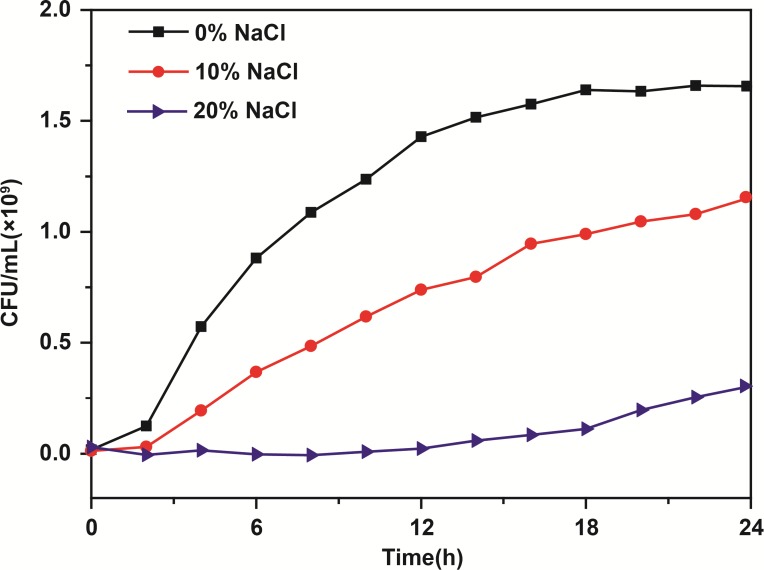
Growth curves of *S. aureus* in culture media containing 0, 10, and 20% NaCl. Data are represented as the means (*N* = 3).

The growth curves of *S. aureus* in 0, 10, and 20% NaCl are shown in Figure 2. From 0 to 3 h, there was no difference in the cell counts in the *S. aureus* cultures with or without NaCl. In the control group, cell growth entered the exponential phase (3–18 h) during which the number of *S. aureus* cells increased stepwise from 0.36 × 10^9^ CFU⋅mL^−1^ to 1.66 × 10^9^ CFU⋅mL^−1^, and then, the cell number remained approximately constant during stationary phase (18–24 h). After incubation for 18 h, the number of *S. aureus* cells slowly and logarithmically increased to 0.80 × 10^9^ CFU⋅mL^−1^ in the 10% NaCl group from the exponential phase to the stationary phase, whereas the number of cells increased to only 0.12 × 10^9^ CFU⋅mL^−1^ in the 20% NaCl group. In addition, after 24 h incubation, the number of *S. aureus* cells reached 1.12 × 10^9^ CFU⋅mL^−1^ in the 10% NaCl group, while cell growth was apparently inhibited in the 20% NaCl group (approximately 0.25 × 10^9^ CFU⋅mL^−1^).

### High Concentrations of NaCl Affect the Cell Wall

**FIGURE 3:**
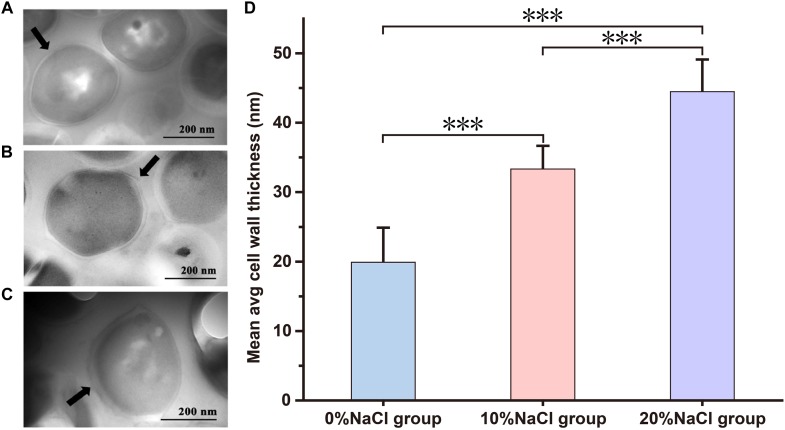
Transmission electron microscopy images of *S. aureus* morphological changes. **(A)** Control group; **(B)** 10% NaCl group; **(C)** 20% NaCl group. **(D)** The cell wall thickness of cells grown in the 0, 10, and 20% NaCl group. ^∗∗∗^*P* < 0.001, the arrow is the cell membrane.

The TEM results showed that high NaCl concentrations had significant effects on the cell wall. Compared with the control group (19.89 ± 4.82 nm, *n* = 25), 10 and 20% NaCl treatments significantly increased the cell wall thickness to 33.34 ± 3.17 nm (*n* = 25, *p <* 0.001) and 44.48 ± 4.46 nm (*n* = 25, *p* < 0.001), respectively (Figure 3 and Supplementary Table S2).

### Differentially Expressed Proteins (DEPs) Identified by iTRAQ

**FIGURE 4:**
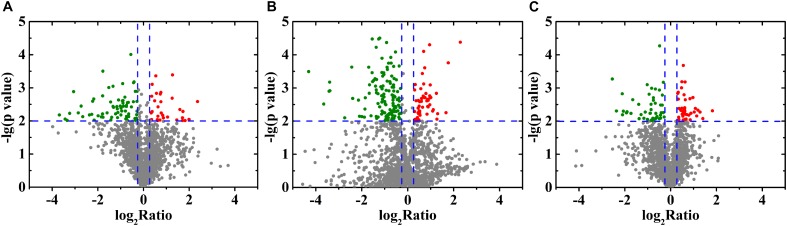
Volcano plot of the DEPs identified in *S. aureus* in response to treatment with 0, 10, and 20% NaCl. Each point represents a protein, and the red and green areas represents upregulated (FC ≥ 1.2 or FC ≤ 0.83 and *P* ≤ 0.05) and downregulated proteins, respectively. **(A)** 10% NaCl group and the control group; **(B)** 20% NaCl group and the control group; **(C)** 20% NaCl group and 10% NaCl group.

In total, 1,820 proteins were identified via the iTRAQ labeling and 2D-LCMS/MS analysis in this study. Detailed information, including the protein quantification data and average iTRAQ ratios, for these identified proteins is shown in Supplementary Table S3. DEPs were defined as proteins with fold changes ≥ 1.2 or ≤0.83 and *p* < 0.05. Compared with the control group, 484 DEPs (194 upregulated and 290 downregulated) and 750 DEPs (279 upregulated and 471 downregulated) were identified in the 10 and 20% NaCl groups, respectively (Figure 4A,B and Supplementary Tables S4, S5). Compared with the 10% NaCl group, 361 DEPs (144 upregulated and 217 downregulated) were identified in the 20% NaCl group (Figure 4C and Supplementary Table S6).

### Hierarchical Cluster Analysis of DEPs

**FIGURE 5:**
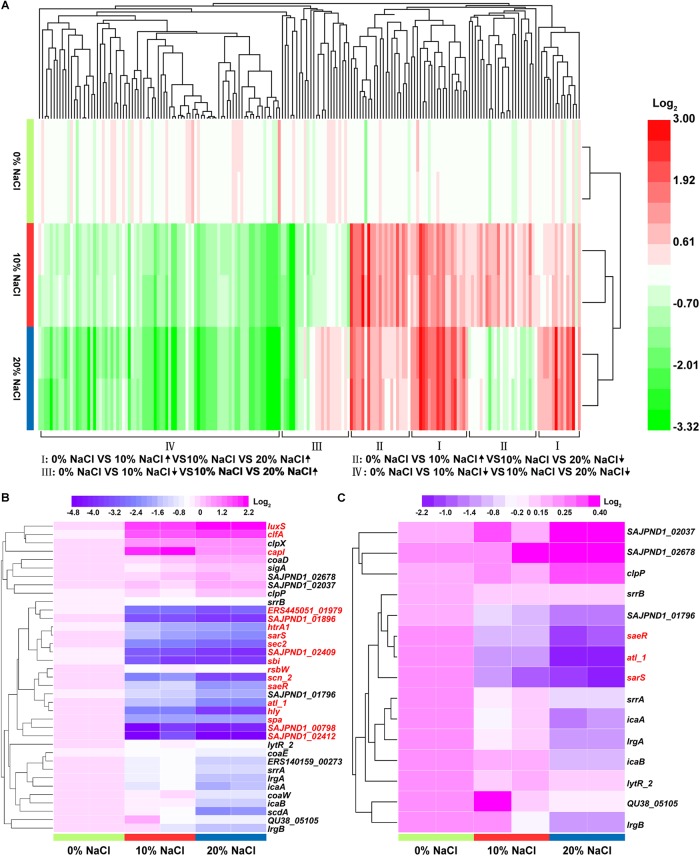
Functional clustering analysis of the DEPs in the three comparison groups. **(A)** Hierarchical cluster analysis of the DEPs with increase/increase, increase/decrease, decrease/increase, and decrease/decrease regulatory patterns; **(B)** DEPs representing virulence factors; **(C)** DEPs representing biofilm formation-related enzymes. The fold-changes of the DEPs are represented by different colors. Red represents upregulated proteins and green represents downregulated proteins. The DEPs with fold changes of ≥ 1.2 or ≤0.83 and *p*-value ≤ 0.05 are labeled in red in Figure 5B,C.

Hierarchical cluster analysis of DEPs was performed using HemI 1.0 (Deng et al., 2014). When the 0, 10, and 20% NaCl groups were considered together, four expression change patterns were identified in the DEPs (Figure 5A). Pattern I (increase/increase): proteins that were stepwise upregulated with increasing NaCl concentrations; Pattern II (increase/decrease): proteins that reached their highest expression levels in the 10% NaCl group; Pattern III (decrease/increase): proteins that reached their lowest expression levels in the 10% NaCl group; and Pattern IV (decrease/decrease): proteins that were stepwise downregulated with increasing NaCl concentrations. The numbers of DEPs that showed each pattern were 20, 59, 20, and 90, respectively (Supplementary Tables S7–S10). In addition, the DEPs involved in virulence and biofilm formation were analyzed in this study, and the levels of these DEPs decreased stepwise in the 10 and 20% NaCl groups compared with their levels in the control group (Figure 5B,C).

### Functional Classification of DEPs Predicted by Gene Ontology (GO) Annotations

**FIGURE 6:**
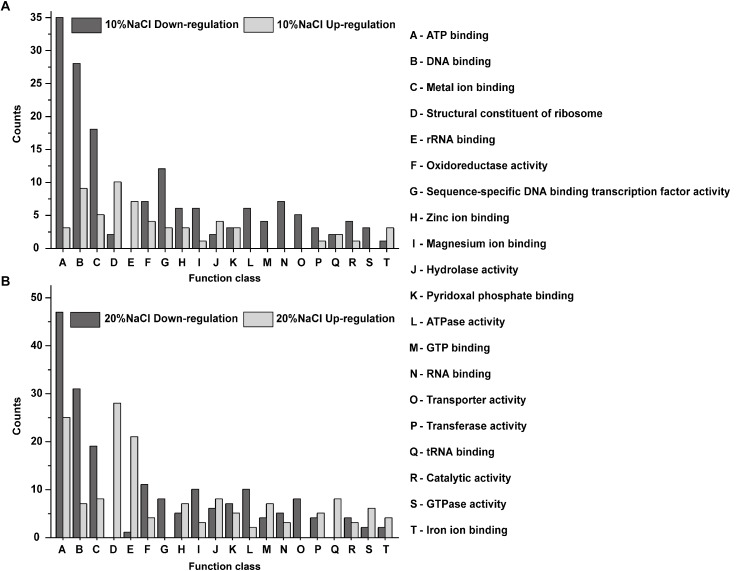
GO functional annotations of the DEPs identified in the 10 and 20% groups compared with their levels in the control. **(A)** DEPs identified between 10% NaCl and control groups; **(B)** GO DEPs identified between 20% NaCl and control groups. The *X*-axis represents the GO categories, and the *Y*-axis represents the number of up- or downregulated DEPs in the functional classification.

Gene ontology analysis was performed using Blast2GO software to identify significantly enriched functional classifications in the DEPs. The DEPs in the 10 and 20% groups were profiled based on their GO functional annotations and were categorized into 20 GO terms. The upregulated DEPs in the 10 and 20% NaCl groups were mainly classified as structural constituent of ribosome, DNA binding, rRNA binding, metal ion binding, ATP binding, tRNA binding and hydrolase activity (Figure 6A). The downregulated DEPs were classified as ATP binding, DNA binding, metal ion binding, RNA binding, sequence-specific DNA binding transcription factor activity, ATPase activity and oxidoreductase activity (Figure 6B).

**FIGURE 7:**
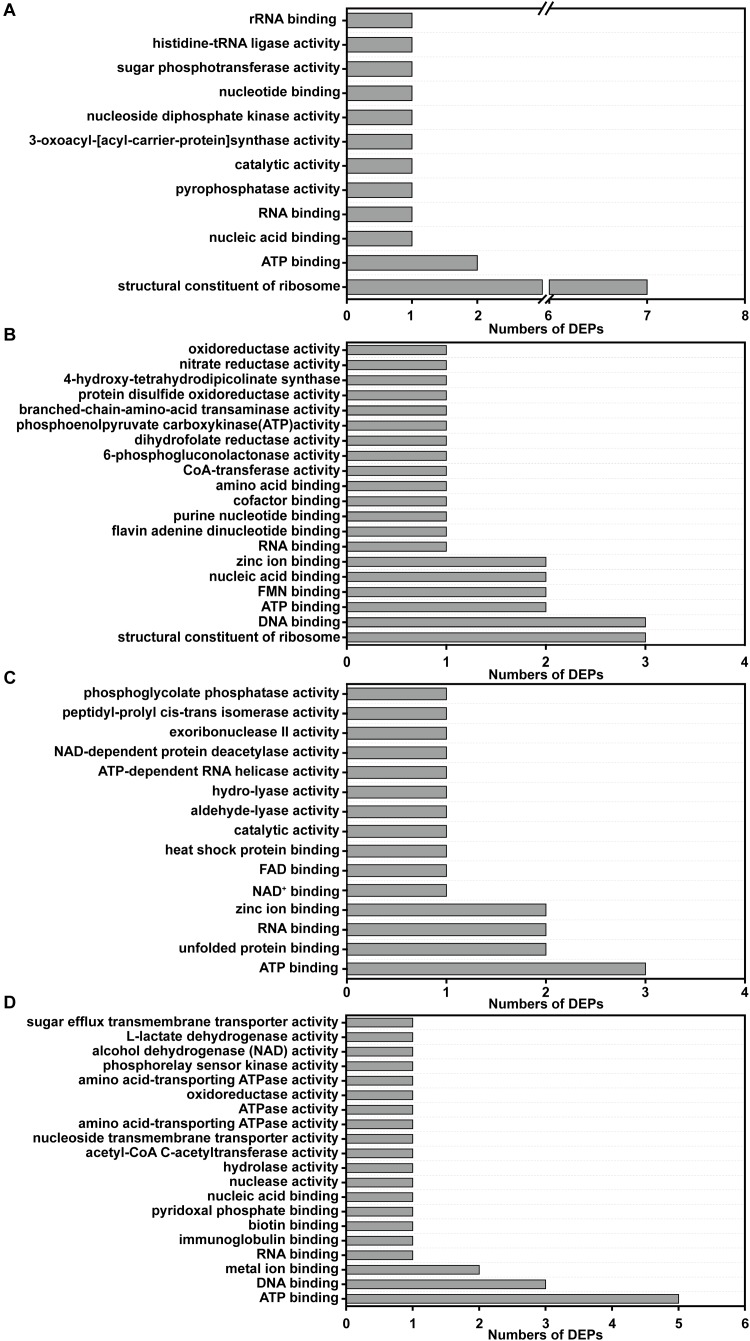
GO functional annotations of the DEPs with different change trends (Pattern I, II, III, and IV). **(A)** The GO functional annotations of the DEPs in pattern I; **(B)** the GO functional annotations of the DEPs in pattern II; **(C)** the GO functional annotations of the DEPs in pattern III; **(D)** the GO functional annotations of the DEPs in pattern IV.

In reference to the four expression change patterns identified in the DEPs, the DEPs involved in pattern I are mainly annotated as structural constituent of ribosome and ATP binding (Figure 7A); the pattern II DEPs are mainly involved in structural constituent of ribosome, ATP binding, DNA binding, FMN binding, nucleic acid binding and zinc ion binding (Figure 7B); the pattern III DEPs are focused on ATP binding, unfolded protein binding, RNA binding and zinc ion binding (Figure 7C); and the pattern IV DEPs are annotated as ATP binding, DNA binding and metal ion binding (Figure 7D).

### Pathways Predicted by the Kyoto Encyclopedia of Genes and Genomes (KEGG)

**FIGURE 8:**
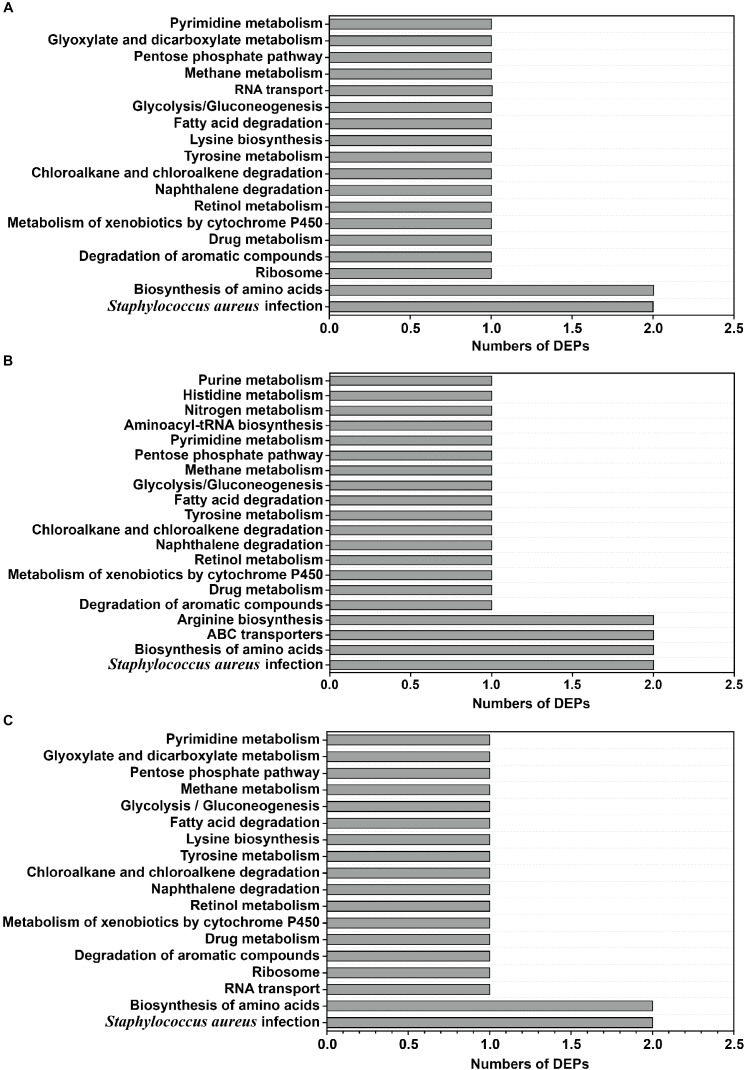
Classification of DEPs according to the KEGG database. **(A)** The significant KEGG pathways were annotated in the 10% NaCl group compared with the control group; **(B)** the significant KEGG pathways were annotated in the 20% NaCl group compared with the control group; **(C)** the significant KEGG pathways were annotated in the 20% NaCl group compared with the 10% NaCl group.

To identify and compare the relevant biological pathways regulated by osmotic stress due to the different NaCl concentrations, the DEPs identified in this study were mapped to the KEGG pathway database ^2^. In total, 24 KEGG pathways were predicted, and all of them were statistically significant in the protein expression profiling study. The groups of DEPs identified in the 10 and 20% NaCl groups were enriched in pathways involved in *S. aureus* infection and biosynthesis of amino acids (Figure 8A,B), whereas pathways involved in ABC transporters and arginine biosynthesis were only found in the 20% NaCl group (Figure 8B). Furthermore, relative to their levels in the 10% group, the DEPs in the 20% group were involved in similar pathways, including *S. aureus* infection and biosynthesis of amino acids (Figure 8C).

### Complementation of the Differentially Expressed Proteins via qRT-PCR Analysis

**FIGURE 9:**
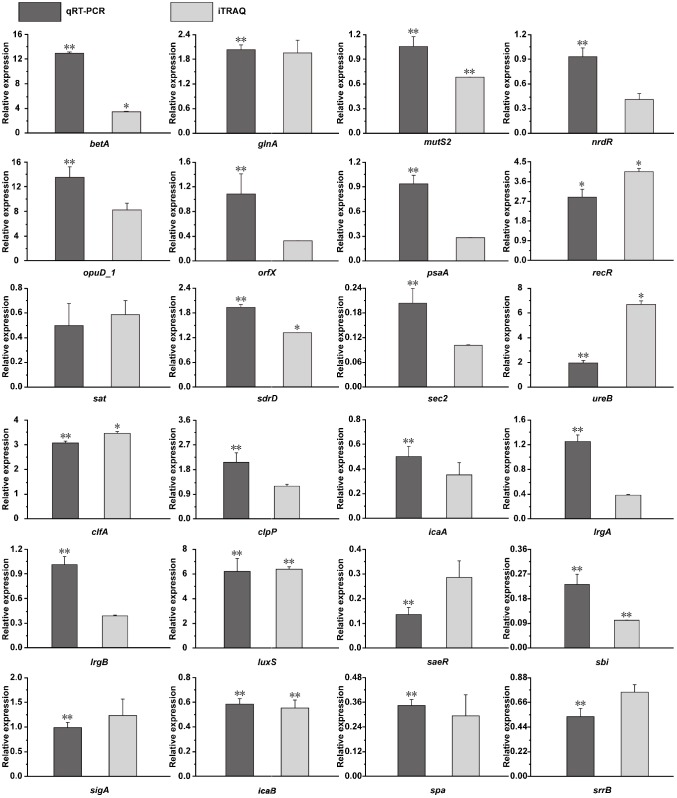
Transcriptional complementation of the proteomic output using qRT-PCR assays. Candidate genes mainly included oxygen-dependent choline dehydrogenase (*betA*), glutamate-ammonia ligase activity (*glnA*), endonuclease activity (*mutS2*), transcriptional repressor (*nrdR*), glycine betaine transporter (*opuD_1*), ribosomal RNA large subunit methyltransferase H (*orfX*), ABC transporter substrate-binding protein (*psaA*), recombination protein (*recR*), serine acetyltransferase (*sat*), LPXTG surface protein (*sdrD*), enterotoxin C2 (fragment) (*sec2*), urease activity (*ureB*), clumping factor A (*clfA*), ATP-dependent Clp protease proteolytic subunit (*clpP*), *N*-glycosyltransferase (*icaA*), antiholin-like protein (*lrgA*), antiholin-like protein (*lrgB*), *S*-ribosylhomocysteine lyase activity (*luxS*), response regulator (*saeR*), immunoglobulin-binding protein (*sbi*), RNA polymerase sigma factor (*sigA*), poly-beta-1,6-*N*-acetyl-D-glucosamine *N*-deacetylase (*icaB*), immunoglobulin G-binding protein A (*spa*), and histidine kinase (*srrB*). The value of the fold change in the *Y*-axis indicates the ratios of DEPs (dark gray bar) or mRNA level (light gray bar) and are shown in the 20% NaCl group compared with the control group. ^∗^*p* < 0.05, ^∗∗^*p* < 0.01, compared with the corresponding control group in the RNA and protein levels.

To verify the correspondence between transcript level and protein expression level, the transcriptional levels of 24 DEPs were measured by qRT-PCR. As shown in Figure 9, 20 genes showed similar trends the changes in their transcript and protein expression levels, whereas only four genes (*mutS2*, *orfX*, *lrgA*, and *lrgB*) show the opposite trend.

## Discussion

*Staphylococcus aureus* remains one of the leading causes of pathogenesis in aquatic production and preservation under high salt condition throughout the world. The mechanism underlying its extremely high osmotic stress tolerance remains frustratingly unclear in spite of several emerging omics-based technologies that could be useful for its elucidation (Sihto et al., 2014). In this study, we performed a comprehensive evaluation of the proteomic profiles of *S. aureus* cells treated with different NaCl concentrations, thus providing new data on osmotic stress tolerance.

### Effects of Osmotic Stress on Cellular Osmotic Protection and Biofilm Formation

Bacterial cell membranes can maintain cellular integrity upon exposure to mechanical, physical, nutritional and chemical stress and can provide resistance to the host immune system to maintain normal bacterial growth (You et al., 2014). Fatty acids, an important component of the cell membrane, can participate in the formation of malonyl-CoA via the catalytic activity of acetyl-CoA carboxylase, which is encoded by the *accA*, *accB*, *accC*, and *accD* genes (Davis et al., 2000). In this study, the AccB, AccC, and AccD (*p* < 0.05) proteins were upregulated in the 10% NaCl group and significantly further upregulated in the 20% NaCl group (*p* < 0.01 for protein AccD) compared to their levels in the control group (Supplementary Tables S3, S4). Published results have indicated that the acetyl-coenzyme A carboxylase (ACC) enzyme family could regulate fatty acid biosynthesis and oxidation, which could be associated with bacterial tolerance of NaCl stress (Xian et al., 2013). Therefore, these results indicate that the high expression level of the *acc* protein family might play key roles in the synthesis of fatty acids involved in preserving the structural integrity of cells and in protecting cells in the microenvironment from direct damage under high-salt osmotic stress conditions.

**FIGURE 10:**
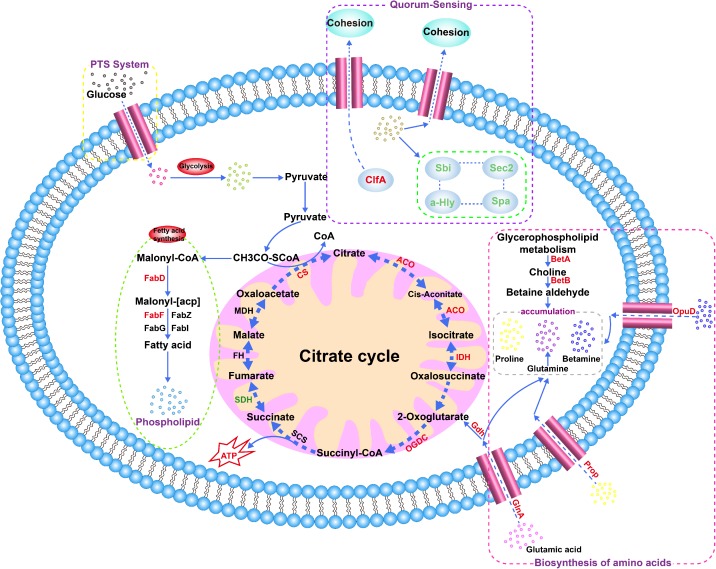
Relevant metabolic pathways and the accumulation of major intracellular osmoprotectants in *S. aureus*. Red font indicates upregulated proteins, and green font indicates downregulated proteins. Candidate proteins include oxygen-dependent choline dehydrogenase (BetA), betaine-aldehyde dehydrogenase (BetB), glutamine synthetase (GlnA), proline/betaine transporter (ProP), and glycine betaine transporter (OpuD), citrate synthase (CS), glutamate dehydrogenase (Gdh), isocitrate dehydrogenase (IDH), aconitate hydratase (ACO), oxoglutarate dehydrogenase complex (OGDC), 3-oxoacyl-[acyl-carrier-protein] synthase 2 (FabF), and malonyl CoA-acyl carrier protein transacylase (FabD).

A previous study reported that *S. aureus* cells produce carbohydrates, alcohols and amino acids to maintain their intracellular osmotic pressure by regulating the expression levels of select intracellular proteins (Price-Whelan et al., 2013). Consistent with our results, proline/betaine transporter (Prop) and glycine betaine transporter (OpuD_1) were upregulated by 2.10− (*p* > 0.05) and 7.61-fold (*p* > 0.05), respectively, in the 20% NaCl group compared with their levels in the control group (Supplementary Tables S3, S5), and the transcriptional level of the *opuD_1* gene increased by 12.48-fold as shown via qRT-PCR (*p* < 0.01) (Figure 9). A recent study showed that the accumulation of compatible solutes, such as glycine betaine, maintain the intracellular osmotic potential to allow cells to resist changes in osmotic pressure and to decrease dehydration, to maintain the stability of their intracellular proteins and nucleic acids, and to preserve their membrane integrity (Calderon et al., 2004). In addition, the Prop and OpuD_1 protein were also upregulated by 1.40− (*p* > 0.05) and 3.30-fold (*p* > 0.05), respectively, in the 10% NaCl group compared with their levels in the control group (Supplementary Tables S3, S4). In addition, as shown in Figure 10, *S. aureus* uses oxygen-dependent choline dehydrogenase (BetA) and betaine aldehyde dehydrogenase (BetB) to accumulate betaine and modulate osmotic pressure from the intracellular environment under hyperosmotic culture conditions (Peddie et al., 1999). It has been reported that BetB catalyzes irreversible NAD(P)-dependent oxidation of betaine aldehyde or glycine betaine aldehyde to glycine betaine, which then functions as an osmoprotectant in both prokaryotic and eukaryotic organisms (Chen et al., 2014). In the present study, BetA and BetB were upregulated 3.00− (*p* < 0.01) and 3.75-fold (*p* > 0.05) in the 10% NaCl group, respectively, and further upregulated 3.60− (*p* < 0.05) and 5.30-fold (*p* > 0.05) in the 20% NaCl group, respectively, compared with their levels in the control group (Supplementary Tables S3–S5). Consistently, the transcriptional level of *betA* also increased significantly (*p* < 0.01) (Figure 9). Taken together, these results indicate that *S. aureus* cells adapt to survive in high-salt osmotic environments by increasing the biosynthesis and conversion of osmoprotectants, such as betaine.

As shown in Figure 5C, upon increasing NaCl concentrations, most of the proteins involved in biofilm formation were downregulated. Our results also show that the IcaA (0.40-fold, *p* < 0.001), IcaB (0.54-fold, *p* > 0.05) and Spa (0.29-fold, *p* < 0.05) proteins were downregulated in the 20% NaCl group (Supplementary Tables S3, S5) and that the transcriptional levels of the genes encoding these proteins were significantly decreased by 0.50− (*p* < 0.01), 0.60− (*p* < 0.01) and 0.30-fold (*p* < 0.01), respectively (Figure 9). Transcription of the Ica operon and Spa can be regulated by the SrrAB TCS; however, this system is inhibited in osmotic stress environments. Moreover, Spa, a bacterial cell surface protein, promotes bacterial growth and virulence as well as regulates a regulatory network involved in quorum-sensing (Figure 10) (Pragman et al., 2004). In addition, the expression levels of the LytR_2 (*p* > 0.05) and LrgA (*p* > 0.05) proteins decreased in the 20% NaCl group (Supplementary Tables S3, S5). It has been reported that these two proteins (which belong to the LytRS TCS family) can not only sense changes in the bacterial cell membrane potential but also inhibit biofilm formation by limiting cell membrane hydrolase activity by regulating the expression of the *lrgA* gene (Chatfield et al., 2005). These results indicate that regulation of biofilm formation plays vital roles in osmotic stress tolerance.

ClpX was upregulated by 1.73− (*p* < 0.05) and 2.39-fold (*p* < 0.05) in the 10 and 20% NaCl groups, respectively, while ClpP was upregulated 1.73-fold (*p* > 0.05) in the 20% NaCl group (Supplementary Tables S3–S5). Both ClpX and ClpP belong to the Clp protease family, a class of Clp ATPases that play crucial roles in stress tolerance, intracellular replication and biofilm formation in *S. aureus* (Frees et al., 2003). Thus, our results reveal that the abundance of the Clp protease family in *S. aureus* is increased in response to salt stress. The SaeR response regulator was downregulated 0.50− (*p* > 0.05) and 0.30-fold (*p* < 0.01) in the 10 and 20% NaCl groups, respectively (Supplementary Tables S3, S4), while the mRNA expression level of its gene was decreased 0.14-fold (*p* < 0.01) in the 20% NaCl group (Figure 9). It has been reported that SaeR is the response regulator of SaeRS TCS, which is associated with regulating bacterial autolysis and biofilm formation (Chen et al., 2013). These results suggest that the growth of *S. aureus* cells can be adapted to cope with high salt stress conditions by regulating the activities of the enzymes in the Clp protease family, whereas the SaeRS TCS is inhibited with respect to their roles in biofilm formation.

Cold shock protein (CspA) and immunodominant staphylococcal antigen A (IsaA) were upregulated by 2.33− (*p* > 0.05) and 1.83-fold (*p* < 0.05), respectively, in the 10% NaCl group compared with their levels in the control group, whereas they were downregulated by 0.68− (*p* > 0.05) and 0.47-fold (*p* < 0.05), respectively, when the NaCl concentration was increased to 20% (Supplementary Tables S3, S4, S6). Previous studies showed that CspA and IsaA play important roles in stabilizing biofilms and that these two proteins were more abundant at higher NaCl concentrations (up to 4%) (Islam et al., 2015). However, at an extremely high NaCl concentration (20%), their functions and the trends in the changes in their expression levels have not been measured before. In contrast, previous studies indicated that reduced CspA levels lead to bacterial aggregation, which is consistent with the results obtained in our 10 and 20% NaCl groups. Therefore, we propose that the regulation of CspA and IsaA, with respect to their roles in biofilm stabilization, is only effective at a low NaCl concentration (10%) rather than at an extremely high NaCl concentration (20%).

### Effects of Osmotic Stress on the Transport and Biosynthesis of Cellular Substances

In this study, most of the proteins related to cellular substance transport were downregulated upon NaCl treatment. Among them, several proteins associated with ABC transporters, such as PsaA (*p* > 0.05), StpA (*p* > 0.05 and *p* < 0.01), SitB (*p* < 0.05 and *p* > 0.05) and UhpT (*p* > 0.05), were downregulated in the 10 and 20% NaCl groups compared with their levels in the control group (Figure 6 and Supplementary Tables S3–S6). Previous studies have shown that in prokaryotes, ABC transporters can utilize energy derived from adenosine triphosphate hydrolysis to mediate the absorption of amino acids and carbohydrates (among other substances) to maintain intracellular or extracellular osmotic balance and bacterial substance transport (Davidson and Chen, 2004). Furthermore, the membrane protein (MmpL8) was upregulated by 1.55-fold (*p* < 0.01) in the 10% NaCl group compared with its level in the control group (Supplementary Tables S3, S4) and by 2.48-fold (*p* < 0.001) in the 20% NaCl group compared with its level in the 10% NaCl group (Supplementary Tables S3, S6). Converse et al. reported that in *Mycobacterium tuberculosis*, MmpL8 (a member of a family of lipid transporters) likely exports lipids from the cytoplasmic membrane to the cell wall (Converse et al., 2003). Such transport may explain why *S. aureus* forms biofilms at high concentrations of NaCl (Islam et al., 2015). Furthermore, MmpL8 upregulation is the most likely explanation for the thicker cell walls observed in *S. aureus* cells grown in 20% NaCl compared with those of cells grown in 10% NaCl, and this conclusion is consistent with those of several previous reports (Kanemasa et al., 1974; Hajmeer et al., 2006). SceD was upregulated 1.75-fold (*p* < 0.01) in the 10% NaCl group compared with its level in the control group (Supplementary Tables S3, S4) and 4.71-fold in the 20% NaCl group compared with its level in the 10% NaCl group (*p* < 0.01) (Supplementary Tables S3, S6). These results are consistent with those in the previous report from Stapleton et al. (2007), who demonstrated that SceD is robustly upregulated by the presence of NaCl and that it plays a fundamental role in peptidoglycan emodeling.

On the other hand, 3-hexulose-6-phosphate synthase (CH51_02975), which is involved in amino acid biosynthesis, was upregulated by 2.40− (*p* > 0.05) and 4.90-fold (*p* > 0.05) in the 10 and 20% NaCl groups, respectively (Supplementary Tables S3–S5), whereas ornithine carbamoyl transferase (SAKOR_01090), which is associated with arginine biosynthesis, was downregulated by 0.30-fold (*p* > 0.05) in the 20% NaCl group (Supplementary Tables S3, S5). In addition, the molecular chaperone DnaK, which plays vital roles in maintaining cell membrane structure, composition and synthesis (Sienczyk et al., 2004), was downregulated by 0.58-fold (*p* < 0.05) in the 10% NaCl group, whereas it was upregulated by 1.79-fold (*p* < 0.05) in the 20% NaCl group (Supplementary Tables S3, S4, S6). There are various ways by which cells can adapt to high-salt osmotic stress. Considering that the response to osmotic stress consumes primary metabolites and energy, cells will use only the most appropriate pathway. Therefore, the contrasting patterns observed in some proteins in this study might be caused by shifts between coping strategies under different NaCl concentrations due to various reasons required to maintain a balance between effectiveness and economy. Taken together, the activities of these proteins in cellular substance transport and amino acid metabolism play major roles in intracellular decomposition and amino acid synthesis for the protection *S. aureus* cells under osmotic stress.

### Effects of Osmotic Stress on Cellular Energy Metabolism

Previous studies have shown that cells possess a self-protective mechanism against exposure to hyperosmotic environments that reduces unnecessary energy loss to help them focus on survival (Lee et al., 2013). In our study, alcohol dehydrogenase (Adh) was significantly downregulated by 0.29-fold (*p* > 0.05) and 0.14-fold (*p* > 0.05) in the 10 and 20% NaCl groups, respectively (Supplementary Tables S3–S5). Adh is closely associated with the cellular energy metabolism pathway, indicating that energy metabolism is likely affected by salt stress. It is well known that the tricarboxylic acid (TCA) cycle is the primary glycolytic pathway for glucose metabolism and that it plays a decisive role in organismal survival (Mailloux et al., 2007).

In this study, several proteins involved in the TCA cycle, including citrate synthase (CS), glutamate dehydrogenase (Gdh), isocitrate dehydrogenase (IDH), aconitate hydratase (ACO), and the oxoglutarate dehydrogenase complex (OGDC), were upregulated in *S. aureus* cells treated with different NaCl concentrations, while succinate dehydrogenase flavoprotein subunit (SdhA_1) was downregulated (Supplementary Tables S3–S6). Citrate synthase catalyzes the first reaction in the TCA cycle and Gdh is involved in the production of α-ketoglutaric acid from glutamic acid, while IDH catalyzes the biosynthesis of TCA cycle precursors, and all three of these proteins play important roles in energy production and carbon assimilation. In addition, 3-oxoacyl-[acyl-carrier-protein] synthase 2 (FabF) and malonyl CoA-acyl carrier protein transacylase (FabD), which are involved in fatty acid biosynthesis and play prominent roles in transferring the malonyl moiety from coenzyme A to acyl-carrier protein, were upregulated after NaCl treatment (Supplementary Tables S3–S6). All of these results indicate that energy metabolism regulation contributes to the osmotic pressure tolerance analyzed in this study.

### DEPs Involved in the Osmotic Stress Response

Studies have shown that osmotic stress may result in the production of a variety of stress proteins that function in cellular self-protection, nucleic acid repair, degradation of abnormal proteins, and regulation of intracellular and extracellular osmotic pressure (Vilhelmsson and Miller, 2002; Kiran and Balaban, 2009). The DnaJ chaperone protein was downregulated by 0.80-fold (*p* > 0.05) in the 10% NaCl group, while it was upregulated by 1.53-fold (*p* > 0.05) in the 20% NaCl group (Supplementary Tables S3–S5). DnaJ plays a central role in maintaining intracellular protein homeostasis and in improving cell membrane fluidity under osmotic stress conditions (Sienczyk et al., 2004; Warnecke, 2012). The proteins UreC and UreG were upregulated by 3.00− (*p* > 0.05) and 3.95-fold (*p* < 0.05), respectively, in the 20% NaCl group, while UreB and UreE were upregulated in both the 10 and 20% groups (Supplementary Tables S3–S5). Increased expression of these four proteins, which are all ureases, contributes to the maintenance of homeostasis in *S. aureus* under hyperosmotic conditions (Jiang et al., 1997). As shown in Figure 10, immunoglobulin-binding protein (Sbi) was downregulated by 0.22− and 0.11-fold in the 10 and 20% NaCl groups (*p* > 0.05), respectively (Supplementary Tables S3–S5). Burman et al. (2008) demonstrated that Sbi is a cell surface adhesion protein associated with electrostatic interactions in *S. aureus*, and that it plays a prominent role in bacterial adhesion.

Finally, gamma-hemolysin A subunit (HlgA) and gamma-hemolysin B subunit (HlgB) were both downregulated in the 10 and 20% NaCl groups (*p* > 0.05) (Supplementary Tables S3–S5). A previous study demonstrated that the Hlg proteins are broadly conserved among *S. aureus* strains and that they play crucial roles during *S. aureus* infection (Delfani et al., 2015). Alpha-hemolysin (Hly) was downregulated by 0.28-fold (*p* < 0.01) and 0.42-fold (*p* > 0.05) in the 10 and 20% NaCl groups, respectively (Supplementary Tables S3–S5). Hly is a potent cytotoxin and is closely linked to the pathogenesis of staphylococcal diseases (Wilke and Bubeck Wardenburg, 2010). Staphyloxanthin biosynthesis protein (CrtP) was downregulated by 0.73− (*p* < 0.05) and 0.71-fold (*p* > 0.05) in the 10 and 20% NaCl groups, respectively (Supplementary Tables S3–S5). Antonic et al. (2013) reported that staphyloxanthin is one of several virulence factors that play crucial roles in the ability of *S. aureus* to withstand and survive stressful environments. These results suggest that high NaCl concentrations reduce the expression levels of proteins related to pathogenic infections and virulence factors in *S. aureus*.

Fold-changes of ≥1.2 or ≤0.83 and *p* < 0.05 were used to identify DEPs in this study instead of the more common cutoffs based on fold-changes of ≥2 or ≤0.5 with *p* < 0.05. We chose these modest cutoffs for two reasons. First, cells of the standard strain grown under normal conditions (∼5–10% NaCl) must robustly alter their metabolism to survive in a high salt environment (>20% NaCl), leading to significant fold-changes in mRNA and protein levels. However, the ZS01 strain used in this study was isolated from pickled *Bullacta exarate*; therefore, it already had a high salt tolerance (>20% NaCl). Subsequent resequencing of its genome identified various variations (SNPs and Indels) in this strain (data not shown) that likely provide more efficient mechanisms for salt tolerance, and which do not change under different osmotic stress conditions. Therefore, ZS01 may not need to extensively alter its gene transcription and protein expression levels to survive in high salt conditions. If we used the default cutoffs, few DEPs would have been identified in this study. Second, some important proteins, such as IcaB, SaeR and CspA, are known to play important roles in osmotic stress tolerance; however, their fold changes detected in this study were 0.54, 0.50, and 0.70, respectively. If the fold-change cutoff of ≤0.5 was used in this study, these three proteins would not have been identified, and other important proteins might also have been missed. Therefore, we decided that the common cutoff was not appropriate for this study, and we choose modest cutoff based on the specific characteristics of the *S. aureus* strain used in this study (ZS01).

The expression level changes of the DEPs identified in this study were complemented via qRT-PCR as previously described (Dong et al., 2016; Hou et al., 2016; Wang et al., 2016; Jin et al., 2018). Because of the complexity of translation, some researchers have reported that the relative abundance of an mRNA and its corresponding protein may not show similar ratios among groups (Maier et al., 2009). However, the trends in the changes in mRNA and protein levels are similar in most cases such that high protein levels likely result from both frequent transcription and high mRNA stability (de Sousa Abreu et al., 2009; Maier et al., 2009). Therefore, we propose that a combination of qRT-PCR and immunoblotting or ELISAs would be a better choice for DEPs identification in future work.

## Conclusion

The DEPs identified in cells grown in 0, 10, and 20% NaCl via iTRAQ could be categorized into 20 GO functional groups and 24 KEGG pathways. These results indicate that extremely high osmotic stress upregulates the levels of proteins involved in fatty acid synthesis, those of the Clp protease family, and proteins involved proline/glycine betaine transportation and energy metabolism. In contrast, high osmotic stress downregulates the levels of proteins involved in biofilm formation and pathogenic infection. This study clarifies the mechanism of salt tolerance in *S. aureus* and provides information that is useful for the prevention and control of *S. aureus* contamination during high-salt aquatic production and preservation.

## Author Contributions

CL, JZ, and XS conceived and designed the experiments. TM, LG, YF, SH, DZ, and YYL carried out the experiments and data analysis. TM, LG, CL, YL, LC, and XS were involved in drafting the manuscript. All authors read and approved the final manuscript.

## Conflict of Interest Statement

DZ was employed by the company Zhejiang Zhengli Antuo Biotechnology Co., Ltd. The remaining authors declare that the research was conducted in the absence of any commercial or financial relationships that could be construed as a potential conflict of interest.

## Footnotes

1 http://www.uniprot.org/

2 http://www.genome.jp/kegg/tool/map_pathway2.html
