# Supplementary material for: iTRAQ-Based Quantitative Proteomic Profiling of Staphylococcus aureus Under Different Osmotic Stress Conditions
Source: Front Microbiol. 2019 May 29;10:1082. doi: 10.3389/fmicb.2019.01082 (PMC6549500; doi:10.3389/fmicb.2019.01082)
Supplement: Supplementary file 1 [file Data_Sheet_1.PDF]

Supplementary Material

iTRAQ-based quantitative proteomic profiling of *Staphylococcus aureus* under different osmotic stress conditions

Tinghong Ming, Lingxin Geng, Ying Feng, Chenyang Lu, Jun Zhou, Yanyan Li, Dijun Zhang, Shan He, Ye Li, Lingzhi Cheong, Xiurong Su*

*** Correspondence:** Xiurong Su: suxiurong_public@163.com

Supplementary materials legends

Table S1 Primers used in this study.

Table S2 The cell wall thickness of cells grown under the different concentrations of NaCl.

Table S3 The detailed information including information of protein quantification date, and average iTRAQ ratio for these identified proteins. The DEPs with fold change ⩾2 or ⩽0.5 in at least one comparison group are labelled in red.

Table S4 Differentially expressed proteins in the 10% NaCl group compared with the control group. The DEPs with fold change ⩾2 or ⩽0.5 are labelled in red.

Table S5 Differentially expressed proteins in the 20% NaCl group compared with the control group. The DEPs with fold change ⩾2 or ⩽0.5 are labelled in red.

Table S6 Differentially expressed proteins in the 20% NaCl group compared with the 10% NaCl group. The DEPs with fold change ⩾2 or ⩽0.5 are labelled in red.

Table S7 The upregulated proteins in both the 10% NaCl group compared with the control group and in the 20% NaCl group compared with the 10% NaCl group.

Table S8 The upregulated proteins in the 10% NaCl group compared with the control group, and the downregulated proteins in the 20% NaCl group compared with the 10% NaCl group.

Table S9 The downregulated proteins in the 10% NaCl group compared with the control group, and the upregulated proteins in the 20% NaCl group compared with the 10% NaCl group.

Table S10 The downregulated proteins in both the 10% NaCl group compared with the control group and in the 20% NaCl group compared with the 10% NaCl group.

Figure S1 After incubation for 48 h, the cell growth of *S. aureus* under the different concentrations of NaCl. (A) Control group; (B) 10% NaCl group; (C) 20% NaCl group.
