# Supplementary material for: iTRAQ-Based Quantitative Proteomic Profiling of Staphylococcus aureus Under Different Osmotic Stress Conditions
Source: Front Microbiol. 2019 May 29;10:1082. doi: 10.3389/fmicb.2019.01082 (PMC6549500; doi:10.3389/fmicb.2019.01082)
Supplement: Supplementary file 2 [file Data_Sheet_2.PDF]

Table S1 Primers used in this study.

Primer	Sequence (5′ – 3′)
betA-F	AGTTAAAACGAGGGCCAGCA
betA-R	CTTGAAGCTGACATTCGGCG
glnA -F	CATGGACTGCTGGACAAGGT
glnA -R	GCACGAGGATCCCCTTCAAA
mutS2-F	TCCAAGTGAAGGTGCTGCAT
mutS2-R	CCGGCACACCCATTAACAAC
nrdR-F	TGGCACAAGAGAGCAATTTTCA
nrdR-R	TGGACGCTTTTCACATGAACG
opuD_1-F	CCTGACCATGCTGACCAAGT
opuD_1-R	ATGCGTGGACCTTTAGGTGG
orfX-F	ATCTTCCGAAGGATTGGCCC
orfX-R	CCGTTTGATCCGCCAATGAC
psaA-F	AATCTGTGTACACTTCACCGAAGA
psaA-R	GCAATCGAGTTTGTAAAAGCACATA
recR-F	CGCCTATGGATGGCATTGGA
recR-R	TCCCCCTCTAAGTTCGGGTT
sat-F	GCACGCGCGATATCTCAAAT
sat-R	CCTAAAACTTTCGCACCCGC
sdrD-F	GCGACTCAGACTCAGACAGT
sdrD-R	TACCTTCACTACCTGTTTCTGG
sec2-F	CCTGCACCAGGCGATAAGTT
sec2-R	GAATCAACCGTTTTATTGTCGTTGT
ureB-F	CTGGAGCAGCTGTTCGATTTG
ureB-R	TCAGTTGGGCGATAGACACG
icaA-F	CCGCTGCCTTAGTAGTTGCT
icaA-R	GATTGCAGAAACACCCGACG
srrB-F	TCGCTTGCCATTGTCCTTGA
srrB-R	ACGCTGCAATAGGCTGAACT
sigA-F	CATGGCAAACGCGTGATGAT
sigA-R	TACACCTTGTGCCTGCCATT
icaB-F	GCAGTCACTCCGAACTCCAA
icaB-R	TTCATGGAATCCGTCCCATCTC

**Primer**	**Sequence (5′ – 3′)**
clpP-F	AACAACAAACCGCGGTGAAC
clpP-R	TTCTCTGAGTCTTGCGCTTGT
luxs-F	TCCTATGGGTTGCCAAACTGG
luxs-R	TGCCCAGCCACATTGTACTT
clfA-F	AAATCGATTGGCGTGGCTTC
clfA-R	GATTTTGCGCCACACTCGTT
sbi-F	AGCGAGTGAAAACACGCAAC
sbi-R	CACGTCGGTCTGGGTTCTTG
saeR-F	GGCTCCAAAGAAACTAGCAGC
saeR-R	TGTTGTCCCACTCGGAGAGA
lrgB-F	CAAGTGCTAATCCTCGGGCA
lrgB-R	TCAAGCAGCAACTACAGCGA
lrgA-F	TGCAGGCATAGGAATTGGCA
lrgA-R	ACGCATCAAAACCAGCACAC
spa-F	ACGGCACTACTGCTGACAAA
spa-R	GCATGGTTTGCTGGTTGCTT
