# Supplementary material for: iTRAQ-Based Quantitative Proteomic Profiling of Staphylococcus aureus Under Different Osmotic Stress Conditions
Source: Front Microbiol. 2019 May 29;10:1082. doi: 10.3389/fmicb.2019.01082 (PMC6549500; doi:10.3389/fmicb.2019.01082)
Supplement: Supplementary file 3 [file Data_Sheet_3.PDF]

Table S2 The cell wall thickness of cells grown under the different concentrations of NaCl. "***" represented $p < 0.01$.

Group	No. of cells measured	Mean avg cell wall thickness (nm) ± SD
0% NaCl group	25	19.89±4.82
10% NaCl group	25	33.34±3.17 ***
20% NaCl group	25	44.48±4.46 ***
