# Supplementary material for: iTRAQ-Based Quantitative Proteomic Profiling of Staphylococcus aureus Under Different Osmotic Stress Conditions
Source: Front Microbiol. 2019 May 29;10:1082. doi: 10.3389/fmicb.2019.01082 (PMC6549500; doi:10.3389/fmicb.2019.01082)
Supplement: Supplementary file 4 [file Data_Sheet_4.PDF]

Table S4 Differentially expressed proteins in the 10% NaCl group compared with the control group. The DEPs with fold change ≥2 or ≤0.5 are labelled in red.

Accession Number	Protein Name	Gene Name	Fold Change
A0A0D1IXJ7	Gamma-hemolysin B subunit HlgB	*SAJPND1_02412*	0.08
A0A0D6DK32	Extracellular matrix binding protein	*SAJPND1_00798*	0.09
A0A0D6DCF3	Extracellular fibrinogen-binding protein	*SAJPND1_01081*	0.09
A0A0D6DFZ4	Leukocidin S subunit LukS	*SAJPND1_01995*	0.10
A0A090LXD5	Uncharacterized protein	-	0.10
A0A0D6DGW3	Gamma-hemolysin A subunit HlgA	*SAJPND1_02409*	0.11
OMP7	77 kDa membrane protein	*SACOL2002*	0.12
A0A0D1K3U3	Extracellular fibrinogen-binding protein	*QU38_12095*	0.13
A0A0D1JXR9	Strain SA-120 Contig630, whole genome shotgun sequence	*QU38_15890*	0.15
Q9AFB0	Leukocidin LukS component (Fragment)	-	0.19
SBI	Immunoglobulin-binding protein sbi	*sbi*	0.22
A0A0D6DFD4	Beta-lactamase regulator protein	*SAJPND1_01896*	0.22
A0A0C5HVE0	Pyruvate decarboxylase%3B Alpha-keto-acid decarboxylase	*ipdC*	0.22
A0A077TWE9	Lipoprotein	*ERS094548_00195*	0.22
A0A0C5HSI2	Gamma-hemolysin component B	*ERS445051_01979*	0.23
A0A0D6HPJ5	Lantibiotic transport ATP-binding protein	*ecsA_3*	0.23
A0A0D1H8B0	Threonine-tRNA ligase	*A0A0D1H8B0*	0.25
A7E1C4	Enterotoxin C2 (Fragment)	*sec2*	0.25
A0A068A489	Inhibitor	*scn_2*	0.25
A0A0D1I054	L-lactate dehydrogenase	*ldh*	0.25
A0A090LSD7	Uncharacterized protein	-	0.26
A0A077U8P9	Membrane spanning protein	*ERS140026_02521*	0.27
A0A090N157	Alpha-hemolysin	*hly*	0.28
ADH	Alcohol dehydrogenase	*adh*	0.29
A0A0D6DIM1	Ribokinase	*SAJPND1_00306*	0.29
A0A0D1HN80	Strain SA-120 Contig620, whole genome shotgun sequence	-	0.29
A0A077U1D0	Amino acid permease	*steT*	0.30
A0A0B6XPE8	Pyruvate carboxylase	*cfiB_1*	0.30
A0A0E0VS19	Carboxylic ester hydrolase	*ST398NM01_2504*	0.30
A0A077VAK0	Hexose phosphate transport protein	*uhpT*	0.30
A0A090LUS7	AraC family transcription regulator	*araC*	0.30
A0A077TT97	DNA-damage repair protein	*umuC*	0.30
A0A0D6DGG6	Uncharacterized protein	-	0.31
A0A0B6XL12	Pseudouridine-5'-phosphate glycosidase	*psuG*	0.32
A0A077U927	Pseudouridine-5'-phosphate glycosidase	*psuG*	0.32
A0A077V7K3	ABC transporter substrate-binding protein	*psaA*	0.33
A0A090LUS6	Lipoprotein	*yehR*	0.33
A0A0C2HZY8	Pyrimidine-nucleoside phosphorylase	*pdp*	0.33
A0A0D1GJW4	Arsenate reductase family protein	*QU38_11110*	0.33

Accession Number	Protein Name	Gene Name	Fold Change
A0A0D6FNQ5	Immunoglobulin G-binding protein A	*spa*	0.33
A0A0D1HEF4	6,7-dimethyl-8-ribityllumazine synthase	*ribH*	0.33
A0A0D1JQX0	Strain SA-120 Contig628, whole genome shotgun sequence	-	0.35
Q9S2Z4	Cell surface protein map-w	*map-w*	0.35
Q7DK27	ABC transporter ATP-binding protein	*stpA*	0.35
A0A0D6DN91	Thymidine phosphorylase	*SAJPND1_02113*	0.35
A0A077VMN9	Peptide methionine sulfoxide reductase MsrA	*msrA_1*	0.35
A0A090LW57	ABC transporter, permease protein	*SAU060112_100003*	0.36
A0A090LWP1	HTH-type transcriptional regulator SarS	*sarS*	0.38
A0A0D1GD88	2,3-bisphosphoglycerate-dependent phosphoglycerate mutase	*gpmA*	0.39
A0A0B6XTB4	N-acetylmuramoyl-L-alanine amidase%2C family 4	*ERS094548_01923*	0.39
A0A0D1G5I0	Strain SA-120 Contig630, whole genome shotgun sequence	*QU38_11030*	0.40
A0A0D1HCV5	Uncharacterized protein	-	0.40
A0A0D6GIM9	Autolysin	*atl_1*	0.40
A0A0C5HYQ5	SepS16B protein	*sepS16B*	0.40
A0A077UDD9	3-ketoacyl-CoA thiolase%3B Acetyl-CoA acetyltransferase	*ERS140248_02364*	0.40
A0A077VSV4	Peptide ABC transporter permease	*oppB_1*	0.40
A0A0D3QBH3	Peptide synthetase	*grsB*	0.40
A0A0A0Q4N9	HtrA-like serine protease	*htrA*	0.41
A0A0D1IIH6	Uncharacterized protein	-	0.42
A0A0D1GTD3	Strain SA-120 Contig622, whole genome shotgun sequence	*QU38_05245*	0.42
A0A0D6DDK7	Uncharacterized protein	*SAJPND1_01250*	0.42
A0A069GEF6	Threonine--tRNA ligase	*thrS_1*	0.42
A0A0D1HUP1	Nucleoside transporter	*QU38_09615*	0.43
A0A0E1AGE4	Uncharacterized protein	-	0.44
A0A090M2J3	Uncharacterized protein	*SAU060112_70120*	0.44
A0A0E1AHK4	Foldase protein PrsA	*prsA*	0.44
A0A0D1HQ98	Histidine kinase	*QU38_10565*	0.45
A0A090LTY1	Uncharacterized protein	-	0.45
A0A0E1AMS3	Uncharacterized protein	-	0.45
A0A0E1VQA6	Immunoglobulin-binding protein sbi	-	0.45
A0A0D1HD00	Universal stress protein	*uspA*	0.45
A0A090LR99	CobW/HypB/UreG, nucleotide-binding domain protein	*SAU060112_10579*	0.45
A0A077U988	Pyrimidine nucleoside transport protein	*nupC_2*	0.45
A0A0D1JLL3	Adenosylmethionine-8-amino-7-oxononanoate aminotransferase	*bioA*	0.45
A0A0B6XN73	3-oxoadipate enol-lactonase	*dehH1*	0.45
A0A0E1AE25	Phosphoglycolate phosphatase	*SAZ172_0574*	0.45
A0A0D1H739	Strain SA-120 Contig625, whole genome shotgun sequence	-	0.46
A0A0D1IB15	Strain SA-120 Contig624, whole genome shotgun sequence	-	0.46
A0A077UJ82	Uncharacterized protein	-	0.46
A0A0D1I600	Uncharacterized N-acetyltransferase QU38_12215	-	0.46

Accession Number	Protein Name	Gene Name	Fold Change
A0A0D1K2N9	GlnQ protein	*glnQ*	0.47
A0A090LX90	Probable transcriptional regulatory protein yeeN	*yeeN*	0.47
A0A0D1IJC4	Strain SA-120 Contig621, whole genome shotgun sequence	-	0.47
A0A0B6XP33	Oligopeptide transport ATP-binding protein oppD	*oppD_1*	0.47
A0A0D1IXM8	Small heat shock protein	*QU38_07680*	0.48
A0A077TYE1	Uncharacterized protein	-	0.49
A0A0B6XRG8	Lipoprotein	*ERS094548_00892*	0.49
A0A077W0Q5	UPF0365 protein ERS140167_00916	-	0.49
A0A090N246	Formate/nitrite transporter	*nirC*	0.50
A0A068W8S1	Ribosomal RNA large subunit methyltransferase H	*orfX*	0.50
A0A0D1GJ10	Strain SA-120 Contig630, whole genome shotgun sequence	*QU38_15345*	0.50
A0A0D1JPH9	Strain SA-120 Contig629, whole genome shotgun sequence	*QU38_12985*	0.50
A0A090LVY0	Aspartate carbamoyltransferase	*pyrB*	0.50
A0A0B6XKT1	Aldehyde-alcohol dehydrogenase	*adhE*	0.50
A0A077V9M9	Response regulator SaeR	*saeR*	0.50
A0A0D1JT33	Cardiolipin synthase	*QU38_06435*	0.50
A0A077TYE7	Uncharacterized protein conserved in bacteria	*ERS140095_00284*	0.50
A0A090M2D6	Oligoendopeptidase F	*SAU060112_70075*	0.50
A0A0D6DGT6	Cystine-binding protein	*SAJPND1_02402*	0.50
A0A0D1HBY5	Pyridoxal 5'-phosphate synthase subunit PdxT	*pdxT*	0.50
A0A0D1G640	Arginine repressor	*argR*	0.50
A0A090LY09	GTPase Obg	*obg*	0.50
A0A077UJ58	Pyridoxal 5'-phosphate synthase subunit PdxS	*pdxS*	0.51
A0A098H232	Chromosome replication initiation protein dnaD	*dnaD*	0.51
A0A090LXQ9	Uncharacterized protein	-	0.51
A0A0B6XNE5	Hydrolase (HAD superfamily)	*ERS094548_00694*	0.52
A0A0D6FS99	Putative polyribitolphosphotransferase	*tagF_1*	0.52
A0A090LZA4	Chorismate mutase I/2-keto-3-deoxy-D-arabino-heptulosonate-7-phosphate synthase I beta%2C AroH/AroA I beta	*aroA*	0.52
A0A0D1GN53	Strain SA-120 Contig628, whole genome shotgun sequence	-	0.53
A0A090LXT5	Peptidase C45	*SAU060112_110028*	0.53
A0A090LZI4	Uncharacterized protein	-	0.53
X5IX38	DNA-binding protein	*SAU060112_10479*	0.54
A0A0D1H4D9	Strain SA-120 Contig627, whole genome shotgun sequence	-	0.54
A0A077UDM1	Manganese ABC transporter%2C ATP-binding protein SitB	*sitB*	0.54
A0A0B6XP73	Lipoate-protein ligase	*lplJ_2*	0.54
A0A077U4E2	2-oxoglutarate/malate translocator-like protein	*ttdT*	0.54
A0A090M283	Catalase	*katA*	0.54
A0A077UZP7	Transcriptional repressor NrdR	*nrdR*	0.55
A0A0D1FF77	Coenzyme A disulfide reductase	*cdr*	0.55
A0A0D1HKP9	Arginase	*SAJPND1_02140*	0.55

Accession Number	Protein Name	Gene Name	Fold Change
A0A090N1I7	Putative aldehyde dehydrogenase YwdH	*ywdH*	0.55
A0A090LRF4	Uncharacterized protein	-	0.55
A0A090LYD3	Uncharacterized protein	-	0.56
A0A0D1HIC5	L-threonine 3-dehydrogenase	*QU38_09780*	0.56
A0A077UYW0	Uncharacterized protein	-	0.56
A0A0B6XQC4	Serine hydroxymethyltransferase	*glyA*	0.56
A0A090N1L4	Diacetyl reductase ((S)-acetoin forming)	*butA*	0.56
A0A0D1H5M4	Transaldolase	*QU38_04770*	0.56
A0A0D1JGI6	2-C-methyl-D-erythritol 4-phosphate cytidylyltransferase	*ispD*	0.56
A0A0B6XMG3	Argininosuccinate synthase	*argG*	0.57
A0A0D1GBS9	Signal transduction protein TRAP	*SAJPND1_01796*	0.57
A0A0E0VRM5	TenA	*ST398NM01_2137*	0.57
A0A0D1G1J9	Cytochrome c oxidase polypeptide II	*SAJPND1_00987*	0.58
A0A0D6DAQ1	6-phospho-beta-glucosidase	*SAJPND1_00245*	0.58
A0A077UG02	Acid phosphatase	*hel*	0.58
A0A077TZL7	Modification methylase MboII	*mboIIM*	0.58
A0A077VJX6	Deblocking aminopeptidase	*ysdC_1*	0.58
A0A090N1X3	Chaperone protein DnaK	*dnaK*	0.58
A0A090LW12	Poly (Glycerol-phosphate) alpha-glucosyltransferase	*SAU060112_10705*	0.58
A0A090LXY5	Uncharacterized protein	*SAU060112_120007*	0.58
A0A0D1GVJ7	Poly (Glycerol-phosphate) alpha-glucosyltransferase	*SAJPND1_00558*	0.59
A0A0D6DJ43	Teichoic acid biosynthesis protein X	*SAJPND1_00639*	0.59
A0A090LR26	Uncharacterized oxidoreductase YcsN	*ycsN*	0.60
A0A080V4N7	Membrane spanning protein	*yhhT_1*	0.60
A0A0E1VKR4	ABC transporter, ATP-binding protein	*HMPREF0776_1978*	0.60
A0A0D1IUP6	Strain SA-120 Contig628, whole genome shotgun sequence	-	0.60
A0A090LWI3	Argininosuccinate lyase	*argH*	0.60
A0A077TYC4	Similar to putative sodium/glucose cotransporter	*sglT*	0.60
A0A090M057	Putative ATP-dependent helicase DinG homolog	*dinG*	0.60
A0A0B6XKT2	Ribose operon repressor%2C putative	*degA*	0.60
A0A0D1I5J3	Ribulose-phosphate 3-epimerase	*QU38_12445*	0.60
A0A069FTI7	Hydroxymethylpyrimidine kinase/phosphomethylpyrimidine kinase	*thiD_1*	0.60
A0A090LUN2	Na+/H+ antiporter family protein	*SAU060112_40163*	0.60
A0A0D1IL35	NAD kinase	*ppnK*	0.60
A0A0D1FA95	Negative regulator of genetic competence clpC	*QU38_09635*	0.60
A0A0B4NAS5	30S ribosomal protein S1	*rpsA*	0.60
A0A077W554	Membrane associated protein	*ERS140026_02425*	0.60
A0A090M1C4	Dihydroorotate dehydrogenase (quinone)	*pyrD*	0.61
A0A0B6XL00	Lipoprotein	*metQ_1*	0.61
A0A0D6GYJ2	Alanine dehydrogenase	*ald1*	0.61
A0A0C5HTL6	Dihydroxyacetone kinase	*dhaL*	0.63

Accession Number	Protein Name	Gene Name	Fold Change
A0A0E0VSZ0	GTP pyrophosphokinase	*ST398NM01_2559*	0.63
A0A069GA97	30S ribosomal protein S1	-	0.63
A0A077U7R1	Cytochrome d ubiquinol oxidase subunit I	*cydA*	0.63
A0A0B6XNT6	Bifunctional ligase/repressor BirA	*birA*	0.64
A0A0E1VLF4	TIGR01777 family protein	*HMPREF0776_1780*	0.64
A0A090LUN7	Urocanate hydratase	*hutU*	0.64
A0A0D1J363	Uracil phosphoribosyltransferase	*upp*	0.64
A0A0D6DNP1	Uncharacterized protein	-	0.65
A0A090LUL7	DeoR-like helix-turn-helix domain protein	*SAU060112_40148*	0.67
A0A0E1VIJ7	HTH-type transcriptional regulator rot	*HMPREF0776_2797*	0.67
A0A0D6GAR1	Carboxylesterase	*est_2*	0.67
A0A0D6DK53	Glyoxylatereductase	*SAJPND1_00846*	0.67
A0A0B6XRM8	Glycine betaine transport ATP-binding protein	*proV_2*	0.67
A0A0D1HL09	Redox-sensing transcriptional repressor Rex	*rex*	0.67
A0A077U2Q8	Quinol oxidase polypeptide I QoxB	*qoxB*	0.67
A0A0D1GC10	Strain SA-120 Contig629, whole genome shotgun sequence	-	0.67
A0A0C5HTQ6	Oxidoreductase of aldo/keto reductase family%2C subgroup 1	*yvgN_1*	0.68
A0A0D1GID9	Strain SA-120 Contig630, whole genome shotgun sequence	-	0.68
A0A090N216	Uncharacterized protein	-	0.68
A0A0D1JYJ4	Strain SA-120 Contig628, whole genome shotgun sequence	*QU38_10035*	0.68
A0A077UFT7	Lipoprotein	*ERS140147_00473*	0.68
A0A0D6H2B5	Salicylate hydroxylase	*nagX*	0.68
A0A0D1HD94	Serine acetyltransferase	*sat*	0.69
A0A0B6XQW2	2-oxoisovalerate dehydrogenase subunit alpha	*bfmBAA*	0.69
A0A0E8GI61	GTPase Der	*engA*	0.69
A0A0D1K8W2	Bifunctional protein PyrR	*pyrR*	0.70
A0A0C5HMT4	GntR family transcriptional regulator	*treR_1*	0.70
A0A0D1H6Z1	Nitric oxide synthase oxygenase	*QU38_06960*	0.70
A0A0E1AE46	Phosphoenolpyruvate-dihydroxyacetone phosphotransferase, dihydroxyacetone binding subunit DhaK	*SAZ172_0661*	0.70
A0A077UP25	Membrane-bound serine protease	*ERS140159_00273*	0.70
A0A069FW70	Fructose-1,6-bisphosphatase class 3	*fbp*	0.70
A0A0B6XKQ5	Deoxyribose-phosphate aldolase	*dra_1*	0.70
A0A0D6DGV1	Staphyloxanthin biosynthesis protein CrtQ	*SAJPND1_02565*	0.70
A0A077U1Q7	Oxidoreductase%2C aldo/keto reductase family	*ERS140248_01341*	0.70
A0A0D1JZ91	General stress protein	*QU38_08215*	0.70
A0A090N275	Transcriptional regulator, TetR family	*SAU060112_40385*	0.70
A0A090M109	Gluconate operon transcriptional repressor	*gntR*	0.70
A0A090LYF9	Acetoin dehydrogenase E1 component beta-subunit	*acoB*	0.70
A0A0B6XKM4	Histidine ammonia-lyase	*hutH*	0.70
A0A0D6DEP2	Beta-lactamase	*SAJPND1_01897*	0.70
A0A0D1IUZ1	Citrate synthase	*QU38_08465*	0.70

Accession Number	Protein Name	Gene Name	Fold Change
A0A077U7M7	Two component transcriptional regulator VraR%2C LuxR family	*vraR_2*	0.70
A0A0B6XL04	PTS system trehalose-specific transporter subunit IIBC	*treB*	0.71
A0A0B6XRJ3	Riboflavin biosynthesis protein RibD	*ribD*	0.71
A0A0D1JNX0	Glutamate racemase	*murI*	0.71
A0A0B6XRK6	Nicotinate phosphoribosyltransferase	*ERS094548_02369*	0.71
A0A0D1EZS5	Proline dehydrogenase	*SAJPND1_01717*	0.71
A0A0D1FPR0	3-hydroxyacyl-[acyl-carrier-protein] dehydratase FabZ	*fabZ*	0.72
A0A090LVA4	Sulfite reductase [NADPH] flavoprotein alpha-component	*cysJ*	0.72
A0A0D6DMA3	2-succinylbenzoate-CoA ligase	*menE*	0.72
A0A0D6DPK7	Staphyloxanthin biosynthesis protein CrtP	*SAJPND1_02566*	0.73
A0A090LYL9	Teichoic acid biosynthesis protein F	*SAU060112_20185*	0.73
A0A0B6XL97	Periplasmic binding protein	*btuF*	0.73
A0A0B6XPW9	Sodium/proline symporter	*putP*	0.73
A0A0B6XQ10	Exported protein	*ERS094548_02603*	0.73
A0A077UAU6	Transcription regulator (Contains diacylglycerol kinase catalytic domain)	*dagK*	0.73
A0A0B6XQX6	Bifunctional protein: zinc-containing alcohol dehydrogenase%3B quinone oxidoreductase (NADPH:quinone reductase)	*ERS094548_01069*	0.73
A0A0E1X794	DEAD/DEAH box helicase	*HMPREF0769_12121*	0.73
A0A077UUV1	Hydrogen peroxide-inducible genes activator	*oxyR*	0.73
A0A0A0R7J1	RepL	-	0.73
A0A0D1I4D2	Oligoendopeptidase F	*QU38_11615*	0.74
A0A077UNV6	Pseudouridine synthase	*rluB*	0.74
A0A090LUF4	Ribose-5-phosphate isomerase A	*rpiA*	0.74
A0A090LUE4	Pseudouridine synthase	*ylyB*	0.75
A0A0D1HRD3	Strain SA-120 Contig627, whole genome shotgun sequence	-	0.75
A0A0D1JST6	Peroxide operon regulator	*QU38_07270*	0.75
A0A0D1GUW7	GTPase Era	*era*	0.75
A0A0D1IPW1	DNA polymerase III subunit beta	*QU38_15525*	0.75
A0A0B6XMZ2	Succinate dehydrogenase flavoprotein subunit	*sdhA_1*	0.75
A0A090LZZ7	Uncharacterized peptidase YqhT	*YqhT*	0.76
A0A077UYI3	Ribonuclease 3	*rnc*	0.76
A0A077UJ41	3-methyl-2-oxobutanoate hydroxymethyltransferase	*panB*	0.76
A0A090LVR9	Uncharacterized protein	*SAU060112_10595*	0.76
A0A077U7P6	Sugar phosphate isomerase/epimerase	*ERS140159_00782*	0.76
A0A0D1IP31	Septation ring formation regulator EzrA	*ezrA*	0.76
A0A0D1IBM2	DEAD-box ATP-dependent RNA helicase CshA	*cshA*	0.77
A0A0D6H5T9	NAD-dependent protein deacetylase	*cobB*	0.77
A0A0D6DD44	Glycerophosphoryl diester phosphodiesterase	*SAJPND1_01047*	0.77
A0A0D1IBW0	Strain SA-120 Contig620, whole genome shotgun sequence	*QU38_04480*	0.77

Accession Number	Protein Name	Gene Name	Fold Change
A0A0D1G9T6	Tautomerase	*QU38_13190*	0.77
A0A0D1IKK8	10 kDa chaperonin	*groS*	0.77
A0A0D1JZP5	DNA repair protein RecN	*QU38_09335*	0.78
A0A0E1VKG9	Endonuclease MutS2	*mutS2*	0.78
A0A0D1HJ56	Strain SA-120 Contig628, whole genome shotgun sequence	-	0.78
A0A090LXX3	DNA polymerase	*polA*	0.78
A0A0D6DLY3	Pyrroline-5-carboxylate reductase	*proC*	0.78
A0A0D6DJB9	Histidinol-phosphate aminotransferase	*hisC*	0.78
A0A0D1HJP2	Ribosomal large subunit pseudouridine synthase D	*SAJPND1_01814*	0.78
A0A0D1HWP8	Dephospho-CoA kinase	*coaE*	0.78
A0A0D6DM29	5-formyltetrahydrofolate cyclo-ligase	*SAJPND1_01504*	0.78
A0A0D1JR64	Accessory regulator a	*QU38_10110*	0.78
A0A068A8Z0	Protein GrpE	*grpE*	0.78
A0A0D1HZ15	Cell division protein divic	*QU38_03180*	0.78
A0A0D1FJV3	Glucose-6-phosphate 1-dehydrogenase	*zwf*	0.79
A0A090LXP9	Uroporphyrinogen decarboxylase	*hemE*	0.79
A0A0D3Q7P5	3-phosphoshikimate 1-carboxyvinyltransferase	*aroA_2*	0.79
A0A090LXB7	Luciferase-like monooxygenase	*SAU060112_20273*	0.79
A0A0D6DFF7	Uncharacterized protein	-	0.80
A0A090LVS1	Potassium-transporting ATPase B chain	*kdpB*	0.80
A0A0D6HWL4	ATP-dependent helicase/deoxyribonuclease subunit B	*addB*	0.80
A0A0E1VI43	Carbamoyl-phosphate synthase (glutamine-hydrolyzing)	*carB*	0.80
A0A090N1M7	Type-1 restriction enzyme R protein	*hsdR*	0.80
CCA	CCA-adding enzyme	*cca*	0.80
A0A0D1J100	Ribosomal-protein-alanine acetyltransferase	*SAJPND1_02696*	0.80
A0A0B6XMQ8	Myo-inositol-1(Or 4)-monophosphatase	*suhB_1*	0.80
A0A0D6DHW6	Glycosyltransferase	*SAJPND1_00237*	0.80
A0A077UBB6	Type I restriction modification DNA specificity domain protein	*hsdS*	0.80
A0A0D1K8D2	Tyrosine recombinase XerD	*xerD*	0.80
A0A090LYW3	Molybdopterin molybdenumtransferase	*moeA*	0.80
A0A0D6GHE7	Protease	*ERS445051_00905*	0.80
A0A0E1AFV2	Non-canonical purine NTP pyrophosphatase	*SAZ172_1150*	0.80
A0A0D1JJX2	Mevalonate kinase	*QU38_09960*	0.80
A0A069G475	Pyridine nucleotide-disulfide oxidoreductase	*CO98_1154*	0.80
A0A090LQG8	Protein dltD	*SAU060112_10398*	0.80
A0A0C5I2R4	Peptidase propeptide and YPEB domain-containing protein	*ERS445051_01691*	0.80
A0A0D1JKT6	S-adenosylmethionine:tRNA ribosyltransferase-isomerase	*queA*	0.80
A0A0D1HD79	tRNA binding domain protein	*SAJPND1_01690*	0.80
A0A0D1I2B3	Chaperone protein DnaJ	*dnaJ*	0.80
A0A0D1HV16	Putative cytosolic protein	*QU38_12695*	0.81
A0A0D6DEN2	Uncharacterized protein	-	0.81

Accession Number	Protein Name	Gene Name	Fold Change
A0A090LYF0	Acetyl-CoA carboxylase, biotin carboxylase subunit	*accC*	0.82
A0A0D1I4Q5	Strain SA-120 Contig630, whole genome shotgun sequence	-	0.82
A0A0D1G9L3	Adenylosuccinate lyase	*QU38_06995*	0.82
A0A077U409	Protein GrpE	*grpE*	0.82
A0A0D6GTK5	3-hydroxy-3-methylglutaryl coenzyme A reductase	*mvaA*	0.83
A0A0B6XM19	Signal peptidase I	*spsB*	0.83
A0A077ULT9	Ribonuclease R	*rnr*	0.83
A0A0D1JS50	Strain SA-120 Contig626, whole genome shotgun sequence	*QU38_07375*	0.83
A0A0E1XJY5	Abi-like protein	*HMPREF0769_10093*	0.83
A0A033ULT9	ATP synthase subunit c	*V070_02071*	1.20
A0A0B6XRB8	Sodium export permease protein	*yhaP*	1.20
A0A0C5HFW6	Aromatic amino acid aminotransferase	*patA*	1.20
A0A0B6XM00	Iron binding protein from the HesB_IscA_SufA family	*ERS094548_00056*	1.20
A0A090LQ01	Putative DNA-binding protein	*SAU060112_10164*	1.20
A0A077W129	30S ribosomal protein S13	*rpsM*	1.20
A0A069FUU4	30S ribosomal protein S11	*rpsK*	1.20
A5X5X2	RNA polymerase-beta subunit (Fragment)	*rpoB*	1.20
A0A0D1FKQ9	30S ribosomal protein S9	*rpsI*	1.20
A0A090LTG7	Acetyl-coenzyme A carboxylase carboxyl transferase subunit beta	*accD*	1.21
A0A0D1H0K7	50S ribosomal protein L23	*rplW*	1.21
A0A0C5HKE6	Hypothetical esterase/lipase	*pip*	1.21
A0A0B6XLD2	Sporulation protein	*yaaT*	1.21
A0A0E1AM34	Uncharacterized protein	-	1.21
A0A077U0Z6	Biotin carboxyl carrier protein of acetyl-CoA carboxylase	*accB*	1.22
A0A0D1JM30	Ribosomal silencing factor RsfS	*rsfS*	1.22
A0A0B6XLK3	Haloacid dehalogenase-like hydrolase	*ppaX*	1.22
A0A0D1I5G9	50S ribosomal protein L19	-	1.22
A0A090LVT9	DNA topoisomerase 1	*topA*	1.22
A0A0D1G616	Putative cytosolic protein	*QU38_09200*	1.23
A0A0D1H8Z3	3-oxoacyl-[acyl-carrier-protein] synthase 3	*fabH*	1.23
A0A0D1GMH3	50S ribosomal protein L17	-	1.23
A0A0D1K0H1	30S ribosomal protein S16	-	1.24
A0A0D1HQY5	Nucleoside diphosphate kinase	*ndk*	1.24
A0A0B6XQS8	Valine--tRNA ligase	*valS*	1.24
A0A0D1HT39	NADH dehydrogenase	*QU38_11305*	1.24
A0A0D1I3J5	Urease accessory protein UreE	*ureE*	1.25
A0A0E0VNZ9	Glyceraldehyde-3-phosphate dehydrogenase	*ST398NM01_0851*	1.25
A0A0D1HT84	50S ribosomal protein L5	-	1.25
A0A0E0VM51	Acetate CoA-transferase YdiF	*ST398NM01_0245*	1.25
A0A090LTN7	Adenine phosphoribosyltransferase	*apt*	1.25

Accession Number	Protein Name	Gene Name	Fold Change
A0A090N220	Cyclic pyranopterin monophosphate synthase accessory protein	*moaC*	1.25
A0A0B6XL55	Hydrolase (HAD superfamily)	*ywpJ_1*	1.25
A0A0D6HKP6	Asparaginase	*ansA*	1.25
A0A0D1HYX5	50S ribosomal protein L14	*rplN*	1.25
A0A0E1XGI2	Uncharacterized protein	-	1.26
A0A090LQH4	Protein NagD homolog	*nagD*	1.27
A0A0D1HVU3	Strain SA-120 Contig623, whole genome shotgun sequence	-	1.28
A0A090N2E5	Mannitol-specific phosphotransferase enzyme IIA component	*mtlF*	1.28
A0A0E1AJ22	Probable thiol peroxidase	*tpx*	1.28
A0A0D1GWI4	50S ribosomal protein L4	*rplD*	1.28
A0A0D6DCH6	Scaffold protein sufB	*SAJPND1_00832*	1.29
A0A0D1IW07	Histidine-tRNA ligase	*hisS*	1.29
A0A0D6HFE5	Repressor-like protein	*ERS445051_01972*	1.30
A0A0D1JPL9	Strain SA-120 Contig616, whole genome shotgun sequence	-	1.30
A0A0D1HF64	30S ribosomal protein S18	-	1.30
A0A0E1AHK5	Acetylornithine deacetylase/Succinyl-diaminopimelate desuccinylase-related deacylase	*SAZ172_1765*	1.30
A0A0B6XLN0	Uracil-DNA glycosylase	*ung*	1.30
A0A0E8GH95	Phage protein	*ERS195391_00924*	1.31
A0A0C2HDA0	Glycerol-3-phosphate dehydrogenase	*glpD*	1.33
A0A090LRD1	Molecular chaperone Hsp31 and glyoxalase 3	*hchA*	1.33
A0A0D1I6P2	Diaminopimelate decarboxylase	*lysA*	1.33
A0A0B6XNR9	Probable glycine dehydrogenase (decarboxylating) subunit 2	*gcvPB*	1.33
A0A0D1GNJ9	Putative pit accessory protein	*QU38_10340*	1.33
A0A077V1V2	50S ribosomal protein L10	*rplJ*	1.33
A0A0D1HI29	Putative tRNA (cytidine(34)-2'-O)-methyltransferase	*QU38_03970*	1.33
A0A0D1IHK1	50S ribosomal protein L20	*rplT*	1.33
A0A0D1HT50	Adenylate kinase	*adk*	1.34
A0A077VUG4	Hypoxanthine-guanine phosphoribosyltransferase	*hpt*	1.35
A0A0B6XN60	Probable dual-specificity RNA methyltransferase RlmN	*rlmN*	1.35
A0A0D1H0B5	50S ribosomal protein L27	*rpmA*	1.36
A0A0B6XP80	Similar to oxidoreductase	*yhdN*	1.36
A0A0B6XPL1	Aldo/keto reductase family protein	*yvgN*	1.36
A0A0D1HQV9	30S ribosomal protein S6	*rpsF*	1.38
A0A0D1J193	30S ribosomal protein S12	-	1.38
A0A068DY65	DNA ligase	*lig*	1.39
A0A0C5HKH2	PTS-dependent dihydroxyacetone kinase phosphotransfer protein	*dhaM*	1.40
A0A0D1FBM5	6-phosphogluconolactonase	*SAJPND1_01893*	1.40
A0A077W1K8	Pyrrolidone-carboxylate peptidase	*pcp*	1.40
A0A0B6XSS6	Molybdate-binding protein	*modA*	1.40

Accession Number	Protein Name	Gene Name	Fold Change
A0A0D6DDB0	Dihydrofolate reductase	*SAJPND1_01369*	1.41
A0A0D1FXV8	Succinyl-CoA ligase [ADP-forming] subunit beta	*sucC*	1.42
A0A090LYX3	Inosine-uridine preferring nucleoside hydrolase	*SAU060112_40119*	1.42
A0A0D1J2E1	Translation initiation factor IF-3	*infC*	1.43
A0A0B6XPZ7	Dihydrolipoyllysine-residue succinyltransferase component of 2-oxoglutarate dehydrogenase complex	*odhB*	1.43
A0A0D6H0T7	Phage protein	*ERS445051_01427*	1.43
A0A033UV78	50S ribosomal protein L36	*rpmJ*	1.44
A0A090LXL4	Glutamate-tRNA ligase	*gltX*	1.45
A0A090LYB7	UDP-N-acetyl glucosamine-2-epimerase	*rffE*	1.45
A0A077U9X8	Two-component response regulator	*nreC*	1.46
A0A0D1I1V1	50S ribosomal protein L7/L12	-	1.47
A0A0D1HES6	Strain SA-120 Contig628, whole genome shotgun sequence	-	1.48
A0A0D1IKI9	Strain SA-120 Contig619, whole genome shotgun sequence	-	1.49
A0A0D1GP04	Succinyl-CoA ligase [ADP-forming] subunit alpha	*SAJPND1_01177*	1.49
A0A0D1HKI2	Ferric uptake regulation protein	*QU38_09445*	1.50
A0A077TYV7	3-oxoacyl-[acyl-carrier-protein] synthase 2	*fabF*	1.50
A0A0B6XNS5	Aspartate-semialdehyde dehydrogenase	*asd*	1.50
A0A0B6XQU1	Molybdenum cofactor biosynthesis protein E	*moaE*	1.50
A0A0D1HN41	Phosphoenolpyruvate carboxykinase [ATP]	*pckA*	1.51
A0A069FZ92	Branched-chain-amino-acid aminotransferase	*ilvE*	1.51
A0A0D1EZR2	Strain SA-120 Contig627, whole genome shotgun sequence	*QU38_08415*	1.51
A0A0D6DE20	Alanine dehydrogenase	*SAJPND1_01657*	1.52
A0A0D1I3V2	UDP-N-acetylenolpyruvoylglucosamine reductase	*murB*	1.53
A0A0D3Q4K0	ABC transporter permease protein	*CH51_01470*	1.53
A0A0D1HTP8	Strain SA-120 Contig628, whole genome shotgun sequence	-	1.54
A0A0D1H088	50S ribosomal protein L35	*rpmI*	1.55
A0A0D1JWV4	50S ribosomal protein L28	*rpmB*	1.55
A0A0D6GUT4	Membrane protein	*mmpL8*	1.55
A0A069FS69	Phosphocarrier protein HPr	*ptsH*	1.58
A0A033UDX3	33 kDa chaperonin	*hslO*	1.60
A0A0D1FDF3	UPF0291 protein QU38_13075	-	1.63
A0A0D6DD31	4-hydroxy-tetrahydrodipicolinate synthase	*dapA*	1.65
A0A090LZN4	Biotin carboxylase 1	*accC*	1.65
A0A0D1FG95	Deoxyadenosine kinase	*SAJPND1_00548*	1.65
A0A090LTB6	Methyltransferase small domain protein	*SAU060112_30089*	1.66
A0A0D1HT96	Strain SA-120 Contig620, whole genome shotgun sequence	-	1.69
A0A0D1H7G8	Thioredoxin	*QU38_12030*	1.69
A0A077ULL5	LPXTG surface protein	*sdrD*	1.70
A0A069G344	FeS assembly protein SufD	*sufD*	1.70
A0A0D1I822	30S ribosomal protein S20	*rpsT*	1.72
A0A0D6GKH3	FAD-dependent pyridine nucleotide-disulfide oxidoreductase	*fccB*	1.73

Accession Number	Protein Name	Gene Name	Fold Change
A0A077UL50	ATP-dependent Clp protease ATP-binding subunit	*clpX*	1.73
A0A0D1JUA6	UPF0473 protein QU38_08855	-	1.74
A0A0B6XSQ4	DNA repair/chromosome segregation ATPase	*ERS094548_01003*	1.74
A0A033UZC9	30S ribosomal protein S21	*rpsU*	1.75
A0A0D1IZY7	Superoxide dismutase	*QU38_15115*	1.75
A0A0D1HPY4	UDP-N-acetylmuramoyl-L-alanyl-D-glutamate-L-lysine ligase	*murE*	1.75
A0A0D1H3E1	Sced	*SAJPND1_02072*	1.75
A0A0B6XR64	Urease subunit beta	*ureB*	1.75
A0A0D6HMU5	FmhA protein of FemAB family	*fmhA*	1.76
A0A0D6FMN0	Putative cytosolic protein	*ERS445051_00083*	1.78
A0A0D1GUR6	Strain SA-120 Contig627, whole genome shotgun sequence	-	1.78
A0A0D1H1Q3	Glyoxalase family protein	*QU38_12370*	1.78
A0A090LXA3	Lipase/esterase LipA	*SAU060112_10612*	1.80
A0A033UV22	Translation initiation factor IF-1	*infA*	1.80
A0A090M065	Serine--tRNA ligase	*serS*	1.82
A0A0C5HY37	Glycine cleavage system H protein	*gcvH_2*	1.82
A0A0D1JMR1	Acyl carrier protein	*acpP*	1.83
A0A077USG2	Immunodominant staphylococcal antigen A	*isaA*	1.83
A0A068A921	Nitrate reductase	*narH*	1.85
A0A0D6DE64	Putative cytosolic protein	*SAJPND1_01441*	1.90
A0A0E0VPB3	Tripeptidase T	*ST398NM01_1577*	1.90
A0A090LUB5	Uncharacterized protein	-	1.95
A0A090N225	Secretory antigen	*ssaA*	1.95
A0A0B6XQV4	Alpha-amylase	*malA*	2.00
A0A0D1K117	Peptide methionine sulfoxide reductase MsrA	*msrA*	2.00
MURC	UDP-N-acetylmuramate-L-alanine ligase	*murC*	2.02
A0A0D1INB5	Arginine deiminase	*arcA*	2.07
A0A0D6HFI7	Succinyl-diaminopimelate desuccinylase	*dapE*	2.07
A0A077U2I8	FMN-dependent NADH-azoreductase	*azoR*	2.10
A0A0D1JQL5	Thioredoxin	*SAJPND1_00815*	2.11
A0A090LQD9	Putative peptidyl-prolyl cis-trans isomerase	*SAU060112_10378*	2.17
A0A090LUF7	DNA repair/chromosome segregation ATPase	*ERS445051_02217*	2.22
A0A0D6DD40	UPF0223 protein SAJPND1_01024	-	2.26
A0A0D1I7V8	Triosephosphate isomerase	*tpiA*	2.27
A0A077VA34	Uncharacterized protein	*ERS140026_01689*	2.28
A0A0D1FZV0	Strain SA-120 Contig626, whole genome shotgun sequence	*QU38_07345*	2.28
A0A077VL65	Succinyl-diaminopimelate desuccinylase	*dapE*	2.30
A0A0B6XKZ1	Sorbitol dehydrogenase	*gutB_1*	2.30
A0A0D1GRH1	Superoxide dismutase	*QU38_09165*	2.36
A0A077TYH3	Uncharacterized protein	-	2.37
A0A077UT21	Phage protein	*ERS140159_01807*	2.40
A0A0B4N811	3-hexulose-6-phosphate synthase	*CH51_02975*	2.40

Accession Number	Protein Name	Gene Name	Fold Change
A0A0D1I4N9	Strain SA-120 Contig630, whole genome shotgun sequence	-	2.41
A0A0D1HX32	Strain SA-120 Contig626, whole genome shotgun sequence	-	2.43
A0A090LTM2	5'-methylthioadenosine/S-adenosylhomocysteine nucleosidase	*pfs*	2.44
A0A0D1IW19	Biotinc arboxyl carrier protein of acetyl-CoA carboxylase	*SAJPND1_01561*	2.44
A0A0D1HYD3	Strain SA-120 Contig626, whole genome shotgun sequence	*QU38_07650*	2.46
A0A0D6WDW6	Major tail protein	*ERS445051_01394*	2.50
A0A0D1FA44	Strain SA-120 Contig617, whole genome shotgun sequence	-	2.52
A0A077UAB9	S-ribosylhomocysteine lyase	*luxS*	2.53
A0A077UBW6	O-methyltransferase family protein	*ERS140162_01570*	2.55
A0A0D1GNT7	Strain SA-120 Contig628, whole genome shotgun sequence	-	2.55
A0A0D1IPY9	Organic hydroperoxide resistance protein	*SAJPND1_00812*	2.56
A0A0D1K2T0	Putative cytosolic protein	*QU38_07185*	2.64
A0A069G4G3	Recombination protein RecR	*recR*	2.77
A0A0C5HEJ3	Endoribonuclease L-PSP	*yabJ*	2.85
A0A0D1K3I9	Cold shock protein	*QU38_11035*	2.89
A0A0D1HB94	2,3-bisphosphoglycerate-independent phosphoglycerate mutase	*gpmI*	2.90
A0A0D1H2E4	UPF0356 protein SAJPND1_01017	-	2.97
A0A0D1JVV7	Oxygen-dependent choline dehydrogenase	*betA*	3.00
A0A077UW75	YceI-like domain protein	*ERS140266_00175*	3.00
A0A0D1HIZ5	Putative cytosolic protein	*SAJPND1_01624*	3.01
A0A090LVJ3	Ornithine carbamoyltransferase	*arcB*	3.09
A0A0D1KAK6	Rhodanese-related sulfurtransferase	*SAJPND1_01707*	3.15
A0A0D1HYC8	Nitrite reductase [NAD(P)H] large subunit	*SAJPND1_02388*	3.17
A0A0E1VTG8	Oxidoreductase, FAD/FMN-binding protein	*HMPREF0776_1945*	3.20
A0A090M0E8	Molybdopterin synthase sulfur carrier subunit	*moaD*	3.23
A0A0D3Q462	Uncharacterised protein	*CH51_01290*	3.31
A0A0D1H4E0	Strain SA-120 Contig630, whole genome shotgun sequence	*QU38_15895*	3.40
G0XY48	Clumping factor A	*clfA*	3.45
A0A0E1X586	Phage major tail protein	*HMPREF0769_12240*	3.50
A0A0D3Q486	Uncharacterized protein	-	3.68
A0A033V5W3	Cold shock protein CspA	*cspA*	3.75
A0A0B6XQZ3	S-ribosylhomocysteine lyase	*luxS*	3.80
A0A0D6HBG6	UPF0435 protein ERS445051_01856	-	3.98
A0A0D1GFP6	Transcription elongation factor GreA	*greA*	4.06
A0A077UAB5	D-2-hydroxyacid dehydrogenase	*ldhD_2*	4.25
A0A0D1JZ55	Putative cytosolic protein	*SAJPND1_01294*	4.52
A0A0B6XLS6	UDP-2-acetamido-2%2C6-dideoxy-beta-L-talose 4-dehydrogenase	*ERS094548_01225*	5.15
A0A0D6G3W7	Serine-aspartate repeat-containing protein E	*sdrE*	5.16
A0A0B6XKR5	Capsular polysaccharide synthesis enzyme Cap5I	*capI*	5.40
A0A077UNT4	YozC	*ERS140026_00525*	6.35

**Accession Number**	**Protein Name**	**Gene Name**	**Fold Change**
A0A090N1L9	UDP-glucose 4-epimerase	*capD*	7.55
