# Supplementary material for: iTRAQ-Based Quantitative Proteomic Profiling of Staphylococcus aureus Under Different Osmotic Stress Conditions
Source: Front Microbiol. 2019 May 29;10:1082. doi: 10.3389/fmicb.2019.01082 (PMC6549500; doi:10.3389/fmicb.2019.01082)
Supplement: Supplementary file 5 [file Data_Sheet_5.PDF]

**Table S5** Differentially expressed proteins in the 20% NaCl group compared with the control group. The DEPs in Figure 5 with fold change ≥2 or ≤0.5 are labelled in red.

| Accession Number | Protein Name | Gene Name | Fold Change |
|---|---|---|---|
| A0A0D1IXJ7 | Gamma-hemolysin B subunit HlgB | *SAJPND1_02412* | 0.04 |
| A0A0D1J6H7 | Strain SA-120 Contig629, whole genome shotgun sequence | - | 0.05 |
| A0A0D6DCF3 | Extracellular fibrinogen-binding protein | *SAJPND1_01081* | 0.05 |
| OMP7 | 77 kDa membrane protein | *SACOL2002* | 0.04 |
| A0A0D6DK32 | Extracellular matrix binding protein | *SAJPND1_00798* | 0.05 |
| A0A0D6DFZ4 | Leukocidin S subunit LukS | *SAJPND1_01995* | 0.05 |
| A0A077U1D0 | Amino acid permease | *steT* | 0.05 |
| A0A0D6DIM1 | Ribokinase | *SAJPND1_00306* | 0.08 |
| A0A0D1JXR9 | Strain SA-120 Contig630, whole genome shotgun sequence | - | 0.09 |
| A0A0D1K3U3 | Extracellular fibrinogen-binding protein | *QU38_12095* | 0.10 |
| A7E1C4 | Enterotoxin C2 (Fragment) | *sec2* | 0.10 |
| A0A068A489 | Inhibitor | *scn_2* | 0.10 |
| A0A0B6XPE8 | Pyruvate carboxylase | *cfiB_1* | 0.10 |
| A0A090LXD5 | Uncharacterized protein | - | 0.10 |
| A0A0D1HN80 | Strain SA-120 Contig620, whole genome shotgun sequence | - | 0.10 |
| A0A0D6DGW3 | Gamma-hemolysin A subunit HlgA | *SAJPND1_02409* | 0.11 |
| SBI | Immunoglobulin-binding protein sbi | *sbi* | 0.11 |
| A0A0D6DFD4 | Beta-lactamase regulator protein | *SAJPND1_01896* | 0.11 |
| Q9AFB0 | Leukocidin LukS component (Fragment) | - | 0.11 |
| A0A0C5HVE0 | Pyruvate decarboxylase%3B Alpha-keto-acid decarboxylase | *ipdC* | 0.11 |
| A0A090N157 | Alpha-hemolysin | *hly* | 0.11 |
| A0A0D6GYJ2 | Alanine dehydrogenase | *ald1* | 0.11 |
| A0A0B6XPE8 | Pyruvate carboxylase | *cfiB_1* | 0.12 |
| A0A0B6XL12 | Pseudouridine-5'-phosphate glycosidase | *psuG* | 0.13 |
| A0A0D1I054 | L-lactate dehydrogenase | *ldh* | 0.13 |
| A0A0B6XMG3 | Argininosuccinate synthase | *argG* | 0.14 |
| A0A0D1I054 | L-lactate dehydrogenase | *ldh* | 0.14 |
| A0A0E1VRD5 | Threonine ammonia-lyase | *ilvA* | 0.14 |
| A0A0E0VS19 | Carboxylic ester hydrolase | *ST398NM01_2504* | 0.14 |
| A0A0B6XL12 | Pseudouridine-5'-phosphate glycosidase | *psuG* | 0.14 |
| ADH | Alcohol dehydrogenase | *adh* | 0.14 |
| A0A0B6XMG3 | Argininosuccinate synthase | *argG* | 0.14 |
| A0A0C5HSI2 | Gamma-hemolysin component B | *ERS445051_01979* | 0.15 |
| A0A0D6DGG6 | Uncharacterized protein | - | 0.15 |
| A0A077TWE9 | Lipoprotein | *ERS094548_00195* | 0.15 |
| A0A0E1VQA6 | Immunoglobulin-binding protein sbi | *sbi* | 0.15 |
| A7E1C4 | Enterotoxin C2 (Fragment) | *sec2* | 0.15 |
| A0A0E0VS19 | Carboxylic ester hydrolase | *ST398NM01_2504* | 0.15 |
| Q7DK27 | ABC transporter ATP-binding protein | *stpA* | 0.15 |

| Accession Number | Protein Name | Gene Name | Fold Change |
|---|---|---|---|
| A0A0D6DN91 | Thymidine phosphorylase | *SAJPND1_02113* | 0.15 |
| A0A0D1HD00 | Universal stress protein | *uspA* | 0.15 |
| A0A090LWI3 | Argininosuccinate lyase | *argH* | 0.15 |
| A0A077U1A4 | Oligopeptide transporter putative substrate binding domain protein | *opp-1A* | 0.15 |
| A0A0E1VRD5 | Threonine ammonia-lyase | *ilvA* | 0.15 |
| A0A0C5HVE0 | Pyruvate decarboxylase%3B Alpha-keto-acid decarboxylase | *ipdC* | 0.16 |
| A0A0D1JQX0 | Strain SA-120 Contig628, whole genome shotgun sequence | - | 0.17 |
| A0A090LUS6 | Lipoprotein | *yehR* | 0.17 |
| A0A0C5HSI2 | Gamma-hemolysin component B | *ERS445051_01979* | 0.18 |
| A0A090LW57 | ABC transporter, permease protein | *SAU060112_100003* | 0.18 |
| A0A0C2HZY8 | Pyrimidine-nucleoside phosphorylase | *pdp* | 0.18 |
| A0A090LW57 | ABC transporter, permease protein | *SAU060112_100003* | 0.18 |
| A0A077U1A4 | Oligopeptide transporter putative substrate binding domain protein | *opp-1A* | 0.19 |
| A0A0D1G5I0 | Strain SA-120 Contig630, whole genome shotgun sequence | - | 0.19 |
| A0A0E1VKR4 | ABC transporter, ATP-binding protein | *HMPREF0776_1978* | 0.19 |
| A0A090LUS6 | Lipoprotein | *yehR* | 0.19 |
| A0A0D6DDK7 | Uncharacterized protein | - | 0.19 |
| A0A0C5HUH7 | Carbamate kinase | *arcC* | 0.20 |
| A0A0B6XTB4 | N-acetylmuramoyl-L-alanine amidase%2C family 4 | *ERS094548_01923* | 0.20 |
| A0A077U4E2 | 2-oxoglutarate/malate translocator-like protein | *ttdT* | 0.20 |
| A0A090N246 | Formate/nitrite transporter | *nirC* | 0.20 |
| A0A077U927 | Pseudouridine-5'-phosphate glycosidase | *psuG* | 0.20 |
| A0A077VAK0 | Hexose phosphate transport protein | *uhpT* | 0.20 |
| A0A0D6DN91 | Thymidine phosphorylase | *SAJPND1_02113* | 0.20 |
| A0A0D6GIM9 | Autolysin | *atl_1* | 0.20 |
| A0A0D6DA07 | Iron-sulfur cluster repair protein ScdA | *scdA* | 0.20 |
| A0A0B6XP33 | Oligopeptide transport ATP-binding protein oppD | *oppD_1* | 0.21 |
| A0A077VAK0 | Hexose phosphate transport protein | *uhpT* | 0.21 |
| A0A0B6XTB4 | N-acetylmuramoyl-L-alanine amidase%2C family 4 | *ERS094548_01923* | 0.21 |
| A0A0C2HZY8 | Pyrimidine-nucleoside phosphorylase | *pdp* | 0.22 |
| A0A0D1GJW4 | Arsenate reductase family protein | *QU38_11110* | 0.22 |
| A0A0D1K2N9 | GlnQ protein | *glnQ* | 0.22 |
| A0A0D6HPJ5 | Lantibiotic transport ATP-binding protein | *ecsA_3* | 0.23 |
| A0A0D6GIM9 | Autolysin | *atl_1* | 0.23 |
| A0A0D6DA07 | Iron-sulfur cluster repair protein ScdA | *scdA* | 0.23 |
| A0A090LSD7 | Uncharacterized protein | - | 0.23 |
| A0A0D1HUP1 | Nucleoside transporter | *QU38_09615* | 0.23 |
| A0A077V7K3 | ABC transporter substrate-binding protein | *psaA* | 0.23 |
| A0A033UCK9 | Biotin synthase | *bioB* | 0.23 |
| A0A090LQD2 | Glycerophosphoryl diester phosphodiesterase | *glpQ* | 0.23 |

| Accession Number | Protein Name | Gene Name | Fold Change |
|---|---|---|---|
| A0A0D1GTD3 | Strain SA-120 Contig622, whole genome shotgun sequence | - | 0.24 |
| Q9S2Z4 | Cell surface protein map-w | *map-w* | 0.24 |
| A0A090LUS7 | AraC family transcription regulator | *araC* | 0.25 |
| A0A090LQ06 | Uncharacterized protein | - | 0.25 |
| A0A0D6FS99 | Putative polyribitolphosphotransferase | *tagF_1* | 0.25 |
| A0A0B6XNX0 | Alanine dehydrogenase | *ald2_1* | 0.25 |
| A0A0C5HYQ5 | SepS16B protein | *sepS16B* | 0.25 |
| A0A0D1GJW4 | Arsenate reductase family protein | *QU38_11110* | 0.25 |
| A0A0D1H8B0 | Threonine-tRNA ligase | *thrS* | 0.25 |
| A0A0C5HYQ5 | SepS16B protein | *sepS16B* | 0.25 |
| A0A090N246 | Formate/nitrite transporter | *nirC* | 0.25 |
| A0A077U4E2 | 2-oxoglutarate/malate translocator-like protein | *ttdT* | 0.25 |
| A0A0B6XNX0 | Alanine dehydrogenase | *ald2_1* | 0.25 |
| A0A033UCK9 | Biotin synthase | *bioB* | 0.25 |
| A0A0C5HUH7 | Carbamate kinase | *arcC* | 0.25 |
| A0A090LWP1 | HTH-type transcriptional regulator SarS | *sarS* | 0.26 |
| A0A077TYC4 | Similar to putative sodium/glucose cotransporter | *sglT* | 0.26 |
| A0A0B6XKT1 | Aldehyde-alcohol dehydrogenase | *adhE* | 0.26 |
| A0A090M2J3 | Uncharacterized protein | - | 0.26 |
| A0A090LR99 | CobW/HypB/UreG, nucleotide-binding domain protein | *SAU060112_10579* | 0.27 |
| A0A077U8P9 | Membrane spanning protein | *ERS140026_02521* | 0.27 |
| A0A077VSV4 | Peptide ABC transporter permease | *oppB_1* | 0.27 |
| A0A077TT97 | DNA-damage repair protein | *umuC* | 0.27 |
| A0A033V8J0 | Pyruvate carboxylase | *V070_00063* | 0.27 |
| A0A077U8P9 | Membrane spanning protein | *ERS140026_02521* | 0.27 |
| A0A0B6XP33 | Oligopeptide transport ATP-binding protein oppD | *oppD_1* | 0.27 |
| A0A090LVY0 | Aspartate carbamoyltransferase | *pyrB* | 0.28 |
| A0A0D1H6Z1 | Nitric oxide synthase oxygenase | *QU38_06960* | 0.28 |
| A0A0D1JKL8 | Carbamate kinase | *QU38_12170* | 0.28 |
| A0A077V7K3 | ABC transporter substrate-binding protein | *psaA* | 0.28 |
| A0A0D1JA14 | Esterase | *QU38_16465* | 0.28 |
| A0A0D1HUP1 | Nucleoside transporter | *QU38_09615* | 0.29 |
| A0A0D1HEF4 | 6,7-dimethyl-8-ribityllumazine synthase | *ribH* | 0.29 |
| A0A0D1HQ98 | Histidine kinase | *QU38_10565* | 0.29 |
| A0A077VMN9 | Peptide methionine sulfoxide reductase MsrA | *msrA_1* | 0.29 |
| A0A0D1JLL3 | Adenosylmethionine-8-amino-7-oxononanoate aminotransferase | *bioA* | 0.29 |
| A0A0D6FNQ5 | Immunoglobulin G-binding protein A | *spa* | 0.29 |
| A0A0D1HEF4 | 6,7-dimethyl-8-ribityllumazine synthase | *ribH* | 0.29 |
| A0A090LQD2 | Glycerophosphoryl diester phosphodiesterase | *glpQ* | 0.29 |
| A0A0A0Q4N9 | HtrA-like serine protease | *htrA1* | 0.30 |
| A0A077V9M9 | Response regulator SaeR | *saeR* | 0.30 |

| Accession Number | Protein Name | Gene Name | Fold Change |
|---|---|---|---|
| A0A077VMN9 | Peptide methionine sulfoxide reductase MsrA | *msrA_1* | 0.30 |
| A0A077UDD9 | 3-ketoacyl-CoA thiolase%3B Acetyl-CoA acetyltransferase | *ERS140248_02364* | 0.30 |
| A0A077VSV4 | Peptide ABC transporter permease | *oppB_1* | 0.30 |
| A0A090LR99 | CobW/HypB/UreG, nucleotide-binding domain protein | *SAU060112_10579* | 0.30 |
| A0A068W8S1 | Ribosomal RNA large subunit methyltransferase H | *orfX* | 0.30 |
| A0A090LVY0 | Aspartate carbamoyltransferase | *pyrB* | 0.30 |
| A0A0B6XKT1 | Aldehyde-alcohol dehydrogenase | *adhE* | 0.30 |
| A0A077TYC4 | Similar to putative sodium/glucose cotransporter | *sglT* | 0.30 |
| A0A0D1HCV5 | Uncharacterized protein | - | 0.31 |
| A0A090M1C4 | Dihydroorotate dehydrogenase (quinone) | - | 0.31 |
| A0A0D6FNQ5 | Immunoglobulin G-binding protein A | *spa* | 0.31 |
| A0A090LWP1 | HTH-type transcriptional regulator SarS | *sarS* | 0.31 |
| A0A0C5HTL6 | Dihydroxyacetone kinase | *dhaL* | 0.31 |
| A0A0A0Q4N9 | HtrA-like serine protease | *htrA* | 0.32 |
| A0A0D1JGI6 | 2-C-methyl-D-erythritol 4-phosphate cytidylyltransferase | *ispD* | 0.32 |
| A0A077U988 | Pyrimidine nucleoside transport protein | *nupC_2* | 0.32 |
| A0A0C5HMT4 | GntR family transcriptional regulator | *treR_1* | 0.32 |
| A0A0D1JRN5 | Strain SA-120 Contig627, whole genome shotgun sequence | - | 0.32 |
| A0A077U7R1 | Cytochrome d ubiquinol oxidase subunit I | *cydA* | 0.32 |
| A0A068W8S1 | Ribosomal RNA large subunit methyltransferase H | *orfX* | 0.32 |
| A0A090N1L4 | Diacetyl reductase ((S)-acetoin forming) | *butA* | 0.33 |
| A0A077U2Q8 | Quinol oxidase polypeptide I QoxB | *qoxB* | 0.33 |
| A0A0D1GVJ7 | Poly (Glycerol-phosphate) alpha-glucosyltransferase | *SAJPND1_00558* | 0.33 |
| A0A0D1HQ98 | Histidine kinase | *QU38_10565* | 0.33 |
| A0A090M1C4 | Dihydroorotate dehydrogenase (quinone) | *pyrD* | 0.33 |
| A0A077U2Q8 | Quinol oxidase polypeptide I QoxB | *qoxB* | 0.33 |
| T1Y8N9 | Ornithine carbamoyltransferase | *SAKOR_01090* | 0.34 |
| A0A0B6XNE5 | Hydrolase (HAD superfamily) | *ERS094548_00694* | 0.34 |
| A0A0D1IIH6 | Uncharacterized protein | - | 0.34 |
| A0A0D1HUW5 | Threonine synthase | *SAJPND1_01270* | 0.34 |
| A0A077TT97 | DNA-damage repair protein | *umuC* | 0.35 |
| A0A0D3QBH3 | Peptide synthetase | *grsB* | 0.35 |
| A0A077U988 | Pyrimidine nucleoside transport protein | *nupC_2* | 0.35 |
| A0A0D1JLL3 | Adenosylmethionine-8-amino-7-oxononanoate aminotransferase | *bioA* | 0.35 |
| A0A077V9M9 | Response regulator SaeR | *saeR* | 0.35 |
| A0A077U7R1 | Cytochrome d ubiquinol oxidase subunit I | *cydA* | 0.35 |
| A0A0C5HMT4 | GntR family transcriptional regulator | *treR_1* | 0.35 |
| A0A0D1H6Z1 | Nitric oxide synthase oxygenase | *QU38_06960* | 0.35 |
| A0A0D6DD63 | Peptide methionine sulfoxide reductase MsrA | *msrA* | 0.35 |
| A0A0E1VQA5 | MOSC domain protein | *HMPREF0776_0387* | 0.35 |
| A0A069G7V7 | Putative N-acetyltransferase YedL | *CO98_1080* | 0.35 |

| Accession Number | Protein Name | Gene Name | Fold Change |
|---|---|---|---|
| A0A077VDA6 | Lipoprotein | *metQ_1* | 0.35 |
| A0A0D1GBS9 | Signal transduction protein TRAP | *SAJPND1_01796* | 0.36 |
| A0A0D1INB5 | Arginine deiminase | *arcA* | 0.36 |
| A0A0D1JT33 | Cardiolipin synthase | *QU38_06435* | 0.36 |
| A0A077TYE1 | Uncharacterized protein | - | 0.36 |
| A0A090LXY5 | Uncharacterized protein | - | 0.36 |
| A0A077TYE7 | Uncharacterized protein conserved in bacteria | *ERS140095_00284* | 0.38 |
| A0A090M283 | Catalase | *katA* | 0.38 |
| A0A0D1JGI6 | 2-C-methyl-D-erythritol 4-phosphate cytidylyltransferase | *ispD* | 0.38 |
| A0A0D6DIR5 | Protein-arginine kinase | *mcsB* | 0.38 |
| X5IX38 | DNA-binding protein | *SAU060112_10479* | 0.38 |
| A0A090LW12 | Poly(Glycerol-phosphate) alpha-glucosyltransferase | *SAU060112_10705* | 0.38 |
| A0A0D1G1J9 | Cytochrome c oxidase polypeptide II | *SAJPND1_00987* | 0.38 |
| A0A077UIM6 | Polysaccharide intercellular adhesin (PIA) biosynthesis N-glycosyltransferase IcaA | *icaA* | 0.38 |
| A0A090LPS1 | Uncharacterized protein | - | 0.38 |
| A0A0D1GD88 | 2,3-bisphosphoglycerate-dependent phosphoglycerate mutase | *gpmA* | 0.39 |
| A0A090N1L4 | Diacetyl reductase ((S)-acetoin forming) | *butA* | 0.39 |
| A0A090N1D1 | ABC transporter | *yfmR* | 0.39 |
| A0A0E1XJY5 | Abi-like protein | *HMPREF0769_10093* | 0.39 |
| A0A0D1GC10 | Strain SA-120 Contig629, whole genome shotgun sequence | - | 0.39 |
| A0A0D6GKV9 | UPF0637 protein ERS157365_00537 | - | 0.39 |
| A0A0D6GUT2 | Cardiolipin synthase | - | 0.39 |
| A0A0D6DNZ9 | Extracellular matrix binding protein | *SAJPND1_02136* | 0.39 |
| A0A0D1GN53 | Strain SA-120 Contig628, whole genome shotgun sequence | - | 0.39 |
| A0A090LZI4 | Uncharacterized protein | - | 0.39 |
| A0A0B6XN73 | 3-oxoadipate enol-lactonase | *dehH1* | 0.40 |
| A0A090M057 | Putative ATP-dependent helicase DinG homolog | *dinG* | 0.40 |
| A0A090N1W4 | Uncharacterized AAA domain-containing protein YrvN | - | 0.40 |
| A0A0E1AE46 | Phosphoenolpyruvate-dihydroxyacetone phosphotransferase, dihydroxyacetone binding subunit DhaK | *SAZ172_0661* | 0.40 |
| A0A090LVS1 | Potassium-transporting ATPase B chain | *kdpB* | 0.40 |
| A0A090LXT5 | Peptidase C45 | *SAU060112_110028* | 0.40 |
| A0A077UJH0 | DNA-binding response regulator | *bvgA* | 0.40 |
| A0A0D1EST2 | Pyruvate formate-lyase-activating enzyme | *QU38_14695* | 0.40 |
| A0A077UZP7 | Transcriptional repressor NrdR | *nrdR* | 0.41 |
| A0A0D1FF77 | Coenzyme A disulfide reductase | *cdr* | 0.41 |
| A0A077UJ82 | Uncharacterized protein | - | 0.41 |
| A0A0B6XL00 | Lipoprotein | *metQ_1* | 0.41 |
| A0A090LR26 | Uncharacterized oxidoreductase YcsN | - | 0.41 |
| A0A077U7Z6 | Antiholin-like protein LrgA | *lrgA* | 0.41 |
| A0A090M2D6 | Oligoendopeptidase F | *SAU060112_70075* | 0.42 |

| Accession Number | Protein Name | Gene Name | Fold Change |
|---|---|---|---|
| A0A0D6GK28 | Uncharacterized protein | - | 0.42 |
| A0A077W0Q5 | UPF0365 protein ERS140167_00916 | - | 0.42 |
| A0A0B6XPU6 | Acetyltransferase AcuA%2C acetyl-CoA synthetase inhibitor | *acuA* | 0.42 |
| A0A0B6XP73 | Lipoate--protein ligase | *lplJ_2* | 0.43 |
| A0A0B6XNT6 | Bifunctional ligase/repressor BirA | *birA* | 0.43 |
| A0A0D6DHW6 | Glycosyltransferase | *SAJPND1_00237* | 0.43 |
| A0A090LTY1 | Uncharacterized protein | - | 0.43 |
| A0A0E7DTW4 | Putative lipoprotein | *ERS157365_00280* | 0.44 |
| A0A090LX90 | Probable transcriptional regulatory protein yeeN | *yeeN* | 0.44 |
| A0A0E1VQ76 | Biotin synthase | *bioB* | 0.44 |
| A0A0B6XKT2 | Ribose operon repressor%2C putative | *degA* | 0.45 |
| A0A0B6XPW9 | Sodium/proline symporter | *putP* | 0.45 |
| A0A0D6HWL4 | ATP-dependent helicase/deoxyribonuclease subunit B | *addB* | 0.45 |
| A0A0E1VI43 | Carbamoyl-phosphate synthase (glutamine-hydrolyzing) | *carB* | 0.45 |
| A0A069G2X1 | Methicillin resistance regulatory protein MecI | *mecI* | 0.45 |
| A0A077U153 | Gluconate permease | *gntP* | 0.45 |
| A0A077U0H9 | Pantothenate synthetase | *panC* | 0.45 |
| A0A0D6HMD3 | General stress protein 26 | *ydaG* | 0.45 |
| A0A0B6XRK6 | Nicotinate phosphoribosyltransferase | *ERS094548_02369* | 0.45 |
| A0A0D6FNA4 | ATPase | *CH51_00375* | 0.45 |
| A0A0D1HUW5 | Threonine synthase | *SAJPND1_01270* | 0.45 |
| A0A077UDM1 | Manganese ABC transporter%2C ATP-binding protein SitB | *sitB* | 0.45 |
| A0A077UDM4 | Ketol-acid reductoisomerase | *ilvC* | 0.45 |
| A0A090LZD2 | Putative permease of an ABC transporter | *ybbM* | 0.45 |
| A0A077UG02 | Acid phosphatase | *hel* | 0.46 |
| A0A077TZL7 | Modification methylase MboII | *mboIIM* | 0.46 |
| A0A090N216 | Uncharacterized protein | - | 0.46 |
| A0A0D1IUP6 | Strain SA-120 Contig628, whole genome shotgun sequence | - | 0.46 |
| A0A0D1GU53 | Uncharacterized protein | - | 0.47 |
| A0A0D1HYC8 | Nitrite reductase [NAD(P)H] large subunit | *SAJPND1_02388* | 0.47 |
| A0A0D1KA16 | Strain SA-120 Contig620, whole genome shotgun sequence | - | 0.48 |
| A0A0D1HKS5 | Chromosomal replication initiator protein DnaA | *dnaA* | 0.48 |
| A0A0D1FNA8 | Uncharacterized protein | - | 0.48 |
| A0A0E1AMS3 | Uncharacterized protein | - | 0.49 |
| A0A0D6DGY8 | Uroporphyrin-III C-methyltransferase | *SAJPND1_02386* | 0.49 |
| A0A080V4N7 | Membrane spanning protein | *yhhT_1* | 0.49 |
| A0A090LXQ9 | Uncharacterized protein | - | 0.49 |
| A0A0D6DGT6 | Cystine-binding protein | *SAJPND1_02402* | 0.50 |
| A0A0D1IB15 | Strain SA-120 Contig624, whole genome shotgun sequence | - | 0.50 |
| A0A090LRF4 | Uncharacterized protein | - | 0.50 |
| A0A077VJX6 | Deblocking aminopeptidase | *ysdC_1* | 0.50 |
| A0A0D1I5J3 | Ribulose-phosphate 3-epimerase | *QU38_12445* | 0.50 |

| Accession Number | Protein Name | Gene Name | Fold Change |
|---|---|---|---|
| A0A0D1J363 | Uracil phosphoribosyltransferase | *upp* | 0.50 |
| A0A0E1VLF4 | TIGR01777 family protein | *HMPREF0776_1780* | 0.50 |
| A0A090LUN7 | Urocanate hydratase | *hutU* | 0.50 |
| A0A077UP25 | Membrane-bound serine protease | *ERS140159_00273* | 0.50 |
| A0A0D1IBW0 | Strain SA-120 Contig620, whole genome shotgun sequence | - | 0.50 |
| A0A090N1M7 | Type-1 restriction enzyme R protein | *hsdR* | 0.50 |
| A0A077TXE8 | L-threonine 3-dehydrogenase | *ERS140248_00818* | 0.50 |
| A0A0D6DGE5 | Acriflavin resistance plasma membrane protein | *SAJPND1_02233* | 0.50 |
| A0A0D6DPK7 | Staphyloxanthin biosynthesis protein CrtP | *SAJPND1_02566* | 0.50 |
| A0A0D1K8W2 | Bifunctional protein PyrR | *pyrR* | 0.50 |
| A0A0B6XPT4 | D-3-phosphoglycerate dehydrogenase | *serA* | 0.50 |
| A0A0D6DDT0 | Homoserine dehydrogenase | *SAJPND1_01269* | 0.50 |
| A0A077UPQ0 | Antiholin-like protein LrgB | *lrgB* | 0.50 |
| A0A090LX68 | Tagatose-6-phosphate kinase | *fruK* | 0.50 |
| A0A0D1G9T6 | Tautomerase | *QU38_13190* | 0.50 |
| A0A077V1P6 | Methionine ABC transporter ATP-binding protein | *ybbL* | 0.50 |
| A0A077U7R0 | 3-isopropylmalate dehydratase large subunit | *leuC* | 0.50 |
| A0A0B6XPL1 | Aldo/keto reductase family protein | *yvgN* | 0.50 |
| A0A090LXW3 | Ferritin | *ftnA* | 0.50 |
| A0A090LVJ3 | Ornithine carbamoyltransferase | *arcB* | 0.50 |
| A0A0D6HVT0 | ATP-dependent helicase/nuclease subunit A | *addA* | 0.51 |
| A0A0D1H334 | Putative kinase associated protein B family protein | *QU38_11380* | 0.51 |
| A0A090LWR0 | Phosphoglycerate mutase | *gpmA_1* | 0.51 |
| A0A0E1VIJ7 | HTH-type transcriptional regulator rot | *HMPREF0776_2797* | 0.51 |
| A0A0D1JYJ4 | Strain SA-120 Contig628, whole genome shotgun sequence | - | 0.51 |
| A0A077V172 | Respiratory nitrate reductase gamma chain | *narI* | 0.52 |
| A0A069FW70 | Fructose-1,6-bisphosphatase class 3 | *fbp* | 0.52 |
| A0A090LXB7 | Luciferase-like monooxygenase | *SAU060112_20273* | 0.52 |
| A0A0D1G640 | Arginine repressor | *argR* | 0.52 |
| A0A077U0D7 | Protein of pXO2-46 | *ERS140095_00709* | 0.53 |
| A0A0D1H5M4 | Transaldolase | *QU38_04770* | 0.53 |
| A0A0D1HBY5 | Pyridoxal 5'-phosphate synthase subunit PdxT | *pdxT* | 0.54 |
| A0A090LUF4 | Ribose-5-phosphate isomerase A | *rpiA* | 0.54 |
| A0A090LRU6 | Regulatory protein RecX | *recX* | 0.54 |
| A0A077UNV8 | Glycosyltransferase | *pimB* | 0.54 |
| A0A077UUV1 | Hydrogen peroxide-inducible genes activator | *oxyR* | 0.54 |
| A0A0E1AE25 | Phosphoglycolate phosphatase | *SAZ172_0574* | 0.55 |
| A0A0D1HKP9 | Arginase | *SAJPND1_02140* | 0.55 |
| A0A0D1I632 | Carbamoyl-phosphate synthase small chain | *carA* | 0.55 |
| A0A090LUN9 | 6-carboxyhexanoate-CoA ligase | *bioW* | 0.55 |
| A0A090LVM4 | Poly-beta-1,6-N-acetyl-D-glucosamine N-deacetylase | *icaB* | 0.55 |
| A0A090LVR9 | Uncharacterized protein | - | 0.55 |

| Accession Number | Protein Name | Gene Name | Fold Change |
|---|---|---|---|
| A0A0D1GS08 | GTP-sensing transcriptional pleiotropic repressor CodY | *codY* | 0.55 |
| A0A090LUQ0 | Glycerate kinase | *glxK* | 0.55 |
| A0A0D6GUT9 | Dehydrosqualene desaturase | *crtN* | 0.55 |
| A0A077UJ58 | Pyridoxal 5'-phosphate synthase subunit PdxS | *pdxS* | 0.56 |
| A0A0B6XQC4 | Serine hydroxymethyltransferase | *glyA* | 0.56 |
| A0A0B6XMC2 | Lipoprotein | *metQ_2* | 0.56 |
| A0A077UYA4 | Glycerol uptake facilitator protein | *glpF* | 0.56 |
| A0A0D6DAW8 | Long-chain-fatty-acid-CoA ligase | *SAJPND1_00209* | 0.56 |
| A0A0D1HXA7 | D-alanine aminotransferase | *SAJPND1_01699* | 0.56 |
| A0A0D3QB94 | Fructose-1,6-bisphosphatase class 3 | *fbp* | 0.56 |
| A0A0D1IG60 | 2-dehydropantoate 2-reductase | *SAJPND1_02437* | 0.56 |
| A0A077UPJ4 | Phosphopentomutase | *deoB* | 0.57 |
| A0A090N1I7 | Putative aldehyde dehydrogenase YwdH | *ywdH* | 0.57 |
| A0A077UPJ4 | Phosphopentomutase | *deoB* | 0.57 |
| A0A0D1JMD6 | Ribokinase | *QU38_14445* | 0.57 |
| A0A0D1HYD3 | Strain SA-120 Contig626, whole genome shotgun sequence | - | 0.57 |
| A0A077VJX6 | Deblocking aminopeptidase | *ysdC_1* | 0.57 |
| A0A0D1JPH9 | Strain SA-120 Contig629, whole genome shotgun sequence | - | 0.58 |
| A0A0D1HD94 | Serine acetyltransferase | *sat* | 0.58 |
| A0A0D1GS08 | GTP-sensing transcriptional pleiotropic repressor CodY | *codY* | 0.58 |
| A0A090LSG7 | L-lactate dehydrogenase | *ldhA* | 0.58 |
| A0A0D1K078 | Nitrite reductase [NAD(P)H] small subunit | *QU38_07730* | 0.59 |
| A0A077U7M7 | Two component transcriptional regulator VraR%2C LuxR family | *vraR_2* | 0.59 |
| A0A0D3Q7P5 | 3-phosphoshikimate 1-carboxyvinyltransferase | *aroA_2* | 0.59 |
| A0A0D1HPS7 | SdhB protein | *sdhB* | 0.59 |
| A0A090LVS0 | DNA replication and repair protein RecF | *recF* | 0.59 |
| A0A090LUN2 | Na+/H+ antiporter family protein | *SAU060112_40163* | 0.60 |
| A0A0E0VRM5 | TenA | *ST398NM01_2137* | 0.60 |
| A0A090M1B5 | KR domain protein | *SAU060112_40411* | 0.60 |
| EBH | Extracellular matrix-binding protein ebh | *ebh* | 0.60 |
| A0A077USJ8 | Putative lipoprotein | *ERS140159_00666* | 0.60 |
| A0A090LYD3 | Uncharacterized protein | - | 0.60 |
| A0A0B6XKQ5 | Deoxyribose-phosphate aldolase | *dra_1* | 0.60 |
| A0A090LVS0 | DNA replication and repair protein RecF | *recF* | 0.60 |
| A0A077UYA4 | Glycerol uptake facilitator protein | *glpF* | 0.60 |
| A0A0D1JK42 | Methionine import ATP-binding protein MetN | *metN* | 0.60 |
| A0A0D6GUT9 | Dehydrosqualene desaturase | *crtN* | 0.60 |
| A0A090M1B5 | KR domain protein | *SAU060112_40411* | 0.60 |
| A0A090LYP3 | Protein EsaA | *esaA* | 0.60 |
| A0A0D1H4D9 | Strain SA-120 Contig627, whole genome shotgun sequence | - | 0.61 |
| A0A077VIK6 | Monofunctional glycosyltransferase | *mgt* | 0.61 |

| Accession Number | Protein Name | Gene Name | Fold Change |
|---|---|---|---|
| A0A0D1JK42 | Methionine import ATP-binding protein MetN | *metN* | 0.61 |
| A0A0D1H739 | Strain SA-120 Contig625, whole genome shotgun sequence | - | 0.61 |
| A0A0B6XQ25 | Aspartokinase | *lysC2* | 0.61 |
| A0A077UG73 | Lactose phosphotransferase system repressor | *lacR* | 0.61 |
| A0A0D1HPS7 | SdhB protein | *sdhB* | 0.62 |
| A0A0B6XKQ5 | Deoxyribose-phosphate aldolase | *dra_1* | 0.62 |
| A0A0E1AIF3 | Branched-chain alpha-keto acid dehydrogenase, E1 component, beta subunit | *bfmBAB* | 0.62 |
| A0A0D1HFA7 | Type II pantothenate kinase | *coaW* | 0.62 |
| A0A0A0R7J1 | RepL | - | 0.62 |
| A0A0D6DK53 | Glyoxylatereductase | *SAJPND1_00846* | 0.62 |
| A0A0D1FJV3 | Glucose-6-phosphate 1-dehydrogenase | *zwf* | 0.62 |
| A0A0D1I792 | Strain SA-120 Contig624, whole genome shotgun sequence | - | 0.62 |
| A0A077U8X5 | ResD | *srrA* | 0.63 |
| A0A0E1VKG9 | Endonuclease MutS2 | *mutS2* | 0.63 |
| A0A077U597 | Histidine kinase | *walK* | 0.63 |
| A0A090LUE4 | Pseudouridine synthase | *ylyB* | 0.63 |
| A0A077UQK7 | Small integral membrane protein | *ERS140095_02392* | 0.64 |
| A0A077UJ41 | 3-methyl-2-oxobutanoate hydroxymethyltransferase | *panB* | 0.64 |
| A0A077VQ84 | NADP-dependent malic enzyme | *maeB* | 0.64 |
| A0A077U828 | Serine-protein kinase RsbW | *rsbW* | 0.64 |
| A0A0D6DB70 | Staphylococcal accessory regulator-like protein | *SAJPND1_00668* | 0.64 |
| A0A0D1FJV3 | Glucose-6-phosphate 1-dehydrogenase | *zwf* | 0.64 |
| A0A0D1HST0 | Putative sporulation transcription regulator WhiA | *whiA* | 0.65 |
| A0A0D6GTK5 | 3-hydroxy-3-methylglutaryl coenzyme A reductase | *mvaA* | 0.65 |
| A0A090LZA4 | Chorismate mutase I / 2-keto-3-deoxy-D-arabino-heptulosonate-7-phosphate synthase | *aroA* | 0.65 |
| A0A077TXN4 | Ncharacterized conserved protein | *ERS140095_00443* | 0.65 |
| A0A090N186 | Putative sodium/proton-dependent alanine carrier protein YrbD | *yrbD* | 0.65 |
| A0A0D6DMA3 | 2-succinylbenzoate--CoA ligase | *menE* | 0.66 |
| A0A0D1FA95 | Negative regulator of genetic competence clpC | *QU38_09635* | 0.66 |
| A0A0B6XKE6 | Type I restriction-modification system DNA methylase | *hsdM* | 0.66 |
| A0A0D1HJP2 | Ribosomal large subunit pseudouridine synthase D | *SAJPND1_01814* | 0.67 |
| A0A0B6XRG6 | Teichoic acid export ATP-binding protein TagH | *tagH_2* | 0.67 |
| A0A0D1H8Y0 | Glucose-6-phosphate isomerase | *pgi* | 0.67 |
| A0A0D6DMP1 | Putative exported protein | *SAJPND1_01686* | 0.67 |
| A0A0D6DK53 | Glyoxylatereductase | *SAJPND1_00846* | 0.67 |
| A0A0E1VKG9 | Endonuclease MutS2 | *mutS2* | 0.67 |
| A0A0D1HJP2 | Ribosomal large subunit pseudouridine synthase D | *SAJPND1_01814* | 0.67 |
| A0A0C5HUP1 | Endoglucanase M | *ysdC_2* | 0.67 |
| A0A0E1AI63 | Peptide methionine sulfoxide reductase MsrB | *msrB* | 0.67 |
| A0A0E1AGM7 | PTS system, glucose-specific IIA component | *SAZ172_1435* | 0.67 |

| Accession Number | Protein Name | Gene Name | Fold Change |
|---|---|---|---|
| A0A090N275 | Transcriptional regulator, TetR family | *SAU060112_40385* | 0.68 |
| A0A090LV62 | D-lactate dehydrogenase | *ldhD* | 0.68 |
| A0A0D1HWP8 | Dephospho-CoA kinase | *coaE* | 0.68 |
| A0A0B6XMX7 | DNA mismatch repair protein MutL | *mutL* | 0.68 |
| A0A0D1JNT4 | ATP synthase subunit alpha | *atpA* | 0.68 |
| A0A0D1K6G2 | Putative NmrA family protein | *SAJPND1_02580* | 0.68 |
| A0A0D1I997 | Strain SA-120 Contig626, whole genome shotgun sequence | - | 0.68 |
| A0A0D1EZS5 | Proline dehydrogenase | *SAJPND1_01717* | 0.68 |
| A0A090LTX6 | HTH-type transcriptional regulator MalR | *malR* | 0.69 |
| A0A077U2I8 | FMN-dependent NADH-azoreductase | *azoR* | 0.69 |
| A0A0D1J368 | ATP synthase subunit b | *atpF* | 0.69 |
| A0A077U8C2 | ATP synthase subunit beta | *atpD* | 0.69 |
| A0A068DT57 | Phosphoesterase%2C DHH family protein | *nrnA_1* | 0.70 |
| A0A0D1F682 | Staphylococcal accessory regulator-like protein SarV | *QU38_05105* | 0.70 |
| A0A0E8GI61 | GTPase Der | *engA* | 0.70 |
| A0A090N2D7 | Low molecular weight protein-tyrosine-phosphatase PtpB | *ptpB* | 0.70 |
| A0A0D6DD85 | DNA Polymerase X family | *SAJPND1_01069* | 0.70 |
| A0A090LY09 | GTPase Obg | *obg* | 0.70 |
| A0A077UBB6 | Type I restriction modification DNA specificity domain protein | *hsdS* | 0.70 |
| A0A0D6DMA3 | 2-succinylbenzoate-CoA ligase | *menE* | 0.70 |
| A0A0D1JZP5 | DNA repair protein RecN | *QU38_09335* | 0.70 |
| A0A077U1Q7 | Oxidoreductase%2C aldo/keto reductase family | *ERS140248_01341* | 0.70 |
| A0A077UBB6 | Type I restriction modification DNA specificity domain protein | *hsdS* | 0.70 |
| A0A0C5HTQ6 | Oxidoreductase of aldo/keto reductase family%2C subgroup 1 | *yvgN_1* | 0.70 |
| A0A090N2D7 | Low molecular weight protein-tyrosine-phosphatase PtpB | *ptpB* | 0.70 |
| A0A0B6XM36 | Peptidase T | *pepT_1* | 0.70 |
| A0A090LUJ0 | Protease synthase and sporulation negative regulatory protein PAI 1 | *paiA* | 0.70 |
| A0A0D1H8Y0 | Glucose-6-phosphate isomerase | *pgi* | 0.70 |
| A0A077U2Q7 | Cell division protein ftsA | *ftsA* | 0.70 |
| A0A0D1JNT4 | ATP synthase subunit alpha | *atpA* | 0.70 |
| A0A0B6XRG6 | Teichoic acid export ATP-binding protein TagH | *tagH_2* | 0.70 |
| A0A077TUN9 | Siderophore staphylobactin ABC transporter%2C substrate-binding protein SirA | *sirA* | 0.70 |
| A0A077TXN4 | Ncharacterized conserved protein | *ERS140095_00443* | 0.70 |
| A0A0B6XQ25 | Aspartokinase | *lysC2* | 0.70 |
| A0A0D1JFQ2 | Glutathione peroxidase | *QU38_12880* | 0.71 |
| A0A0E8IKP1 | GMP synthase [glutamine-hydrolyzing] | *guaA* | 0.71 |
| A0A0D6FMN0 | Putative cytosolic protein | *ERS445051_00083* | 0.71 |
| A0A0D1H3K1 | Phosphoglucosamine mutase | *glmM* | 0.72 |
| A0A0D6WD10 | Acetyltransferase | *paiA* | 0.72 |
| A0A0D1GUW7 | GTPase Era | *era* | 0.72 |

| Accession Number | Protein Name | Gene Name | Fold Change |
|---|---|---|---|
| A0A090M224 | Transcription termination factor | *rho* | 0.72 |
| A0A0D1I3S9 | Outer membrane protein | *SAJPND1_01920* | 0.72 |
| A0A0D1EZE3 | Putative pyruvate, phosphate dikinase regulatory protein | *QU38_09115* | 0.72 |
| A0A090LQ50 | Isochorismate synthase family protein | *SAU060112_10293* | 0.73 |
| A0A0D1GMN8 | Hydrolase | *QU38_11475* | 0.73 |
| A0A0D1JNW2 | Strain SA-120 Contig624, whole genome shotgun sequence | - | 0.73 |
| A0A077UC86 | PTS family glucose/glucoside (Glc) porter component IIABC | *glcB* | 0.73 |
| A0A0C5HKH2 | PTS-dependent dihydroxyacetone kinase phosphotransfer protein | *dhaM* | 0.73 |
| A0A077U828 | Serine-protein kinase RsbW | *rsbW* | 0.73 |
| A0A0E8H010 | Cell shape-determining protein MreC | *ERS094548_00451* | 0.73 |
| A0A0D6HD63 | DNA repair protein Rad50 | *CH51_09905* | 0.73 |
| A0A0D1IP31 | Septation ring formation regulator EzrA | *ezrA* | 0.73 |
| A0A0B6XL97 | Periplasmic binding protein | *btuF* | 0.73 |
| A0A0B6XPA0 | Pyruvate kinase | *pykA* | 0.73 |
| A0A077U9J9 | Replicative DNA helicase | *dnaC_1* | 0.74 |
| A0A090LYW3 | Molybdopterin molybdenumtransferase | *moeA* | 0.74 |
| A0A0D1H3K1 | Phosphoglucosamine mutase | *glmM* | 0.75 |
| A0A068DT57 | Phosphoesterase%2C DHH family protein | *nrnA_1* | 0.75 |
| A0A0D6FKA0 | DNA gyrase subunit B | *gyrB* | 0.75 |
| A0A090LTA2 | PTS system N-acetylglucosamine-specific EIICB component | *nagP* | 0.75 |
| A0A0D6DEQ9 | Phosphoesterase, DHH family protein | *SAJPND1_01651* | 0.75 |
| A0A077UMA1 | Copper-translocating P-type ATPase | *copA* | 0.75 |
| A0A0B6XPB4 | Proline dipeptidase | *ERS094548_00400* | 0.75 |
| A0A0D1GSA9 | Strain SA-120 Contig630, whole genome shotgun sequence | - | 0.76 |
| A0A0D1HWI2 | tRNA-specific 2-thiouridylase MnmA | *mnmA* | 0.76 |
| A0A0D1I4D2 | Oligoendopeptidase F | *QU38_11615* | 0.77 |
| A0A0D1JNX0 | Glutamate racemase | *murI* | 0.77 |
| A0A090LUT3 | Uncharacterized protein | - | 0.77 |
| A0A090LR04 | Substrate binding domain of ABC-type glycine betaine transport system family protein | *SAU060112_10543* | 0.77 |
| A0A0D1HL09 | Redox-sensing transcriptional repressor Rex | *rex* | 0.78 |
| A0A0D6DM29 | 5-formyltetrahydrofolate cyclo-ligase | *SAJPND1_01504* | 0.78 |
| A0A077UYI3 | Ribonuclease 3 | *rnc* | 0.78 |
| A0A0D6DC64 | Cys-tRNA(Pro)/Cys-tRNA(Cys) deacylase | *SAJPND1_00699* | 0.78 |
| A0A0B6XN18 | Ribulokinase | *araB* | 0.78 |
| A0A077UHU0 | Membrane spanning protein | *ERS140147_01129* | 0.78 |
| A0A0D6GX81 | Cell wall surface anchor family protein | *ERS445052_00900* | 0.78 |
| A0A0D6DE64 | Putative cytosolic protein | *SAJPND1_01441* | 0.79 |
| A0A077W554 | Membrane associated protein | *ERS140026_02425* | 0.80 |
| A0A0D6DDB0 | Dihydrofolate reductase | *SAJPND1_01369* | 0.80 |
| A0A068A3K4 | Histidine kinase | *srrB* | 0.80 |

| Accession Number | Protein Name | Gene Name | Fold Change |
|---|---|---|---|
| A0A077UH28 | LytR family transcriptional regulator | *lytR_2* | 0.80 |
| A0A0D1JZ91 | General stress protein | *QU38_08215* | 0.80 |
| A0A090N275 | Transcriptional regulator, TetR family | *SAU060112_40385* | 0.80 |
| A0A0E1AFV2 | Non-canonical purine NTP pyrophosphatase | *SAZ172_1150* | 0.80 |
| A0A0B6XRB8 | Sodium export permease protein | *yhaP* | 0.80 |
| A0A077U2I8 | FMN-dependent NADH-azoreductase | *azoR* | 0.80 |
| A0A0C5HIW4 | Thiamine-phosphate synthase | *thiE* | 0.80 |
| A0A090M224 | Transcription termination factor | *rho* | 0.80 |
| A0A090LPK8 | DNA polymerase III PolC-type | *polC* | 0.80 |
| A0A0E1VK91 | Primosomal protein N' | *priA* | 0.80 |
| A0A077VMD2 | Trigger factor | *tig* | 0.80 |
| A0A077UEH0 | ATP synthase subunit a | *atpB* | 0.80 |
| A0A090LTA4 | Pyridine nucleotide-disulfide oxidoreductase family protein | *SAU060112_30030* | 0.80 |
| A0A0D1I3S9 | Outer membrane protein | *SAJPND1_01920* | 0.80 |
| A0A0D6DGB0 | Energy-coupling factor transporter ATP-binding protein EcfA | *ecfA* | 0.80 |
| A0A0B6XMX7 | DNA mismatch repair protein MutL | *mutL* | 0.80 |
| A0A068DXM4 | Magnesium transporter MgtE | *mgtE* | 0.81 |
| A0A077U5J7 | Ribonuclease Y | *rny* | 0.81 |
| A0A0B6XQ10 | Exported protein | *ERS094548_02603* | 0.82 |
| A0A077UNV6 | Pseudouridine synthase | *rluB* | 0.82 |
| A0A090LQ68 | Peptide chain release factor 3 | *prfC* | 0.82 |
| A0A0D1ICI0 | Pur operon repressor | *QU38_03120* | 0.82 |
| A0A0D6GRP2 | tRNA pseudouridine synthase B | *truB* | 0.82 |
| METN1 | Methionine import ATP-binding protein MetN 1 | *metN1* | 0.82 |
| A0A077UF55 | Purine nucleoside phosphorylase DeoD-type | *deoD_2* | 0.82 |
| A0A090LRA1 | Uncharacterized protein | - | 1.20 |
| A0A090LWG8 | Chaperone protein ClpB | *clpB* | 1.20 |
| A0A0D1GUG3 | Protein RecA | *recA* | 1.20 |
| A0A0D6DL16 | Virulence factor expression protein CvfB | *SAJPND1_01333* | 1.20 |
| A0A090N2E4 | Cof-like hydrolase | *SAU060112_60089* | 1.20 |
| A0A0C5HQR7 | Radical activating enzyme protein | *ERS445051_00968* | 1.20 |
| A0A0B6XT76 | Fructose-bisphosphate aldolase class 1 | *fda* | 1.20 |
| A0A0E0VN86 | Nitropropane dioxygenase | *ST398NM01_0904* | 1.20 |
| A0A0D1G285 | CDP-ribitol ribitolphosphotransferase | *QU38_14515* | 1.20 |
| A0A033UZD3 | Chaperone protein DnaK | *dnaK* | 1.21 |
| A0A090LW80 | CTP synthase | *pyrG* | 1.22 |
| A0A0D1HWG7 | Ribonuclease J | *QU38_12735* | 1.22 |
| A0A090LWD8 | Aspartyl/glutamyl-tRNA (Asn/Gln) amidotransferase subunit B | *gatB* | 1.22 |
| A0A0C5I0G0 | Aspartate aminotransferase | *aspC* | 1.22 |
| A0A090LQ19 | Bifunctional purine biosynthesis protein PurH | *purH* | 1.23 |
| A0A0E8HN36 | DNA topoisomerase 4 subunit B | *parE* | 1.23 |
| A0A069G7B5 | UPF0473 protein CO98_1354 | - | 1.25 |

| Accession Number | Protein Name | Gene Name | Fold Change |
|---|---|---|---|
| A0A0D1I3W4 | ATP-dependent Clp protease proteolytic subunit | *clpP* | 1.25 |
| A0A0D1I8Z9 | Mechanosensitive ion channel | *SAJPND1_00360* | 1.25 |
| A0A0B6XNU4 | DNA primase | *dnaG* | 1.25 |
| A0A090LQZ8 | Ribonucleoside-diphosphate reductase subunit beta | *nrdF* | 1.25 |
| A0A077VBV0 | Methionine aminopeptidase | *map_3* | 1.26 |
| A0A0D1H1Q3 | Glyoxalase family protein | *QU38_12370* | 1.26 |
| A0A069G7F5 | UPF0374 protein CO98_2312 | - | 1.26 |
| A0A0C2HEJ1 | Translation initiation factor IF-2 | *infB* | 1.27 |
| A0A090LTG7 | Acetyl-coenzyme A carboxylase carboxyl transferase subunit beta | *accD* | 1.27 |
| A0A0D1JLY3 | Strain SA-120 Contig625, whole genome shotgun sequence | - | 1.28 |
| A0A0D1I8U1 | 30S ribosomal protein S4 | - | 1.28 |
| A0A0D1H2U8 | 1,4-dihydroxy-2-naphthoyl-CoA synthase | *menB* | 1.28 |
| A0A0D1I4Y4 | Strain SA-120 Contig630, whole genome shotgun sequence | - | 1.29 |
| A0A0D1IJ21 | Peroxiredoxin | *QU38_04455* | 1.29 |
| A0A077ULL5 | LPXTG surface protein | *sdrD* | 1.30 |
| A0A0D1I5P8 | High-affinity zinc uptake system ATP-binding protein znuC | *SAJPND1_01510* | 1.30 |
| DRP35 | Lactonase drp35 | *drp35* | 1.30 |
| A0A0D6GCQ1 | Phosphoglycerate mutase | *gpmA_1* | 1.30 |
| A0A0E1AIF5 | 2-amino-3-ketobutyrate coenzyme A ligase | *kbl* | 1.30 |
| A0A0E1AJI0 | Ferredoxin-dependent glutamate synthase | *SAZ172_2563* | 1.30 |
| A0A0B6XMB1 | Branched-chain alpha-keto acid dehydrogenase subunit E2 | *pdhC* | 1.30 |
| A0A090N1T0 | Uncharacterized protein | - | 1.30 |
| A0A0E1VGD1 | Fumarate hydratase class II | *fumC* | 1.31 |
| SYI | Isoleucine-tRNA ligase | *ileS* | 1.32 |
| A0A0D1G603 | RNA polymerase sigma factor SigA | *sigA* | 1.32 |
| A0A0D1EUJ0 | Cytidylate kinase | *cmk* | 1.32 |
| A0A069FS69 | Phosphocarrier protein HPr | *ptsH* | 1.33 |
| A0A0D6DLY3 | Pyrroline-5-carboxylate reductase | *proC* | 1.33 |
| A0A0D1FPD0 | Elongation factor G | *fusA* | 1.33 |
| A0A0D1IQB7 | Enoyl-[acyl-carrier-protein] reductase [NADPH] | *QU38_11665* | 1.33 |
| A0A0D1K0Z5 | UDP-N-acetylglucosamine 1-carboxyvinyltransferase | *murA* | 1.34 |
| A0A0D1GZ06 | Putative septation protein SpoVG | *spoVG* | 1.35 |
| A0A068A8I1 | Serine/threonine protein kinase | *prkC* | 1.36 |
| A0A0D6DKR6 | Bifunctional protein FolD | *folD* | 1.36 |
| A0A077UEH9 | Thymidine kinase | *tdk* | 1.36 |
| A0A0D1GQ94 | Transcriptional regulator IcaR | *SAJPND1_02678* | 1.36 |
| A0A0D1I5C0 | Methionyl-tRNA formyltransferase | *fmt* | 1.36 |
| A0A0C5HRP6 | Peptidase family U32 | *yhbU_2* | 1.36 |
| A0A0D1JCM4 | 50S ribosomal protein L11 | - | 1.37 |
| A0A0E1AFG8 | Peptide deformylase | *def* | 1.38 |
| A0A0E0VNX4 | Ribosome-associated factor Y | *ST398NM01_0828* | 1.38 |

| Accession Number | Protein Name | Gene Name | Fold Change |
|---|---|---|---|
| A0A0C2L9V4 | Putative N-acetylmannosamine-6-phosphate 2-epimerase | *nanE* | 1.38 |
| A0A0D1JJZ3 | Central glycolytic protein regulator | *QU38_10935* | 1.39 |
| A0A0D1J8V1 | Glycerophosphoryl diester phosphodiesterase | *SAJPND1_01669* | 1.39 |
| A0A0D6DEH7 | Dimethylallyltransferase | *SAJPND1_01475* | 1.40 |
| A0A0D6HUF4 | Phosphoenolpyruvate-protein phosphotransferase | *ptsI* | 1.40 |
| A0A090LUA2 | Ribosome biogenesis GTPase A | *rbgA* | 1.40 |
| A0A090LW97 | EVE domain protein | *ERS094548_02511* | 1.40 |
| A0A0C5HQ19 | Acetyl-coenzyme A carboxylase carboxyl transferase subunit alpha | *accA* | 1.40 |
| A0A077USS5 | 2-C-methyl-D-erythritol 4-phosphate cytidylyltransferase | *ispD2* | 1.40 |
| A0A0D1H6R9 | Aconitate hydratase | *SAJPND1_01292* | 1.40 |
| A0A077USL8 | L-lactate permease | *lctP_1* | 1.41 |
| A0A0D1IKI9 | Strain SA-120 Contig619, whole genome shotgun sequence | - | 1.41 |
| A0A090LXW9 | Isocitrate dehydrogenase [NADP] | *icd* | 1.41 |
| D7RM11 | Translation elongation factor Tu (Fragment) | - | 1.42 |
| A0A0D1HD79 | tRNA binding domain protein | *SAJPND1_01690* | 1.42 |
| A0A090N220 | Cyclic pyranopterin monophosphate synthase accessory protein | *moaC* | 1.42 |
| A0A0D6DJ51 | 50S ribosomal protein L25 | *rplY* | 1.42 |
| A0A069FMB9 | DNA-directed RNA polymerase subunit alpha | *rpoA* | 1.43 |
| A0A077VNY2 | Teichoic acids export ATP-binding protein TagH | *tagH_1* | 1.44 |
| A0A0D1JI56 | Cell division initiation protein DivIVA | *QU38_12295* | 1.44 |
| A0A077TZY1 | Thioredoxin reductase | *trxB_2* | 1.45 |
| A0A090N127 | GTPase HflX | *hflX* | 1.46 |
| A0A077W129 | 30S ribosomal protein S13 | *rpsM* | 1.47 |
| A0A0D1I5C9 | Strain SA-120 Contig629, whole genome shotgun sequence | - | 1.47 |
| A0A0B6XQV4 | Alpha-amylase | *malA* | 1.48 |
| A0A0D1GT36 | RNA polymerase sigma factor | *SAJPND1_02037* | 1.48 |
| A0A0D6H5T9 | NAD-dependent protein deacetylase | *cobB* | 1.49 |
| A0A0D1H4E0 | Strain SA-120 Contig630, whole genome shotgun sequence | - | 1.49 |
| A0A0D1IHY2 | 50S ribosomal protein L29 | - | 1.50 |
| A0A090LU21 | Fe(3+)-citrate-binding protein YfmC | *yfmC* | 1.50 |
| A0A077U027 | 1-acyl-sn-glycerol-3-phosphate acyltransferase | *plsC* | 1.50 |
| A0A077UXQ0 | UTP-glucose-1-phosphate uridylyltransferase | *gtaB* | 1.50 |
| A0A0D1IPU6 | Adenylosuccinate synthetase | *purA* | 1.52 |
| A0A0D6DKC9 | UPF0738 protein SAJPND1_00926 | - | 1.52 |
| A0A0E1AI69 | Uncharacterized protein | - | 1.52 |
| A0A0D1I2B3 | Chaperone protein DnaJ | *dnaJ* | 1.53 |
| A0A090LRD1 | Molecular chaperone Hsp31 and glyoxalase 3 | *hchA* | 1.53 |
| A0A0D1JMR1 | Acyl carrier protein | *acpP* | 1.54 |
| A0A0B6XSS6 | Molybdate-binding protein | *modA* | 1.54 |
| A0A0D1K1G7 | Phosphopantetheine adenylyltransferase | *coaD* | 1.55 |
| A0A0C5I8L9 | Malonyl CoA-acyl carrier protein transacylase | *fabD* | 1.55 |

| Accession Number | Protein Name | Gene Name | Fold Change |
|---|---|---|---|
| A0A0E1APW1 | 1-pyrroline-5-carboxylate dehydrogenase | *rocA* | 1.55 |
| A0A0B6XPW5 | UDP-N-acetylmuramoylalanyl-D-glutamate-2%2C 6-diaminopimelate ligase | *ERS094548_02204* | 1.55 |
| A0A0D6DQ24 | tRNA modification GTPase MnmE | *mnmE* | 1.55 |
| A0A0D1I6P2 | Diaminopimelate decarboxylase | *lysA* | 1.56 |
| A0A0B6XQS8 | Valine-tRNA ligase | *valS* | 1.56 |
| A0A0D1FKQ9 | 30S ribosomal protein S9 | *rpsI* | 1.57 |
| A0A0D1JTQ6 | 50S ribosomal protein L15 | - | 1.58 |
| A0A0D1J4E7 | Glutamate dehydrogenase | *QU38_11405* | 1.58 |
| A0A0C5I0S0 | Mannitol-1-phosphate 5-dehydrogenase | *mtlD* | 1.59 |
| HUTG | Formimidoylglutamase | *hutG* | 1.60 |
| A0A0D1HT84 | 50S ribosomal protein L5 | *rplE* | 1.60 |
| A0A077UHD3 | Uridine kinase | *udk* | 1.60 |
| A0A0B6XP80 | Similar to oxidoreductase | *yhdN* | 1.60 |
| A0A077UEE8 | Proline--tRNA ligase | *proS* | 1.60 |
| A0A077VSG0 | 50S ribosomal protein L9 | *rplI* | 1.61 |
| A0A068DY65 | DNA ligase | *lig* | 1.61 |
| A0A0C2I3Y5 | Branched-chain amino acid aminotransferase | *ilvE* | 1.61 |
| A0A0B6XQ41 | GMP reductase | *guaC* | 1.61 |
| A0A0C5HY37 | Glycine cleavage system H protein | *gcvH_2* | 1.61 |
| A0A0D1GWI4 | 50S ribosomal protein L4 | *rplD* | 1.62 |
| A0A0D1I3V2 | UDP-N-acetylenolpyruvoylglucosamine reductase | *murB* | 1.62 |
| A0A0B6XL55 | Hydrolase (HAD superfamily) | *ywpJ_1* | 1.63 |
| A0A0D1HWC8 | 30S ribosomal protein S2 | *rpsB* | 1.63 |
| A0A069FME4 | 50S ribosomal protein L16 | *rplP* | 1.63 |
| A0A0D1HB86 | Strain SA-120 Contig627, whole genome shotgun sequence | - | 1.63 |
| A0A0D1HPJ7 | DNA-directed RNA polymerase subunit omega | *rpoZ* | 1.64 |
| A0A0D1I8Q0 | Tyrosine-tRNA ligase | *tyrS* | 1.64 |
| A0A033UZC9 | 30S ribosomal protein S21 | *rpsU* | 1.65 |
| A0A0C5I321 | Glutamate-1-semialdehyde 2,1-aminomutase | *gsaB* | 1.65 |
| A0A0D1FN85 | Methylenetetrahydrofolate-tRNA-(uracil-5-)-methyltransferase TrmFO | *gid* | 1.66 |
| A0A0D1HXC4 | Glycine-tRNA ligase | *glyQS* | 1.69 |
| A0A077VEQ5 | Endonuclease III | *nth* | 1.69 |
| A0A077TYV7 | 3-oxoacyl-[acyl-carrier-protein] synthase 2 | *fabF* | 1.70 |
| A0A0D6DDD4 | Phosphate acyltransferase | *plsX* | 1.70 |
| A0A077V1V2 | 50S ribosomal protein L10 | - | 1.70 |
| A0A0D1I5W0 | Phenylalanine-tRNA ligase alpha subunit | *pheS* | 1.70 |
| A0A0D1K2T0 | Putative cytosolic protein | *QU38_07185* | 1.71 |
| A0A0D1HMM2 | 30S ribosomal protein S3 | *rpsC* | 1.72 |
| A0A090LRJ4 | Ferrochelatase | *hemH* | 1.72 |
| A0A0D1JTN2 | 30S ribosomal protein S10 | - | 1.72 |

| Accession Number | Protein Name | Gene Name | Fold Change |
|---|---|---|---|
| A0A077UA73 | RNA binding protein%2C contains ribosomal protein S1 domain | *yugI_2* | 1.75 |
| A0A090M2G5 | Cobalamin biosynthesis CobT VWA domain protein | *SAU060112_70100* | 1.75 |
| A0A0D1H0K7 | 50S ribosomal protein L23 | *rplW* | 1.75 |
| A0A077U6Y9 | CobB/CobQ-like glutamine amidotransferase domain-containing protein | *ERS140026_02417* | 1.75 |
| A0A0D6DCH6 | Scaffold protein sufB | *SAJPND1_00832* | 1.75 |
| A0A033UV22 | Translation initiation factor IF-1 | *infA* | 1.75 |
| A0A0D1H7G8 | Thioredoxin | *QU38_12030* | 1.76 |
| A0A090LXL4 | Glutamate-tRNA ligase | *gltX* | 1.77 |
| A0A077U0Z6 | Biotin carboxyl carrier protein of acetyl-CoA carboxylase | *accB* | 1.78 |
| A0A0D1JM30 | Ribosomal silencing factor RsfS | *rsfS* | 1.82 |
| A0A0D1HMF1 | Exodeoxyribonuclease 7 small subunit | *xseB* | 1.83 |
| A0A0D1K1S3 | 50S ribosomal protein L2 | *rplB* | 1.83 |
| A0A0D1J193 | 30S ribosomal protein S12 | *rpsL* | 1.83 |
| A0A0D1HQY5 | Nucleoside diphosphate kinase | *ndk* | 1.83 |
| A0A0B6XQU1 | Molybdenum cofactor biosynthesis protein E | *moaE* | 1.84 |
| A0A077V9E3 | Acetyltransferase | *ERS140254_00101* | 1.85 |
| A0A0D1JTT1 | AspS protein | *aspS* | 1.85 |
| A0A0D1IBH8 | 30S ribosomal protein S5 | *rpsE* | 1.85 |
| A0A0D1JQF8 | NADH dehydrogenase | *QU38_11320* | 1.86 |
| A0A0D1H8Z3 | 3-oxoacyl-[acyl-carrier-protein] synthase 3 | *fabH* | 1.86 |
| A0A0B6XLK3 | Haloacid dehalogenase-like hydrolase | *ppaX* | 1.86 |
| A0A0D1JTP6 | Putative cytosolic protein | *QU38_03765* | 1.87 |
| A0A077VD35 | Multidrug resistance protein (Function not yet clear) | *ERS140254_01513* | 1.89 |
| A0A0D1H5H3 | Phenol-soluble modulin export ABC transporter ATP-binding protein PmtC | *QU38_06870* | 1.89 |
| A0A0D1J1L9 | Phosphoglycerate kinase | *pgk* | 1.90 |
| A0A077VKN4 | 50S ribosomal protein L1 | *rplA* | 1.91 |
| A0A0B6XPU4 | Ribosome maturation factor RimP | *rimP* | 1.94 |
| A0A0D1H088 | 50S ribosomal protein L35 | - | 1.94 |
| A0A0D1HPY4 | UDP-N-acetylmuramoyl-L-alanyl-D-glutamate-L-lysine ligase | *murE* | 1.94 |
| A0A0D1IUZ1 | Citrate synthase | *QU38_08465* | 1.95 |
| A0A077VJ87 | GTP-binding protein TypA/BipA | *typA* | 1.95 |
| A0A0D1JX26 | Glutamine synthetase | *glnA* | 1.95 |
| A0A090N1V7 | Porphobilinogen deaminase | *hemC* | 1.97 |
| A0A0D1IHK1 | 50S ribosomal protein L20 | *rplT* | 2.00 |
| A0A077UBW6 | O-methyltransferase family protein | *ERS140162_01570* | 2.00 |
| A0A077UAB5 | D-2-hydroxyacid dehydrogenase | *ldhD_2* | 2.00 |
| A0A0D1HF64 | 30S ribosomal protein S18 | *rpsR* | 2.00 |
| A0A0D1H5C2 | 50S ribosomal protein L22 | *rplV* | 2.00 |
| A0A090LY44 | Alanine-tRNA ligase | *alaS* | 2.00 |

| Accession Number | Protein Name | Gene Name | Fold Change |
|---|---|---|---|
| A0A0D1IJS9 | 50S ribosomal protein L21 | *rplU* | 2.00 |
| A0A0D6DK60 | Uncharacterized protein | - | 2.01 |
| A0A0B6XPZ7 | Dihydrolipoyllysine-residue succinyltransferase component of 2-oxoglutarate dehydrogenase complex | *odhB* | 2.01 |
| A0A0D1I3J5 | Urease accessory protein UreE | *ureE* | 2.05 |
| A0A033UV78 | 50S ribosomal protein L36 | *rpmJ* | 2.06 |
| A0A090LQD9 | Putative peptidyl-prolyl cis-trans isomerase | *SAU060112_10378* | 2.06 |
| A0A068DWZ8 | Ribosomal protein L11 methyltransferase | *prmA* | 2.06 |
| A0A0D1HYX5 | 50S ribosomal protein L14 | *rplN* | 2.06 |
| A0A090M065 | Serine-tRNA ligase | *serS* | 2.06 |
| A0A0B6XKV9 | Isochorismatase | *yecD* | 2.08 |
| A0A090N2E5 | Mannitol-specific phosphotransferase enzyme IIA component | *mtlF* | 2.08 |
| A0A0D6DNQ3 | Transcription accessory protein | *SAJPND1_02036* | 2.08 |
| A0A090LYX3 | Inosine-uridine preferring nucleoside hydrolase | *SAU060112_40119* | 2.09 |
| A0A0B6XQV6 | Iron-sulfur cluster assembly/repair protein ApbC | *apbC* | 2.09 |
| A0A0D1HPY4 | UDP-N-acetylmuramoyl-L-alanyl-D-glutamate-L-lysine ligase | *murE* | 2.10 |
| A0A0B6XMV1 | Proline/betaine transporter | *proP* | 2.10 |
| A0A090LUB5 | Uncharacterized protein | - | 2.10 |
| A0A0D1FKP4 | 50S ribosomal protein L30 | *rpmD* | 2.11 |
| A0A077UEX9 | Putative cysteine ligase BshC | *bshC* | 2.13 |
| A0A0D1K117 | Peptide methionine sulfoxide reductase MsrA | *msrA* | 2.14 |
| A0A0D1IZY7 | Superoxide dismutase | *QU38_15115* | 2.15 |
| A0A090LUF7 | DNA repair/chromosome segregation ATPase | *ERS445051_02217* | 2.17 |
| A0A0D1IW07 | Histidine-tRNA ligase | *hisS* | 2.18 |
| A0A0D6WDW6 | Major tail protein | *ERS445051_01394* | 2.20 |
| A0A0D1IZ12 | D-alanine-poly(phosphoribitol) ligase subunit 2 | *dltC* | 2.22 |
| A0A0D1J2L0 | Phosphomethylpyrimidine kinase | *SAJPND1_00574* | 2.25 |
| A0A090LTM2 | 5'-methylthioadenosine/S-adenosylhomocysteine nucleosidase | *pfs* | 2.28 |
| A0A090LPS7 | Putative ribosomal protein YlxQ | *rplGA* | 2.29 |
| A0A0D1H3G2 | 50S ribosomal protein L31 type B | *rpmE2* | 2.30 |
| A0A0D1HTN7 | 50S ribosomal protein L18 | *rplR* | 2.31 |
| A0A077UL50 | ATP-dependent Clp protease ATP-binding subunit ClpX | *clpX* | 2.39 |
| A0A0D6DD40 | UPF0223 protein SAJPND1_01024 | - | 2.40 |
| A0A0D6DE20 | Alanine dehydrogenase | *SAJPND1_01657* | 2.42 |
| A0A0D1IX47 | Trp repressor binding protein | *SAJPND1_00599* | 2.45 |
| A0A0D1HG99 | Strain SA-120 Contig630, whole genome shotgun sequence | - | 2.47 |
| A0A0D1GXI2 | Strain SA-120 Contig624, whole genome shotgun sequence | - | 2.47 |
| A0A0D1JWV4 | 50S ribosomal protein L28 | *rpmB* | 2.55 |
| A0A077W130 | Iron-sulfur cluster assembly ATPase SufC | *yurY* | 2.55 |
| A0A077UKA9 | 50S ribosomal protein L24 | *rplX* | 2.55 |
| A0A0D1I822 | 30S ribosomal protein S20 | *rpsT* | 2.56 |
| A0A0D1JQL5 | Thioredoxin | *SAJPND1_00815* | 2.56 |

| Accession Number | Protein Name | Gene Name | Fold Change |
|---|---|---|---|
| A0A0C5IA88 | Formate-tetrahydrofolate ligase | *fhs* | 2.57 |
| A0A0E1VP50 | Aldehyde dehydrogenase (NAD) family protein | *HMPREF0776_0903* | 2.62 |
| A0A033V5W3 | Cold shock protein CspA | *cspA* | 2.63 |
| A0A0B6XR80 | Glyoxylate reductase/Glyoxylate reductase/Hydroxypyruvate reductase | *SASCBU26_02308* | 2.63 |
| A0A0D1J2E1 | Translation initiation factor IF-3 | *infC* | 2.64 |
| A0A0E1VTG8 | Oxidoreductase, FAD/FMN-binding protein | *HMPREF0776_1945* | 2.65 |
| A0A0D3Q486 | Uncharacterized protein | - | 2.69 |
| A0A0B6XKR5 | Capsular polysaccharide synthesis enzyme Cap5I | *capI* | 2.70 |
| A0A090LYE2 | Aminomethyltransferase | *gcvT* | 2.72 |
| A0A077VL65 | Succinyl-diaminopimelate desuccinylase | *dapE* | 2.75 |
| A0A0B6XSJ7 | Alcohol dehydrogenase | *curA* | 2.76 |
| SYL | Leucine-tRNA ligase | *leuS* | 2.77 |
| A0A090M2S9 | Uncharacterized deoxyribonuclease YabD | - | 2.77 |
| A0A0D1JCN9 | Peptidyl-tRNA hydrolase | *pth* | 2.85 |
| A0A0B6XST7 | Urease subunit alpha | *ureC* | 3.00 |
| A0A0E1X668 | 2-oxoglutarate ferredoxin oxidoreductase subunit beta | *HMPREF0769_12488* | 3.00 |
| MURC | UDP-N-acetylmuramate-L-alanine ligase | *murC* | 3.00 |
| A0A0D1GP04 | Succinyl-CoA ligase [ADP-forming] subunit alpha | *SAJPND1_01177* | 3.05 |
| A0A090LT64 | Uncharacterized protein | - | 3.09 |
| A0A0E1X586 | Phage major tail protein | *HMPREF0769_12240* | 3.15 |
| A0A0D6DD94 | Phosphoesterase | *SAJPND1_01079* | 3.15 |
| A0A0D1JZ55 | Putative cytosolic protein | *SAJPND1_01294* | 3.25 |
| A0A0D1HIZ5 | Putative cytosolic protein | *SAJPND1_01624* | 3.29 |
| A0A0D6GZ63 | Phage protein | *ERS445051_01398* | 3.35 |
| A0A0D1IPY9 | Organic hydroperoxide resistance protein | *SAJPND1_00812* | 3.44 |
| A0A0D6G3W7 | Serine-aspartate repeat-containing protein E | *sdrE* | 3.47 |
| A0A0D1H5K7 | Strain SA-120 Contig628, whole genome shotgun sequence | - | 3.47 |
| A0A0B6XKZ1 | Sorbitol dehydrogenase | *gutB_1* | 3.60 |
| A0A0D1JVV7 | Oxygen-dependent choline dehydrogenase | *betA* | 3.60 |
| A0A033V401 | Ferredoxin | *V070_00532* | 3.65 |
| A0A090LXA3 | Lipase/esterase LipA | *SAU060112_10612* | 3.70 |
| A0A0B6XLS6 | UDP-2-acetamido-2%2C6-dideoxy-beta-L-talose 4-dehydrogenase | *ERS094548_01225* | 3.80 |
| A0A077UAB9 | S-ribosylhomocysteine lyase | *luxS* | 3.80 |
| A0A0D6GUT4 | Membrane protein | *mmpL8* | 3.85 |
| A0A090M0E8 | Molybdopterin synthase sulfur carrier subunit | *moaD* | 3.91 |
| A0A0D6HBG6 | UPF0435 protein ERS445051_01856 | - | 3.93 |
| A0A0E1VYJ0 | Urease accessory protein UreG | *ureG* | 3.95 |
| A0A069G4G3 | Recombination protein RecR | *recR* | 4.00 |
| G0XY48 | Clumping factor A | *clfA* | 4.05 |
| A0A077UT21 | Phage protein | *ERS140159_01807* | 4.15 |

| **Accession Number** | **Protein Name** | **Gene Name** | **Fold Change** |
|---|---|---|---|
| A0A0D6HFI7 | Succinyl-diaminopimelate desuccinylase | *dapE* | 4.18 |
| A0A0D6DN74 | Aldehyde dehydrogenase | *SAJPND1_02098* | 4.30 |
| A0A0D1KAK6 | Rhodanese-related sulfurtransferase | *SAJPND1_01707* | 4.40 |
| A0A0D1HB94 | 2,3-bisphosphoglycerate-independent phosphoglycerate mutase | *gpmI* | 4.45 |
| A0A0D1GFP6 | Transcription elongation factor GreA | *greA* | 4.50 |
| A0A090N1L9 | UDP-glucose 4-epimerase | *capD* | 4.65 |
| A0A090LXH5 | 3-hexulose-6-phosphate isomerase | *hxlB* | 4.77 |
| A0A0B4N811 | 3-hexulose-6-phosphate synthase | *CH51_02975* | 4.90 |
| A0A090LZV4 | Betaine-aldehyde dehydrogenase | *betB* | 5.30 |
| A0A0D1IUM8 | L-serine dehydratase beta subunit | *SAJPND1_02531* | 5.63 |
| A0A077UW75 | YceI-like domain protein | *ERS140266_00175* | 5.70 |
| A0A0C5HEJ3 | Endoribonuclease L-PSP | *yabJ* | 5.75 |
| A0A0D1FVH2 | Strain SA-120 Contig630, whole genome shotgun sequence | - | 6.06 |
| A0A0D1HWG4 | ProteaseI | *SAJPND1_01839* | 6.27 |
| A0A0B6XR64 | Urease subunit beta | *ureB* | 6.58 |
| A0A0B6XQZ3 | S-ribosylhomocysteine lyase | *luxS* | 6.75 |
| A0A077U0G6 | Glycine betaine transporter OpuD | *opuD_1* | 7.61 |
| A0A0D1H3E1 | Sced | *SAJPND1_02072* | 8.25 |
| A0A0D1FGD6 | Strain SA-120 Contig629, whole genome shotgun sequence | - | 8.92 |
| A0A077UNT4 | YozC | *ERS140026_00525* | 9.20 |
