# Supplementary material for: iTRAQ-Based Quantitative Proteomic Profiling of Staphylococcus aureus Under Different Osmotic Stress Conditions
Source: Front Microbiol. 2019 May 29;10:1082. doi: 10.3389/fmicb.2019.01082 (PMC6549500; doi:10.3389/fmicb.2019.01082)
Supplement: Supplementary file 6 [file Data_Sheet_6.PDF]

Table S6 Differentially expressed proteins in the 20% NaCl group compared with the 10% NaCl group. The DEPs in Figure 5 with fold change ≥2 or ≤0.5 are labelled in red.

**Accession Number**	**Protein Name**	**Gene Name**	**Fold Change**
A0A090LVJ3	Ornithine carbamoyltransferase	*arcB*	0.14
A0A0D1INB5	Arginine deiminase	*arcA*	0.16
A0A0D6GYJ2	Alanine dehydrogenase	*ald1*	0.18
A0A0D1HYC8	Nitrite reductase [NAD(P)H] large subunit	*SAJPND1_02388*	0.19
A0A0D6GYJ2	Alanine dehydrogenase	*ald1*	0.21
A0A077VDA6	Lipoprotein	*metQ_1*	0.22
A0A077U1D0	Amino acid permease	*steT*	0.23
A0A0D1HYD3	Strain SA-120 Contig626, whole genome shotgun sequence	-	0.23
A0A090LQ06	Uncharacterized protein	-	0.23
A0A0D6DA07	Iron-sulfur cluster repair protein ScdA	*scdA*	0.24
A0A0B6XMG3	Argininosuccinate synthase	*argG*	0.25
A0A090LWI3	Argininosuccinate lyase	*argH*	0.25
T1Y8N9	Ornithine carbamoyltransferase	*SAKOR_01090*	0.26
A0A0E1VRD5	Threonine ammonia-lyase	*ilvA*	0.26
A0A0D1K3I9	Cold shock protein	*QU38_11035*	0.28
A0A0D1GZV1	Pyruvate dehydrogenase	*SAJPND1_02539*	0.28
A0A0D6DIM1	Ribokinase	*SAJPND1_00306*	0.29
A0A068A921	Nitrate reductase	*narH*	0.30
A0A0D1JKL8	Carbamate kinase	*QU38_12170*	0.30
A0A0E1VKR4	ABC transporter, ATP-binding protein	*HMPREF0776_1978*	0.32
OMP7	77 kDa membrane protein	*SACOL2002*	0.33
A0A0B6XPE8	Pyruvate carboxylase	*cfiB_1*	0.33
A0A090LQD2	Glycerophosphoryl diester phosphodiesterase	*glpQ*	0.36
A0A077U2I8	FMN-dependent NADH-azoreductase	*azoR*	0.38
A0A0E1VQ76	Biotin synthase	*bioB*	0.38
A0A0B6XNX0	Alanine dehydrogenase	*ald2_1*	0.39
A0A0E7DTW4	Putative lipoprotein	*ERS157365_00280*	0.39
Q4W8K6	3-ketoacyl-CoA thiolase	*thlA*	0.39
A0A0D6FMN0	Putative cytosolic protein	*ERS445051_00083*	0.40
A0A0B6XPL1	Aldo/keto reductase family protein	*yvgN*	0.41
A0A0D1HUW5	Threonine synthase	*SAJPND1_01270*	0.41
A0A077ULW3	Transcriptional regulator	*immR*	0.41
A0A0D6DE64	Putative cytosolic protein	*SAJPND1_01441*	0.42
A0A090N157	Alpha-hemolysin	*hly*	0.42
A0A0D1H6Z1	Nitric oxide synthase oxygenase	*QU38_06960*	0.43
A0A0D1I4N9	Strain SA-120 Contig630, whole genome shotgun sequence	-	0.44
A0A090LYP3	Protein EsaA	*esaA*	0.44

Accession Number	Protein Name	Gene Name	Fold Change
A0A0D1H4E0	Strain SA-120 Contig630, whole genome shotgun sequence	-	0.44
A0A0B6XP33	Oligopeptide transport ATP-binding protein oppD	*oppD_1*	0.44
A0A090LSG7	L-lactate dehydrogenase	*ldhA*	0.45
A0A0C5HMT4	GntR family transcriptional regulator	*treR_1*	0.45
A0A077U0D7	Protein of pXO2-46	*ERS140095_00709*	0.45
A0A0D6DDK7	Uncharacterized protein	-	0.46
A0A0D6FNA4	ATPase	*CH51_00375*	0.46
A0A0D6DDT0	Homoserine dehydrogenase	*SAJPND1_01269*	0.46
A0A0E1XJY5	Abi-like protein	*HMPREF0769_10093*	0.46
A0A077UAB5	D-2-hydroxyacid dehydrogenase	*ldhD_2*	0.47
A0A0D1G5I0	Strain SA-120 Contig630, whole genome shotgun sequence	-	0.47
A0A0D6FS99	Putative polyribitolphosphotransferase	*tagF_1*	0.47
A0A0D1JQX0	Strain SA-120 Contig628, whole genome shotgun sequence	-	0.47
A0A077USG2	Immunodominant staphylococcal antigen A	*isaA*	0.47
A0A0D1K2N9	GlnQ protein	*glnQ*	0.47
A0A0E1VGA3	Uncharacterized protein	-	0.48
A0A0D6HWL4	ATP-dependent helicase/deoxyribonuclease subunit B	*addB*	0.49
A0A090LVM4	Poly-beta-1,6-N-acetyl-D-glucosamine N-deacetylase	*icaB*	0.49
A0A0D6HVT0	ATP-dependent helicase/nuclease subunit A	*addA*	0.49
SBI	Immunoglobulin-binding protein sbi	*sbi*	0.50
A0A0D6DFD4	Beta-lactamase regulator protein	*SAJPND1_01896*	0.50
A0A0C5HVE0	Pyruvate decarboxylase%3B Alpha-keto-acid decarboxylase	*ipdC*	0.50
A0A0D1I054	L-lactate dehydrogenase	*ldh*	0.50
ADH	Alcohol dehydrogenase	*adh*	0.50
A0A090LW57	ABC transporter, permease protein	*SAU060112_100003*	0.50
A0A077V172	Respiratory nitrate reductase gamma chain	*narI*	0.50
A0A0D6GIM9	Autolysin	*atl_1*	0.50
A0A077TYC4	Similar to putative sodium/glucose cotransporter	*sglT*	0.50
A0A077U2Q8	Quinol oxidase polypeptide I QoxB	*qoxB*	0.50
A0A090LVS1	Potassium-transporting ATPase B chain	*kdpB*	0.50
A0A077U7R0	3-isopropylmalate dehydratase large subunit	*leuC*	0.50
A0A0D6DHW6	Glycosyltransferase	*SAJPND1_00237*	0.50
A0A090M1C4	Dihydroorotate dehydrogenase (quinone)	*pyrD*	0.51
A0A0D1IXJ7	Gamma-hemolysin B subunit HlgB	*SAJPND1_02412*	0.52
A0A0C5HKH2	PTS-dependent dihydroxyacetone kinase phosphotransfer protein	*dhaM*	0.52
A0A090N225	Secretory antigen	*ssaA*	0.53
A0A0D6D9X8	Glutaryl-CoA dehydrogenase	*SAJPND1_00208*	0.53

Accession Number	Protein Name	Gene Name	Fold Change
A0A0B6XPT4	D-3-phosphoglycerate dehydrogenase	*serA*	0.54
A0A077UPJ4	Phosphopentomutase	*deoB*	0.54
A0A0B6XPW9	Sodium/proline symporter	*putP*	0.54
A0A0B6XLS6	UDP-2-acetamido-2%2C6-dideoxy-beta-L-talose 4-dehydrogenase	*ERS094548_01225*	0.55
A0A0C5IA17	DNA-3-methyladenine glycosylase	*tag*	0.55
A0A0D1HTG9	Acetyltransferase, GNAT family	*QU38_10310*	0.55
A0A0D6DAW8	Long-chain-fatty-acid-CoA ligase	*SAJPND1_00209*	0.55
A0A0B6XTB4	N-acetylmuramoyl-L-alanine amidase%2C family 4	*ERS094548_01923*	0.55
A0A069FZ92	Branched-chain-amino-acid aminotransferase	*ilvE*	0.55
A0A0D1G1J9	Cytochrome c oxidase polypeptide II	*SAJPND1_00987*	0.56
A0A0D1GTD3	Strain SA-120 Contig622, whole genome shotgun sequence	-	0.56
A0A077USJ8	Putative lipoprotein	*ERS140159_00666*	0.56
A0A0B6XLU3	DNA-binding protein	*ERS094548_00209*	0.56
A0A0D1GVJ7	Poly (Glycerol-phosphate) alpha-glucosyltransferase	*SAJPND1_00558*	0.56
A0A0C6DRW9	Staphylococcus aureus DNA, S. aureus pathogenicity island, SaPI2R, complete sequence, strain: OC3	-	0.56
A0A077V1P6	Methionine ABC transporter ATP-binding protein	*ybbL*	0.56
A0A0E1AE46	Phosphoenolpyruvate-dihydroxyacetone phosphotransferase, dihydroxyacetone binding subunit DhaK	*SAZ172_0661*	0.57
A0A0D1JXR9	Strain SA-120 Contig630, whole genome shotgun sequence	-	0.57
A0A090LWR0	Phosphoglycerate mutase	*gpmA_1*	0.57
A0A0D1GU53	Uncharacterized protein	-	0.58
A0A0D6HMD3	General stress protein 26	*ydaG*	0.58
A0A0D1GC10	Strain SA-120 Contig629, whole genome shotgun sequence	-	0.58
A0A0D1HX32	Strain SA-120 Contig626, whole genome shotgun sequence	-	0.58
A0A077V5S2	Carbamate kinase	*arcC*	0.58
A0A0D1FZV0	Strain SA-120 Contig626, whole genome shotgun sequence	-	0.58
A0A0D1HN41	Phosphoenolpyruvate carboxykinase [ATP]	*pckA*	0.58
A0A077TYI6	Uncharacterized protein	-	0.58
A0A0D6DDB0	Dihydrofolate reductase	*SAJPND1_01369*	0.59
A0A0D1H334	Putative kinase associated protein B family protein	*QU38_11380*	0.59
A0A0D1G9T6	Tautomerase	*QU38_13190*	0.59
A0A0E1AIF3	Branched-chain alpha-keto acid dehydrogenase, E1 component, beta subunit	*bfmBAB*	0.59
A0A090M2J3	Uncharacterized protein	-	0.59

Accession Number	Protein Name	Gene Name	Fold Change
A0A068W8S1	Ribosomal RNA large subunit methyltransferase H	*orfX*	0.60
A0A090LVY0	Aspartate carbamoyltransferase	*pyrB*	0.60
EBH	Extracellular matrix-binding protein ebh	*ebh*	0.60
A0A090LW12	Poly (Glycerol-phosphate) alpha-glucosyltransferase	*SAU060112_10705*	0.60
A0A0D1IN71	Strain SA-120 Contig623, whole genome shotgun sequence	-	0.60
A0A0D6GUT9	Dehydrosqualene desaturase	*crtN*	0.60
A0A0B6XKT1	Aldehyde-alcohol dehydrogenase	*adhE*	0.60
A0A0B6XMC2	Lipoprotein	*metQ_2*	0.61
A0A0D6H8Z6	Thiaminase II	*tenA*	0.61
A0A0D1JLL3	Adenosylmethionine-8-amino-7-oxononanoate aminotransferase	*bioA*	0.61
A0A077UNV8	Glycosyltransferase	*pimB*	0.62
A0A0D6DGW3	Gamma-hemolysin A subunit HlgA	*SAJPND1_02409*	0.62
A0A077V9M9	Response regulator SaeR	*saeR*	0.62
A0A090N1L9	UDP-glucose 4-epimerase	*capD*	0.62
A0A0D1GBS9	Signal transduction protein TRAP	*SAJPND1_01796*	0.63
A0A090N1M7	Type-1 restriction enzyme R protein	*hsdR*	0.63
A0A0B6XMX7	DNA mismatch repair protein MutL	*mutL*	0.63
A0A0D3Q462	Uncharacterised protein	-	0.63
A0A077UNT2	Xpac	*ERS140159_00099*	0.63
A0A090LXY5	Uncharacterized protein	-	0.63
A0A0D1HXA7	D-alanine aminotransferase	*SAJPND1_01699*	0.63
A0A0D6DD31	4-hydroxy-tetrahydrodipicolinate synthase	*dapA*	0.63
A0A0B6XRK6	Nicotinate phosphoribosyltransferase	*ERS094548_02369*	0.63
A0A077W1K8	Pyrrolidone-carboxylate peptidase	*pcp*	0.64
A0A0D1FBM5	6-phosphogluconolactonase	*SAJPND1_01893*	0.64
A0A0D1GS08	GTP-sensing transcriptional pleiotropic repressor CodY	*codY*	0.64
A0A0D1HQ98	Histidine kinase	*QU38_10565*	0.64
A0A0E0VM51	Acetate CoA-transferase YdiF	*ST398NM01_0245*	0.64
A0A0D6DNS3	UDP-N-acetylmuramoyl-tripeptide-D-alanyl-D-alanine ligase	*SAJPND1_02056*	0.64
A0A0D1JT33	Cardiolipin synthase	*QU38_06435*	0.65
A0A0D1K2T0	Putative cytosolic protein	*QU38_07185*	0.65
A0A0D1IBW0	Strain SA-120 Contig620, whole genome shotgun sequence	-	0.65
A0A033V5W3	Cold shock protein CspA	*V070_00472*	0.65
A0A0B6XPW1	o-succinylbenzoate synthase	*menC*	0.65
A0A090N2F1	Lysine-specific permease	*lysP*	0.65
A0A077UH70	DNA/RNA helicase of DEAD/DEAH box family	*srmB*	0.65
A0A090LXB7	Luciferase-like monooxygenase	*SAU060112_20273*	0.66

Accession Number	Protein Name	Gene Name	Fold Change
A0A0D6HV17	Pyruvate carboxylase	*cfiB_2*	0.66
A0A077U8C2	ATP synthase subunit beta	*atpD*	0.66
A0A0B6XNE5	Hydrolase (HAD superfamily)	*ERS094548_00694*	0.66
A0A0B6XMB8	Uncharacterized protein	-	0.67
A0A0D1K8W2	Bifunctional protein PyrR	*pyrR*	0.67
A0A090LUG8	Drug resistance MFS transporter, drug: H+ antiporter-2 family	*SAU060112_40187*	0.67
A0A0D1JGI6	2-C-methyl-D-erythritol 4-phosphate cytidylyltransferase	*ispD*	0.67
A0A090LR04	Substrate binding domain of ABC-type glycine betaine transport system family protein	*SAU060112_10543*	0.67
A0A077VAK0	Hexose phosphate transport protein	*uhpT*	0.67
A0A0C2HZY8	Pyrimidine-nucleoside phosphorylase	*pdp*	0.67
A0A0D1GJW4	Arsenate reductase family protein	*QU38_11110*	0.67
A0A0D1HUP1	Nucleoside transporter	*QU38_09615*	0.67
A0A090M057	Putative ATP-dependent helicase DinG homolog	*dinG*	0.67
A0A0B6XRB8	Sodium export permease protein	*yhaP*	0.67
A0A090LR26	Uncharacterized oxidoreductase YcsN	-	0.68
A0A0D1KA16	Strain SA-120 Contig620, whole genome shotgun sequence	-	0.68
A0A0D1JMR1	Acyl carrier protein	*acpP*	0.68
A0A069FXC5	Alkyl hydroperoxide reductase, F subunit	*ahpF*	0.68
A0A077TUN9	Siderophore staphylobactin ABC transporter%2C substrate-binding protein SirA	*sirA*	0.70
A0A077V7K3	ABC transporter substrate-binding protein	*psaA*	0.70
A0A077UP25	Membrane-bound serine protease	*ERS140159_00273*	0.71
A0A090LQ50	Isochorismate synthase family protein	*SAU060112_10293*	0.71
A0A0D1H1Q3	Glyoxalase family protein	*QU38_12370*	0.71
A0A0D6DPK7	Staphyloxanthin biosynthesis protein CrtP	*SAJPND1_02566*	0.71
A0A077TYE7	Uncharacterized protein conserved in bacteria	-	0.71
A0A0B6XQV4	Alpha-amylase	*malA*	0.71
A0A090LUF4	Ribose-5-phosphate isomerase A	*rpiA*	0.72
A0A0D6GTK5	3-hydroxy-3-methylglutaryl coenzyme A reductase	*mvaA*	0.72
A0A090LVR9	Uncharacterized protein	-	0.72
A0A0D1JZ55	Putative cytosolic protein	*SAJPND1_01294*	0.72
A0A0B6XNT6	Bifunctional ligase/repressor BirA	*birA*	0.72
A0A0D6DD85	DNA Polymerase X family	*SAJPND1_01069*	0.72
A0A090LUQ0	Glycerate kinase	*glxK*	0.73
A0A0D1H8Y0	Glucose-6-phosphate isomerase	*pgi*	0.73
A0A0B6XLD2	Sporulation protein	*yaaT*	0.74
A0A077UDM1	Manganese ABC transporter%2C ATP-binding protein SitB	*sitB*	0.74

Accession Number	Protein Name	Gene Name	Fold Change
A0A077TZL7	Modification methylase MboII	*mboIIM*	0.74
A0A0D1EZR2	Strain SA-120 Contig627, whole genome shotgun sequence	-	0.74
A0A0E0VQZ9	Aldehyde dehydrogenase	*ST398NM01_2164*	0.74
A0A0D6HMU5	FmhA protein of FemAB family	*fmhA*	0.74
A0A077UUV1	Hydrogen peroxide-inducible genes activator	*oxyR*	0.74
A0A0D1IP51	Strain SA-120 Contig628, whole genome shotgun sequence	-	0.74
A0A0D3Q7P5	3-phosphoshikimate 1-carboxyvinyltransferase	*aroA_2*	0.75
A0A077UDD9	3-ketoacyl-CoA thiolase%3B Acetyl-CoA acetyltransferase	*ERS140248_02364*	0.75
A0A077VSV4	Peptide ABC transporter permease	*oppB_1*	0.75
Q9S2Z4	Cell surface protein map-w	*map-w*	0.75
A0A0D6DPS6	Putative membrane spanning protein	*SAJPND1_02654*	0.75
A0A0E1X586	Phage major tail protein	*HMPREF0769_12240*	0.75
A0A069FW70	Fructose-1,6-bisphosphatase class 3	*fbp*	0.75
A0A077ULL5	LPXTG surface protein	*sdrD*	0.76
A0A0C2HDA0	Glycerol-3-phosphate dehydrogenase	*glpD*	0.76
A0A0E1AHK5	Acetylornithine deacetylase/Succinyl-diaminopimelate desuccinylase-related deacylase	*SAZ172_1765*	0.77
A0A0D1J368	ATP synthase subunit b	*atpF*	0.77
A0A0D1IUP6	Strain SA-120 Contig628, whole genome shotgun sequence	-	0.77
A0A0D1JNT4	ATP synthase subunit alpha	*atpA*	0.78
A0A077UBW6	O-methyltransferase family protein	*ERS140162_01570*	0.78
A0A0D1GSA9	Strain SA-120 Contig630, whole genome shotgun sequence	-	0.78
A0A0B6XKT2	Ribose operon repressor%2C putative	*degA*	0.78
A0A0D1ICI0	Pur operon repressor	*QU38_03120*	0.79
A0A0D1I3S9	Outer membrane protein	*SAJPND1_01920*	0.80
A0A090M0I8	ATP synthase gamma chain	*atpG*	0.80
A0A0D1HWP8	Dephospho-CoA kinase	*coaE*	0.80
A0A0D6DEI5	Ribonuclease BN	*SAJPND1_01847*	0.80
A0A0D1FJV3	Glucose-6-phosphate 1-dehydrogenase	*zwf*	0.82
A0A0D1INU4	4-diphosphocytidyl-2-C-methyl-D-erythritol kinase	*ipk*	0.82
A0A0B6XLZ5	EDD%2C DegV family domain protein	*ERS094548_00154*	0.82
A0A068DT57	Phosphoesterase%2C DHH family protein	*nrnA_1*	0.82
A0A0E1VTG8	Oxidoreductase, FAD/FMN-binding protein	*HMPREF0776_1945*	0.83
A0A0D1H2F2	Pyruvate dehydrogenase E1 component beta subunit	*QU38_05725*	0.83
A0A0D6HD63	DNA repair protein Rad50	*CH51_09905*	0.83
A0A090M2D6	Oligoendopeptidase F	*SAU060112_70075*	0.83
A0A0D1I5J3	Ribulose-phosphate 3-epimerase	*QU38_12445*	0.83

Accession Number	Protein Name	Gene Name	Fold Change
A0A0D1J363	Uracil phosphoribosyltransferase	*upp*	0.83
A0A077UK70	Thymidylate synthase	*thyA*	0.83
A0A069FME4	50S ribosomal protein L16	-	1.20
A0A0E1AE25	Phosphoglycolate phosphatase	*SAZ172_0574*	1.20
A0A0D1JNI2	Strain SA-120 Contig622, whole genome shotgun sequence	-	1.21
A0A0D6DFF7	Uncharacterized protein	-	1.21
A0A077W129	30S ribosomal protein S13	*rpsM*	1.22
A0A0C5I0S0	Mannitol-1-phosphate 5-dehydrogenase	*mtlD*	1.22
A0A0D1IPY9	Organic hydroperoxide resistance protein	*SAJPND1_00812*	1.22
A0A0C2HEJ1	Translation initiation factor IF-2	*infB*	1.22
A0A0D1JTQ6	50S ribosomal protein L15	-	1.23
A0A0D1FN85	Methylenetetrahydrofolate-tRNA-(uracil-5-)-methyltransferase TrmFO	*gid*	1.23
A0A090LZA4	Chorismate mutase I/2-keto-3-deoxy-D-arabino-heptulosonate-7-phosphate synthase I beta%2C AroH/AroA I beta	*aroA*	1.24
A0A090LZZ7	Uncharacterized peptidase YqhT	-	1.24
A0A090LQZ8	Ribonucleoside-diphosphate reductase subunit beta	*nrdF*	1.25
A0A0C5I2R4	Peptidase propeptide and YPEB domain-containing protein	*ERS445051_01691*	1.25
A0A0C5I321	Glutamate-1-semialdehyde 2,1-aminomutase	*gsaB*	1.25
A0A077U6M2	3-oxoacyl-[acyl-carrier protein] reductase	*fabG_1*	1.26
A0A0B6XQS8	Valine-tRNA ligase	*valS*	1.26
A0A0D1K1S3	50S ribosomal protein L2	-	1.27
A0A0C5HHM0	Octanoyltransferase LipM	*lipM*	1.27
A0A077V1V2	50S ribosomal protein L10	-	1.28
A0A0E1AFG8	Peptide deformylase	*def*	1.29
6PGD	6-phosphogluconate dehydrogenase, decarboxylating	*gnd*	1.29
SYI	Isoleucine--tRNA ligase	*ileS*	1.29
A0A0D1JI56	Cell division initiation protein DivIVA	*QU38_12295*	1.30
A0A077ULT9	Ribonuclease R	*rnr*	1.30
A0A077UNT4	YozC	*ERS140026_00525*	1.30
A0A0D6H2B5	Salicylate hydroxylase	*nagX*	1.31
A0A0D1HB86	Strain SA-120 Contig627, whole genome shotgun sequence	-	1.33
A0A0D6DL16	Virulence factor expression protein CvfB	*SAJPND1_01333*	1.33
A0A077W554	Membrane associated protein	*ERS140026_02425*	1.33
A0A0D1HXC4	Glycine-tRNA ligase	*glyQS*	1.34
A0A0D1HG99	Strain SA-120 Contig630, whole genome shotgun sequence	-	1.34
A0A0E1AIF5	2-amino-3-ketobutyrate coenzyme A ligase	*kbl*	1.34

Accession Number	Protein Name	Gene Name	Fold Change
A0A0D1GWI4	50S ribosomal protein L4	*rplD*	1.35
A0A077VD35	Multidrug resistance protein (Function not yet clear)	*ERS140254_01513*	1.35
A0A068A8I1	Serine/threonine protein kinase	*prkC*	1.36
A0A0B6XQ41	GMP reductase	*guaC*	1.36
A0A0E1AJI0	Ferredoxin-dependent glutamate synthase	*SAZ172_2563*	1.37
A0A0D1FKQ9	30S ribosomal protein S9	*rpsI*	1.38
HUTG	Formimidoylglutamase	*hutG*	1.38
A0A077UAU6	Transcription regulator (Contains diacylglycerol kinase catalytic domain)	*dagK*	1.38
A0A069FMB9	DNA-directed RNA polymerase subunit alpha	*rpoA*	1.38
A0A0D1H0K7	50S ribosomal protein L23	*rplW*	1.39
A0A0D1I822	30S ribosomal protein S20	-	1.39
A0A077UKA9	50S ribosomal protein L24	-	1.39
A0A077USL8	L-lactate permease	-	1.39
A0A090LY09	GTPase Obg	*obg*	1.40
A0A0D6DEH7	Dimethylallyltransferase	*SAJPND1_01475*	1.40
A0A0D6HUF4	Phosphoenolpyruvate-protein phosphotransferase	*ptsI*	1.40
A0A0B6XPZ7	Dihydrolipoyllysine-residue succinyltransferase component of 2-oxoglutarate dehydrogenase complex	*odhB*	1.40
A0A0D1JTN2	30S ribosomal protein S10	-	1.41
A0A033UV78	50S ribosomal protein L36	*rpmJ*	1.42
A0A090LYX3	Inosine-uridine preferring nucleoside hydrolase	*SAU060112_40119*	1.43
A0A090LXW9	Isocitrate dehydrogenase [NADP]	*icd*	1.44
A0A0D1I8Q0	Tyrosine-tRNA ligase	*tyrS*	1.44
DRP35	Lactonase drp35	*drp35*	1.44
A0A0D1FPR0	3-hydroxyacyl-[acyl-carrier-protein] dehydratase FabZ	*fabZ*	1.45
MURC	UDP-N-acetylmuramate-L-alanine ligase	*murC*	1.45
A0A090N1V7	Porphobilinogen deaminase	*hemC*	1.45
A0A0D1FSK9	GTP cyclohydrolase FolE2	*folE2*	1.46
A0A077UEX9	Putative cysteine ligase BshC	*bshC*	1.46
A0A0D1HTN7	50S ribosomal protein L18	-	1.46
A0A077VBV0	Methionine aminopeptidase	*map_3*	1.47
A0A0D1HQY5	Nucleoside diphosphate kinase	*ndk*	1.48
A0A077VA34	Uncharacterized protein	-	1.49
A0A0D1JN11	N utilization substance protein B homolog	*nusB*	1.49
A0A0D1FYV9	Glycerol-3-phosphate cytidylyltransferase	*QU38_10225*	1.49
A0A0B6XQX6	Bifunctional protein: zinc-containing alcohol dehydrogenase%3B quinone oxidoreductase (NADPH: quinone reductase)	*ERS094548_01069*	1.50
A0A0D1HPJ7	DNA-directed RNA polymerase subunit omega	*rpoZ*	1.50
A0A0D1IHK1	50S ribosomal protein L20	*rplT*	1.50

Accession Number	Protein Name	Gene Name	Fold Change
A0A0D1GZ06	Putative septation protein SpoVG	*spoVG*	1.50
A0A0D1I5C9	Strain SA-120 Contig629, whole genome shotgun sequence	-	1.50
A0A0D1IBM2	DEAD-box ATP-dependent RNA helicase CshA	*cshA*	1.50
A0A0D1I5P8	High-affinity zinc uptake system ATP-binding protein znuC	*SAJPND1_01510*	1.51
A0A090LY44	Alanine-tRNA ligase	*alaS*	1.51
A0A0C5HZK9	Glutamate-1-semialdehyde 2,1-aminomutase	*hemL*	1.52
A0A0D1J4E7	Glutamate dehydrogenase	*QU38_11405*	1.52
A0A0D1HB94	2,3-bisphosphoglycerate-independent phosphoglycerate mutase	*gpmI*	1.53
A0A0D1JM51	Strain SA-120 Contig627, whole genome shotgun sequence	-	1.53
A0A077UEE8	Proline--tRNA ligase	*proS*	1.54
A0A0D1J2L0	Phosphomethylpyrimidine kinase	*SAJPND1_00574*	1.55
A0A0B6XLK3	Haloacid dehalogenase-like hydrolase	*ppaX*	1.56
A0A0E1APW1	1-pyrroline-5-carboxylate dehydrogenase	*rocA*	1.57
A0A0E0VNX4	Ribosome-associated factor Y	*ST398NM01_0828*	1.57
A0A0D1H8Z3	3-oxoacyl-[acyl-carrier-protein] synthase 3	*fabH*	1.58
A0A090M2G5	Cobalamin biosynthesis CobT VWA domain protein	*SAU060112_70100*	1.59
A0A077W130	Iron-sulfur cluster assembly ATPase SufC	*yurY*	1.60
A0A0D1JQF8	NADH dehydrogenase	*QU38_11320*	1.61
A0A090LRA1	Uncharacterized protein	-	1.61
A0A0D1IW07	Histidine-tRNA ligase	*hisS*	1.61
A0A090N2E5	Mannitol-specific phosphotransferase enzyme IIA component	*mtlF*	1.62
A0A077VNY2	Teichoic acids export ATP-binding protein TagH	*tagH_1*	1.63
A0A0B6XQV6	Iron-sulfur cluster assembly/repair protein ApbC	*apbC*	1.63
A0A0D1I3J5	Urease accessory protein UreE	*ureE*	1.64
A0A090LT64	Uncharacterized protein	-	1.64
A0A0D1JWV4	50S ribosomal protein L28	*rpmB*	1.65
A0A0D1HYX5	50S ribosomal protein L14	*rplN*	1.65
A0A0D1J1L9	Phosphoglycerate kinase	*pgk*	1.65
A0A0D1JTT1	AspS protein	*aspS*	1.68
A0A0D1IQB7	Enoyl-[acyl-carrier-protein] reductase [NADPH]	*QU38_11665*	1.68
A0A077U1H0	FemB%2C factor involved in methicillin resistance/Glycine interpeptide bridge formation	*femB*	1.68
A0A0D1JTP6	Putative cytosolic protein	*QU38_03765*	1.68
A0A0D6DNQ3	Transcription accessory protein	*SAJPND1_02036*	1.69
A0A0D1IXM8	Small heat shock protein	*QU38_07680*	1.70
A0A0D1GN84	Strain SA-120 Contig629, whole genome shotgun sequence	-	1.70

Accession Number	Protein Name	Gene Name	Fold Change
A0A0D1HV16	Putative cytosolic protein	*QU38_12695*	1.70
A0A0D6DLY3	Pyrroline-5-carboxylate reductase	*proC*	1.71
A0A077UT21	Phage protein	*ERS140159_01807*	1.73
A0A077VJ87	GTP-binding protein TypA/BipA	*typA*	1.77
A0A0B6XQZ3	S-ribosylhomocysteine lyase	*luxS*	1.78
A0A090N1X3	Chaperone protein DnaK	*dnaK*	1.79
A0A0D1JX26	Glutamine synthetase	*glnA*	1.79
A0A0D6H5T9	NAD-dependent protein deacetylase	*cobB*	1.79
A0A0D1HD79	tRNA binding domain protein	*SAJPND1_01690*	1.81
SYL	Leucine-tRNA ligase	*leuS*	1.81
A0A0D1I2B3	Chaperone protein DnaJ	*dnaJ*	1.81
A0A077UA73	RNA binding protein%2C contains ribosomal protein S1 domain	*yugI_2*	1.84
A0A0D6GZ63	Phage protein	*ERS445051_01398*	1.88
A0A077UW75	YceI-like domain protein	*ERS140266_00175*	1.90
A0A0B6XSJ7	Alcohol dehydrogenase	*curA*	1.94
A0A0C5IA88	Formate-tetrahydrofolate ligase	*fhs*	1.95
A0A0B4N811	3-hexulose-6-phosphate synthase	*CH51_02975*	1.96
A0A0E1X668	2-oxoglutarate ferredoxin oxidoreductase subunit beta	*HMPREF0769_12488*	2.00
A0A0D1FVH2	Strain SA-120 Contig630, whole genome shotgun sequence	-	2.01
A0A0C5HEJ3	Endoribonuclease L-PSP	*yabJ*	2.02
A0A0D6HFI7	Succinyl-diaminopimelate desuccinylase	*dapE*	2.02
A0A0E1AHK4	Foldase protein PrsA	*prsA*	2.03
A0A0D1FGD6	Strain SA-120 Contig629, whole genome shotgun sequence	-	2.04
A0A090LYE2	Aminomethyltransferase	*gcvT*	2.04
A0A090LXA3	Lipase/esterase LipA	*SAU060112_10612*	2.06
A0A0D1GP04	Succinyl-CoA ligase [ADP-forming] subunit alpha	*SAJPND1_01177*	2.10
A0A0D1HWG4	ProteaseI	*SAJPND1_01839*	2.20
A0A090LXH5	3-hexulose-6-phosphate isomerase	*hxlB*	2.28
A0A0E1VP50	Aldehyde dehydrogenase (NAD) family protein	*HMPREF0776_0903*	2.41
A0A077V9E3	Acetyltransferase	*ERS140254_00101*	2.47
A0A0D6GUT4	Membrane protein	*mmpL8*	2.48
A0A0E1VYJ0	Urease accessory protein UreG	*ureG*	2.63
A0A0D6DK60	Uncharacterized protein	-	2.65
A0A0B6XST7	Urease subunit alpha	*ureC*	2.73
A0A0B6XR80	Glyoxylate reductase/Glyoxylate reductase/Hydroxypyruvate reductase	*SASCBU26_02308*	3.00
A0A0D6DN74	Aldehyde dehydrogenase	*SAJPND1_02098*	3.44
A0A0B6XR64	Urease subunit beta	*ureB*	3.76
A0A0D1IUM8	L-serine dehydratase beta subunit	*SAJPND1_02531*	4.35

**Accession Number**	**Protein Name**	**Gene Name**	**Fold Change**
A0A0D1H3E1	Sced	*SAJPND1_02072*	4.71
