# Supplementary material for: iTRAQ-Based Quantitative Proteomic Profiling of Staphylococcus aureus Under Different Osmotic Stress Conditions
Source: Front Microbiol. 2019 May 29;10:1082. doi: 10.3389/fmicb.2019.01082 (PMC6549500; doi:10.3389/fmicb.2019.01082)
Supplement: Supplementary file 7 [file Data_Sheet_7.PDF]

Table S7 The upregulated proteins in both the 10% NaCl group compared with the control group and in the 20% NaCl group compared with the 10% NaCl group.

**Accession Number**	**Protein Name**	**Gene Name**	**Fold Change**	
			**10% NaCl ↑**	**20% NaCl ↑**
A0A077W129	30S ribosomal protein S13	*rpsM*	1.20	1.22
A0A0D1FKQ9	30S ribosomal protein S9	*rpsI*	1.20	1.38
A0A0D1H0K7	50S ribosomal protein L23	*rplW*	1.21	1.39
A0A0B6XLK3	Haloacid dehalogenase-like hydrolase	*ppaX*	1.22	1.56
A0A0D1H8Z3	3-oxoacyl-[acyl-carrier-protein] synthase 3	*fabH*	1.23	1.58
A0A0D1HQY5	Nucleoside diphosphate kinase	*ndk*	1.24	1.48
A0A0B6XQS8	Valine-tRNA ligase	*valS*	1.24	1.26
A0A0D1I3J5	Urease accessory protein UreE	*ureE*	1.25	1.64
A0A0D1HYX5	50S ribosomal protein L14	*rplN*	1.25	1.65
A0A090N2E5	Mannitol-specific phosphotransferase enzyme IIA component	*mtlF*	1.28	1.62
A0A0D1GWI4	50S ribosomal protein L4	*rplD*	1.28	1.35
A0A0D1IW07	Histidine-tRNA ligase	*hisS*	1.29	1.61
A0A0D1IHK1	50S ribosomal protein L20	*rplT*	1.33	1.50
A0A090LYX3	Inosine-uridine preferring nucleoside hydrolase	*SAU060112_40119*	1.42	1.43
A0A0D1JWV4	50S ribosomal protein L28	*rpmB*	1.55	1.65
A0A0D1GP04	Succinyl-CoA ligase [ADP-forming] subunit alpha	*SAJPND1_01177*	1.49	2.10
A0A0D6GUT4	Membrane protein	*mmpL8*	1.55	2.48
A0A0D1H3E1	Sced	*SAJPND1_02072*	1.75	4.71
A0A0B6XR64	Urease subunit beta	*ureB*	1.75	3.76
A0A090LXA3	Lipase/esterase LipA	*SAU060112_10612*	1.80	2.06
