# Supplementary material for: iTRAQ-Based Quantitative Proteomic Profiling of Staphylococcus aureus Under Different Osmotic Stress Conditions
Source: Front Microbiol. 2019 May 29;10:1082. doi: 10.3389/fmicb.2019.01082 (PMC6549500; doi:10.3389/fmicb.2019.01082)
Supplement: Supplementary file 8 [file Data_Sheet_8.PDF]

Table S8 The upregulated proteins in the 10% NaCl group compared with the control group, and the downregulated proteins in the 20% NaCl group compared with the 10% NaCl group.

**Accession Number**	**Protein Name**	**Gene Name**	**Fold Change**	
			**10% NaCl ↑**	**20% NaCl ↓**
A0A0B6XRB8	Sodium export permease protein	*yhaP*	1.20	0.67
A0A0B6XLD2	Sporulation protein	*yaaT*	1.21	0.74
A0A0E0VM51	Acetate CoA-transferase YdiF	*ST398NM01_0245*	1.25	0.64
A0A0E1AHK5	Acetylornithine deacetylase/Succinyl-diaminopimelate desuccinylase-related deacylase	*SAZ172_1765*	1.30	0.77
A0A0C2HDA0	Glycerol-3-phosphate dehydrogenase	*glpD*	1.33	0.76
A0A077V1V2	50S ribosomal protein L10	*rplJ*	1.33	1.28
A0A0B6XPL1	Aldo/keto reductase family protein	*yvgN*	1.36	0.41
A0A0C5HKH2	PTS-dependent dihydroxyacetone kinase phosphotransfer protein	*dhaM*	1.40	0.52
A0A0D1FBM5	6-phosphogluconolactonase	*SAJPND1_01893*	1.40	0.64
A0A077W1K8	Pyrrolidone-carboxylate peptidase	*pcp*	1.40	0.64
A0A0D6DDB0	Dihydrofolate reductase	*SAJPND1_01369*	1.41	0.59
A0A033UV78	50S ribosomal protein L36	*rpmJ*	1.44	1.42
A0A0D1HN41	Phosphoenolpyruvate carboxykinase [ATP]	*pckA*	1.51	0.58
A0A069FZ92	Branched-chain-amino-acid aminotransferase	*ilvE*	1.51	0.55
A0A0D1EZR2	Strain SA-120 Contig627, whole genome shotgun sequence	*QU38_08415*	1.51	0.74
A0A0D6DD31	4-hydroxy-tetrahydrodipicolinate synthase	*dapA*	1.65	0.63
A0A0B6XPZ7	Dihydrolipoyllysine-residue succinyltransferase component of 2-oxoglutarate dehydrogenase complex	*odhB*	1.65	1.42
A0A077ULL5	LPXTG surface protein	*sdrD*	1.70	0.76
A0A0D1I822	30S ribosomal protein S20	*rpsT*	1.72	1.39
A0A0D6HMU5	FmhA protein of FemAB family	*fmhA*	1.76	0.74
A0A0D6FMN0	Putative cytosolic protein	*ERS445051_00083*	1.78	0.40
A0A0D1H1Q3	Glyoxalase family protein	*QU38_12370*	1.78	0.71
A0A0D1JMR1	Acyl carrier protein	*acpP*	1.83	0.68
A0A077USG2	Immunodominant staphylococcal antigen A	*isaA*	1.83	0.47
A0A068A921	Nitrate reductase	*narH*	1.85	0.30

Accession Number	Protein Name	Gene Name	Fold Change	
			10% NaCl ↑	20% NaCl ↓
A0A0D6DE64	Putative cytosolic protein	*SAJPND1_01441*	1.90	0.42
A0A090N225	Secretory antigen	*ssaA*	1.95	0.53
A0A0B6XQV4	Alpha-amylase	*malA*	2.00	0.71
MURC	UDP-N-acetylmuramate-L-alanine ligase	*murC*	2.02	1.45
A0A0D1INB5	Arginine deiminase	*arcA*	2.07	0.16
A0A0D6HFI7	Succinyl-diaminopimelate desuccinylase	*dapE*	2.07	2.02
A0A077U2I8	FMN-dependent NADH-azoreductase	*azoR*	2.10	0.38
A0A077VA34	Uncharacterized protein	*ERS140026_01689*	2.28	1.49
A0A0D1FZV0	Strain SA-120 Contig626, whole genome shotgun sequence	*QU38_07650*	2.28	0.58
A0A077UT21	Phage protein	*ERS140159_01807*	2.40	1.73
A0A0B4N811	3-hexulose-6-phosphate synthase	*CH51_02975*	2.40	1.96
A0A0D1I4N9	Strain SA-120 Contig630, whole genome shotgun sequence	*QU38_15895*	2.41	0.44
A0A0D1HX32	Strain SA-120 Contig626, whole genome shotgun sequence	*QU38_07375*	2.43	0.58
A0A0D1HYD3	Strain SA-120 Contig626, whole genome shotgun sequence	*QU38_07345*	2.46	0.23
A0A077UBW6	O-methyltransferase family protein	*ERS140162_01570*	2.55	0.78
A0A0D1IPY9	Organic hydroperoxide resistance protein	*SAJPND1_00812*	2.56	1.22
A0A0D1K2T0	Putative cytosolic protein	*QU38_07185*	2.64	0.65
A0A0C5HEJ3	Endoribonuclease L-PSP	*yabJ*	2.85	2.02
A0A0D1K3I9	Cold shock protein	*QU38_11035*	2.89	0.28
A0A0D1HB94	2,3-bisphosphoglycerate-independent phosphoglycerate mutase	*gpmI*	2.90	1.53
A0A077UW75	YceI-like domain protein	*ERS140266_00175*	3.00	1.90
A0A090LVJ3	Ornithine carbamoyltransferase	*arcB*	3.09	0.14
A0A0D1HYC8	Nitrite reductase [NAD(P)H] large subunit	*SAJPND1_02388*	3.17	0.19
A0A0E1VTG8	Oxidoreductase, FAD/FMN-binding protein	*HMPREF0776_1945*	3.20	0.83
A0A0D3Q462	Uncharacterised protein	*CH51_01290*	3.31	0.63
A0A0D1H4E0	Strain SA-120 Contig630, whole genome shotgun sequence	*QU38_15005*	3.40	0.44

Accession Number	Protein Name	Gene Name	Fold Change	
			10% NaCl ↑	20% NaCl ↓
A0A0E1X586	Phage major tail protein	*HMPREF0769_12240*	3.50	0.75
A0A033V5W3	Cold shock protein CspA	*cspA*	3.75	0.70
A0A0B6XQZ3	S-ribosylhomocysteine lyase	*luxS*	3.80	1.78
A0A077UAB5	D-2-hydroxyacid dehydrogenase	*ldhD_2*	4.25	0.47
A0A0D1JZ55	Putative cytosolic protein	*SAJPND1_01294*	4.52	0.72
A0A0B6XLS6	UDP-2-acetamido-2%2C6-dideoxy-beta-L-talose 4-dehydrogenase	*ERS094548_01225*	5.15	0.55
A0A077UNT4	YozC	*ERS140026_00525*	6.35	1.30
A0A090N1L9	UDP-glucose 4-epimerase	*capD*	7.55	0.62
