# Supplementary material for: iTRAQ-Based Quantitative Proteomic Profiling of Staphylococcus aureus Under Different Osmotic Stress Conditions
Source: Front Microbiol. 2019 May 29;10:1082. doi: 10.3389/fmicb.2019.01082 (PMC6549500; doi:10.3389/fmicb.2019.01082)
Supplement: Supplementary file 9 [file Data_Sheet_9.PDF]

Table S9 The downregulated proteins in the 10% NaCl group compared with the control group, and the upregulated proteins in the 20% NaCl group compared with the 10% NaCl group.

**Accession Number**	**Protein Name**	**Gene Name**	**Fold Change**	
			**10% NaCl** ↓	**20% NaCl** ↑
A0A0E1AHK4	Foldase protein PrsA	*prsA*	0.44	2.03
A0A0E1AE25	Phosphoglycolate phosphatase	*SAZ172_0574*	0.45	1.20
A0A0D1IXM8	Small heat shock protein	*QU38_07680*	0.48	1.70
A0A090LY09	GTPase Obg	*obg*	0.50	1.40
A0A090LZA4	Chorismate mutase I/2-keto-3-deoxy-D-arabino-heptulosonate-7-phosphate synthase I beta%2C AroH/AroA I beta	*aroA*	0.52	1.24
A0A090N1X3	Chaperone protein DnaK	*dnaK*	0.58	1.79
A0A077W554	Membrane associated protein	*ERS140026_02425*	0.60	1.33
A0A0D6H2B5	Salicylate hydroxylase	*nagX*	0.68	1.31
A0A0D1FPR0	3-hydroxyacyl-[acyl-carrier-protein] dehydratase FabZ	*fabZ*	0.72	1.45
A0A0B6XQX6	Bifunctional protein: zinc-containing alcohol dehydrogenase%3B quinone oxidoreductase (NADPH:quinone reductase)	*ERS094548_01069*	0.73	1.50
A0A090LZZ7	Uncharacterized peptidase YqhT	*YqhT*	0.76	1.24
A0A0D1IBM2	DEAD-box ATP-dependent RNA helicase CshA	*cshA*	0.77	1.50
A0A0D6H5T9	NAD-dependent protein deacetylase	*cobB*	0.77	1.79
A0A0D6DLY3	Pyrroline-5-carboxylate reductase	*proC*	0.78	1.71
A0A0D6DFF7	Uncharacterized protein	*SAJPND1_01888*	0.80	1.21
A0A0C5I2R4	Peptidase propeptide and YPEB domain-containing protein	*ERS445051_01691*	0.80	1.25
A0A0D1HD79	tRNA binding domain protein	*SAJPND1_01690*	0.80	1.81
A0A0D1I2B3	Chaperone protein DnaJ	*dnaJ*	0.80	1.81
A0A0D1HV16	Putative cytosolic protein	*QU38_12695*	0.81	1.70
A0A077ULT9	Ribonuclease R	*rnr*	0.83	1.30
