# Supplementary material for: iTRAQ-Based Quantitative Proteomic Profiling of Staphylococcus aureus Under Different Osmotic Stress Conditions
Source: Front Microbiol. 2019 May 29;10:1082. doi: 10.3389/fmicb.2019.01082 (PMC6549500; doi:10.3389/fmicb.2019.01082)
Supplement: Supplementary file 10 [file Data_Sheet_10.PDF]

Table S10 The downregulated proteins in both the 10% NaCl group compared with the control group and in the 20% NaCl group compared with the 10% NaCl group.

Accession Number	Protein Name	Gene Name	Fold Change	
			10% NaCl ↓	20% NaCl ↓
A0A0D1IXJ7	Gamma-hemolysin B subunit HlgB	*SAJPND1_02412*	0.08	0.52
A0A0D6DGW3	Gamma-hemolysin A subunit HlgA	*SAJPND1_02409*	0.11	0.62
OMP7	77 kDa membrane protein	*SACOL2002*	0.12	0.33
SBI	Immunoglobulin-binding protein sbi	*sbi*	0.22	0.50
A0A0D6DFD4	Beta-lactamase regulator protein	*SAJPND1_01896*	0.22	0.50
A0A0D1JXR9	Strain SA-120 Contig630, whole genome shotgun sequence	*QU38_15890*	0.15	0.57
A0A0C5HVE0	Pyruvate decarboxylase%3B Alpha-keto-acid decarboxylase	*ipdC*	0.22	0.50
A0A0D1I054	L-lactate dehydrogenase	*ldh*	0.25	0.50
A0A090N157	Alpha-hemolysin	*hly*	0.28	0.42
ADH	Alcohol dehydrogenase	*adh*	0.29	0.50
A0A0D6DIM1	Ribokinase	*SAJPND1_00306*	0.29	0.29
A0A077U1D0	Amino acid permease	*steT*	0.30	0.23
A0A0B6XPE8	Pyruvate carboxylase	*cfiB_1*	0.30	0.33
A0A077VAK0	Hexose phosphate transport protein	*uhpT*	0.30	0.67
A0A077V7K3	ABC transporter substrate-binding protein	*psaA*	0.33	0.70
A0A0C2HZY8	Pyrimidine-nucleoside phosphorylase	*pdp*	0.33	0.67
A0A0D1GJW4	Arsenate reductase family protein	*QU38_11110*	0.33	0.67
A0A0D1JQX0	Strain SA-120 Contig628, whole genome shotgun sequence	*QU38_11030*	0.35	0.47
Q9S2Z4	Cell surface protein map-w	*map-w*	0.35	0.75
A0A090LW57	ABC transporter, permease protein	*SAU060112_100003*	0.36	0.50
A0A0B6XTB4	N-acetylmuramoyl-L-alanine amidase%2C family 4	*ERS094548_01923*	0.39	0.55
A0A0D1G5I0	Strain SA-120 Contig630, whole genome shotgun sequence	*QU38_15345*	0.40	0.47
A0A0D6GIM9	Autolysin	*atl_1*	0.40	0.50
A0A077UDD9	3-ketoacyl-CoA thiolase%3B Acetyl-CoA acetyltransferase	*ERS140248_02364*	0.40	0.75
A0A077VSV4	Peptide ABC transporter permease	*oppB_1*	0.40	0.75
A0A0D1GTD3	Strain SA-120 Contig622, whole genome shotgun sequence	*QU38_05245*	0.42	0.56
A0A0D6DDK7	Uncharacterized protein	*SAJPND1_01250*	0.42	0.46
A0A0D1HUP1	Nucleoside transporter	*QU38_09615*	0.43	0.67

Accession Number	Protein Name	Gene Name	Fold Change	
			10% NaCl ↓	20% NaCl ↓
A0A090M2J3	Uncharacterized protein	*SAU060112_70120*	0.44	0.59
A0A0D1HQ98	Histidine kinase	*QU38_10565*	0.45	0.64
A0A0D1JLL3	Adenosylmethionine-8-amino-7-oxononanoate aminotransferase	*bioA*	0.45	0.61
A0A0D1K2N9	GlnQ protein	*glnQ*	0.47	0.47
A0A0B6XP33	Oligopeptide transport ATP-binding protein oppD	*oppD_1*	0.47	0.44
A0A068W8S1	Ribosomal RNA large subunit methyltransferase H	*orfX*	0.50	0.60
A0A090LVY0	Aspartate carbamoyltransferase	*pyrB*	0.50	0.60
A0A0B6XKT1	Aldehyde-alcohol dehydrogenase	*adhE*	0.50	0.60
A0A077V9M9	Response regulator SaeR	*saeR*	0.50	0.62
A0A0D1JT33	Cardiolipin synthase	*QU38_06435*	0.50	0.65
A0A077TYE7	Uncharacterized protein conserved in bacteria	*ERS140095_00284*	0.50	0.71
A0A090M2D6	Oligoendopeptidase F	*SAU060112_70075*	0.50	0.83
A0A0B6XNE5	Hydrolase (HAD superfamily)	*ERS094548_00694*	0.52	0.66
A0A0D6FS99	Putative polyribitolphosphotransferase	*tagF_1*	0.52	0.47
A0A077UDM1	Manganese ABC transporter%2C ATP-binding protein SitB	*sitB*	0.54	0.47
A0A0D1JGI6	2-C-methyl-D-erythritol 4-phosphate cytidylyltransferase	*ispD*	0.56	0.67
A0A0B6XMG3	Argininosuccinate synthase	*argG*	0.57	0.25
A0A0D1GBS9	Signal transduction protein TRAP	*SAJPND1_01796*	0.57	0.63
A0A0D1G1J9	Cytochrome c oxidase polypeptide II	*SAJPND1_00987*	0.58	0.56
A0A077TZL7	Modification methylase MboII	*mboIIM*	0.58	0.74
A0A090LW12	Poly (Glycerol-phosphate) alpha-glucosyltransferase	*SAU060112_10705*	0.58	0.60
A0A090LXY5	Uncharacterized protein	*SAU060112_120007*	0.58	0.63
A0A0D1GVJ7	Poly (Glycerol-phosphate) alpha-glucosyltransferase	*SAJPND1_00558*	0.59	0.56
A0A090LR26	Uncharacterized oxidoreductase YcsN	*ycsN*	0.60	0.68
A0A0E1VKR4	ABC transporter, ATP-binding protein	*HMPREF0776_1978*	0.60	0.32
A0A0D1IUP6	Strain SA-120 Contig628, whole genome shotgun sequence	*QU38_10035*	0.60	0.77
A0A090LWI3	Argininosuccinate lyase	*argH*	0.60	0.25

Accession Number	Protein Name	Gene Name	Fold Change	
			10% NaCl ↓	20% NaCl ↓
A0A077TYC4	Similar to putative sodium/glucose cotransporter	*sglT*	0.60	0.50
A0A090M057	Putative ATP-dependent helicase DinG homolog	*dinG*	0.60	0.67
A0A0D1I5J3	Ribulose-phosphate 3-epimerase	*QU38_12445*	0.60	0.83
A0A0B6XKT2	Ribose operon repressor%2C putative	*degA*	0.60	0.78
A0A090M1C4	Dihydroorotate dehydrogenase (quinone)	*pyrD*	0.61	0.51
A0A0D6GYJ2	Alanine dehydrogenase	*ald1*	0.61	0.18
A0A0B6XNT6	Bifunctional ligase/repressor BirA	*birA*	0.64	0.72
A0A0D1J363	Uracil phosphoribosyltransferase	*upp*	0.64	0.83
A0A0B6XNX0	Alanine dehydrogenase	*ald2_1*	0.64	0.39
A0A077U2Q8	Quinol oxidase polypeptide I QoxB	*qoxB*	0.67	0.50
A0A0D1GC10	Strain SA-120 Contig629, whole genome shotgun sequence	*QU38_12985*	0.67	0.58
A0A0D1K8W2	Bifunctional protein PyrR	*pyrR*	0.70	0.67
A0A0C5HMT4	GntR family transcriptional regulator	*treR_1*	0.70	0.45
A0A0D1H6Z1	Nitric oxide synthase oxygenase	*QU38_06960*	0.70	0.43
A0A0E1AE46	Phosphoenolpyruvate-dihydroxyacetone phosphotransferase, dihydroxyacetone binding subunit DhaK	*SAZ172_0661*	0.70	0.57
A0A077UP25	Membrane-bound serine protease	*ERS140159_00273*	0.70	0.71
A0A069FW70	Fructose-1,6-bisphosphatase class 3	*fbp*	0.70	0.75
A0A0B6XRK6	Nicotinate phosphoribosyltransferase	*ERS094548_02369*	0.71	0.63
A0A0D6DPK7	Staphyloxanthin biosynthesis protein CrtP	*SAJPND1_02566*	0.73	0.71
A0A0B6XPW9	Sodium/proline symporter	*putP*	0.73	0.54
A0A077UUV1	Hydrogen peroxide-inducible genes activator	*oxyR*	0.73	0.74
A0A090LUF4	Ribose-5-phosphate isomerase A	*rpiA*	0.74	0.72
A0A090LVR9	Uncharacterized protein	*SAU060112_10595*	0.76	0.72
A0A0D1IBW0	Strain SA-120 Contig620, whole genome shotgun sequence	*QU38_04480*	0.77	0.65
A0A0D1G9T6	Tautomerase	*QU38_13190*	0.77	0.59
A0A0D1HWP8	Dephospho-CoA kinase	*coaE*	0.78	0.80
A0A0D1FJV3	Glucose-6-phosphate 1-dehydrogenase	*zwf*	0.79	0.82
A0A0D3Q7P5	3-phosphoshikimate 1-carboxyvinyltransferase	*aroA_2*	0.79	0.75

Accession Number	Protein Name	Gene Name	Fold Change	
			10% NaCl ↓	20% NaCl ↓
A0A090LXB7	Luciferase-like monooxygenase	*SAU060112_20273*	0.79	0.66
A0A090LVS1	Potassium-transporting ATPase B chain	*kdpB*	0.80	0.50
A0A0D6HWL4	ATP-dependent helicase/deoxyribonuclease subunit B	*addB*	0.80	0.49
A0A090N1M7	Type-1 restriction enzyme R protein	*hsdR*	0.80	0.63
A0A0D6DHW6	Glycosyltransferase	*SAJPND1_00237*	0.80	0.50
A0A0D6GTK5	3-hydroxy-3-methylglutaryl coenzyme A reductase	*mvaA*	0.83	0.72
A0A0E1XJY5	Abi-like protein	*HMPREF0769_10093*	0.83	0.46
