# Supplementary figures and images for: iTRAQ-Based Quantitative Proteomic Profiling of Staphylococcus aureus Under Different Osmotic Stress Conditions
Source: Front Microbiol. 2019 May 29;10:1082. doi: 10.3389/fmicb.2019.01082 (PMC6549500; doi:10.3389/fmicb.2019.01082)

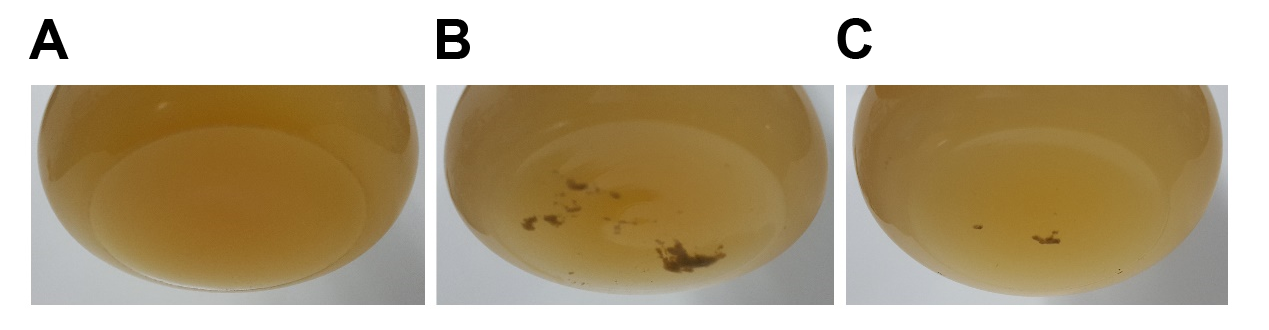

Supplement: Supplementary file 12 [file Image_1.TIF]
